# Drug repurposing for ligand-induced rearrangement of Sirt2 active site-based inhibitors via molecular modeling and quantum mechanics calculations

**DOI:** 10.1038/s41598-021-89627-0

**Published:** 2021-05-13

**Authors:** Shiv Bharadwaj, Amit Dubey, Nitin Kumar Kamboj, Amaresh Kumar Sahoo, Sang Gu Kang, Umesh Yadava

**Affiliations:** 1 Department of Biotechnology, Institute of Biotechnology, College of Life and Applied Sciences, Yeungnam University, 280 Daehak-Ro, Gyeongsan, Gyeongbuk 38541 Republic of Korea; 2 Computational Chemistry and Drug Discovery Division, Quanta Calculus Pvt. Ltd., Kushinagar, 274203 India; 3 School of Physical Sciences, DIT University, Dehradun, UK 248001 India; 4 Department of Applied Sciences, Indian Institute of Information Technology Allahabad, Allahabad, Uttar Pradesh 211015 India; 5 Department of Physics, Deen Dayal Upadhyaya Gorakhpur University, Gorakhpur, India

**Keywords:** Computational models, Computational biology and bioinformatics, Drug discovery, Molecular medicine

## Abstract

Sirtuin 2 (Sirt2) nicotinamide adenine dinucleotide-dependent deacetylase enzyme has been reported to alter diverse biological functions in the cells and onset of diseases, including cancer, aging, and neurodegenerative diseases, which implicate the regulation of Sirt2 function as a potential drug target. Available Sirt2 inhibitors or modulators exhibit insufficient specificity and potency, and even partially contradictory Sirt2 effects were described for the available inhibitors. Herein, we applied computational screening and evaluation of FDA-approved drugs for highly selective modulation of Sirt2 activity via a unique inhibitory mechanism as reported earlier for SirReal2 inhibitor. Application of stringent molecular docking results in the identification of 48 FDA-approved drugs as selective putative inhibitors of Sirt2, but only top 10 drugs with docking scores > − 11 kcal/mol were considered in reference to SirReal2 inhibitor for computational analysis. The molecular dynamics simulations and post-simulation analysis of Sirt2-drug complexes revealed substantial stability for Fluphenazine and Nintedanib with Sirt2. Additionally, developed 3D-QSAR-models also support the inhibitory potential of drugs, which exclusively revealed highest activities for Nintedanib (pIC50 ≥ 5.90 µM). Conclusively, screened FDA-approved drugs were advocated as promising agents for Sirt2 inhibition and required in vitro investigation for Sirt2 targeted drug development.

## Introduction

Nicotinamide adenine dinucleotide (NADP^+^)-dependent protein deacetylases or Sirtuins represent a distinctive class of evolutionarily conserved enzymes from bacteria to humans that are essentially required in the several vital functions of the biological system^1^. Primarily, Sirtuins were defined as deacetylases but recent studies have associated their biological function to catalyze post-translational modifications, including desuccinylation^2^ or ADP-ribosylation^3^, and demyristoylation^4,5^. In the human genome, seven isotypes of Sirtuins (Sirt1-7) have been discovered which diverge in their subcellular localization, viz. nucleus (Sirt1, 6, 7), cytosol (Sirt2), or mitochondria (Sirt3, 4, 5), and specific catalytic activity^6^. Although Sirtuins were identified in the elimination of various acyl groups, such as acetyl, crotonyl, decanoyl, glutaryl, myristoyl, propionyl, palmitoyl, and succinyl, from the histones, and several other protein substrates, but each isoform of Sirtuins exhibits specific affinity for acyl-lysine substrates^7,8^. Besides, Sirtuins have been studied in the regulation of metabolic processes to stress responses—metabolic, oxidative, and genotoxic stress^9–11^, and implicated in aging-related diseases—neurodegeneration and metabolic disorders; and hence, considered as potential therapeutic targets^12,13^.

The human isotype Sirtuin 2 (Sirt2), predominantly located in the cytosol, is reported to transiently shuttle from the cytosol into the nucleus via a cell cycle‐dependent manner^14^. Thus, it mainly deacetylates both the cytoplasmic and nuclear proteins and acted as a major cell cycle regulator^15,16^, a cause of myelination^15^, a regulator of autophagy, and a suppressor of brain inflammation^17^. Additionally, Sirt2 is generally considered as a tumor suppressor^18–20^ but knockdown and blocking of Sirt2 were linked with broad anticancer effects via stimulating c-Myc degradation in human breast cancer cell lines^21^. Also, Sirt2 was noted to promote lipolysis and preclude mature adipocyte differentiation while linked with a variety of neurodegenerative diseases in humans^22^. Therefore, activation and inhibition of Sirt2 have been constantly targeted for small molecule therapeutic development in reference to the biology under analysis^22–25^. Recent studies have documented the extensive biochemistry of Sirt2 and the crystal structures of the catalytic domain from several human isotopes revealed the various stages of the catalytic cycle^26–29^. As members of the Sirtuins family vastly shared conserved residues and high structure similarity in the catalytic core, numerous small-molecule isotype-selective inhibitors have been reported^25,30–32^. For instance, documented first X-ray structure of Sirt2 co-crystallized with a selective ligand, i.e., SirReal2, inhibited Sirt2 via ligand-induced structural rearrangement in the active pocket of protein, and represents a novel approach to inhibit Sirt2^23^. However, all the available inhibitors are endowed with various limitations, including lack of potency and/or poor solubility^33^. Moreover, the development of novel selective and specific Sirt2 inhibitors for therapeutic application from bench to bedside is exceedingly time-consuming and expensive. In response, a comprehensive range of small molecules as drugs that are already in clinical applications for treating various diseases might specifically target Sirt2 directly; and hence, stimulate approaches in treatment for Sirt2 associated diseases such as cancer and neurodegeneration via an approach referred to as drug repurposing or repositioning. The process of extracting new applications for existing FDA-approved drugs as Sirt2 inhibitors can be simplified by utilizing computational modeling followed by disease-relevant phenotypic in vitro cell culture assays and in vivo animal studies, which allowed an unbiased approach to obtain successful drug candidates. Hence, to find the specific Sirt2 inhibitors from the FDA-approved drug database, we performed the high-throughput computational screening of FDA-approved drugs for Sirt2 inhibition by ligand-induced rearrangement of the active site. The screened drugs were then further evaluated using complex molecular simulations and quantum chemical calculations to identify the specific and selective putative inhibitors for Sirt2 inhibition.

## Materials and methods

### Protein crystal structure and ligands collection

Three-dimensional (3D) crystal structure of human Sirt2 co-crystallized with selective inhibitor, i.e., 2-[(4,6-dimethylpyrimidin-2-yl)sulfanyl]-*N*-[5-(naphthalen-1-ylmethyl)-1,3-thiazol-2yl] acetamide, also named SirReal2 inhibitor and which inhibited Sirt2 protein by induced rearrangements in the active site, resolved at 1.42 Å was collected as receptor from RCSB Protein Data Bank (URL: http://www.rcsb.org/; PDB ID: 4RMH)^23^. Besides, 2102 FDA-approved drugs as ligand library were downloaded from the ZINC database (URL: https://zinc.docking.org/)^34^.

### Molecular docking and docking validation

To check the binding affinity of FDA-approved drugs with the selective pocket of Sirt2, molecular docking between the 3D crystallography structure of Sirt2 and drugs was performed using default parameters in the Schrödinger suite 2019–2^35^. Briefly, the 3D structure of Sirt2 was initially preprocessed using the PRIME^36,37^ and Protein preparation Wizard^38^ tools in the Schrödinger suite 2019–2^35^. Herein, co-crystallized ligand and water molecules were deleted from the protein crystal structure while polar hydrogen atoms were included in the protein using the Protein preparation wizard with default parameters. Additionally, hydrogen-bonding network optimization, rotation of hydroxyl and thiol hydrogen atoms, generation of tautomerization and protonation forms of histidine (His) residues, and Chi ‘flip’ assignments for Asparagine (Asn), Glutamine (Gln), and His residues, and minimization of hydrogen atoms in altered species were also accomplished. Moreover, standard distance-dependent dielectric constant at 2.0 Å, which defines the electronic polarization and small backbone fluctuations in the receptor, and conjugated gradient protocol were considered for the subsequent restrained refinement of the receptor and convergence of hydrogen atoms with the maximum root-mean-square deviation (RMSD) at 0.3 Å under optimized potentials for liquid simulations (OPLS)-3e force field^39^.

Likewise, the FDA-approved drug library was also prepared for molecular docking using the LigPrep module under default parameters in the Maestro-Schrödinger suite 2019–2^35^. Briefly, each ligand in the FDA-approved drug library was treated with an OPLS-3e force field^39^ at neutral pH using Epik^40^. Also, for each ligand, desalt and tautomers generation were considered while stereoisomer computation was marked based on specific chiralities (vary other chiral centers) to generate at most 32 per ligand on the LigPrep interface. For the collection of ligand poses as selective inhibitors with an ability to imitate induced rearrangements in the active pocket of Sirt2 protein as reported for SirReal2 inhibitor^23^, the active site residues (Ile^93^, Phe^96^, Tyr^104^, Ile^118^, Phe^119^, Ala^135^, Leu^138^, Tyr^139^, Pro^140^, Phe^143^, Ile^169^, Asp^170^, Phe^190^, Leu^206^, Ile^232^, Val^233^, and Phe^234^) interacting with the co-crystallized ligand, viz. SirReal2 inhibitor, were considered for molecule docking of FDA-approved drug library under default parameters by extra precision (XP) protocol of GLIDE tool^41^ in Maestro-Schrödinger suite 2019–2^35,42^. Herein, the receptor was treated as a rigid entity, and ligands were considered as flexible to achieve the most favorable interaction profile with the residues in the selective pocket of Sirt2. Additionally, Coulombic, freezing rotatable bonds, hydrogen bond, hydrophobic contacts, polar interactions, the penalty for buried polar groups, metal binding, water desolvation energy, and binding affinity enriching interactions were considered in the GLIDE XP scoring protocol^41^. Furthermore, the SirReal2 inhibitor was also extracted from the crystal complex and re-docked in the same selective binding pocket to justify the docking method and marked as a reference complex for comparative docking analysis with the screened drugs.

### Binding pose and interaction profiling

Following molecular docking, binding conformations of drugs with the highest negative docking score corresponds to the least root-mean-square deviation (RMSD) values were extracted and analyzed for binding conformations by comparison to docked Sirt2-SirReal2 inhibitor complex using Quick Align tool in Maestro-Schrödinger suite 2020–2^43^. Also, molecular contact formation was monitored between the active residues and atoms of the respective ligands at a distance of 4 Å around the docked ligand with default parameters. Herein, intermolecular non-covalent interactions, such as hydrophobic interactions, hydrogen bonds, π–π interactions, π–cation interactions, positive interactions, negative interactions, glycine interactions, and formation of salt bridges, were noted for docked complexes under default parameters. Finally, both 3D and 2D docked poses of Sirt2 with ligands were generated using academic Maestro-Schrödinger suite 2020–2^43^.

### Molecular mechanics/generalized Born surface area calculation

To remove the false positive hits obtained from molecular docking analysis, the molecular mechanics generalized Born surface area (MM/GBSA) method was utilized to compute the binding free energy for each Sirt2-drug complex and reference complex, viz Sirt2-SirReal2 inhibitor. Equations (1)–(3) symbolize the mathematical representation for the various steps employed to compute the binding free energy and respective associated energy components.

1 $$\Delta {\text{G}}_{{{\text{Bind}}}} = \Delta {\text{G}}_{{{\text{Com}}}} - \left( {\Delta {\text{G}}_{Rec } + \Delta {\text{G}}_{Lig} } \right) = \Delta {\text{H}} - {\text{T}}\Delta {\text{S }} \approx { }\Delta {\text{E}}_{{{\text{MM}}}} + \Delta {\text{G}}_{{{\text{sol}}}} - {\text{T}}\Delta {\text{S,}}$$

2 $$\Delta {\text{E}}_{{{\text{MM}}}} = \Delta {\text{E}}_{{{\text{Int}}}} + \Delta {\text{E}}_{{\text{Ele }}} + \Delta {\text{E}}_{{{\text{vdW}}}} ,$$

3 $$\Delta {\text{G}}_{{{\text{Sol}}}} = \Delta {\text{G}}_{{{\text{Pol}}}} + \Delta {\text{G}}_{{\text{Nonpol }}},$$

where ΔG_Bind_ stands for binding free energy, ΔG_Com_ depicts the total free energy in protein–ligand complex, and ΔG_Rec_ + ΔG_Lig_ represents the sum of free-state energies of protein and ligand. Using the second law of thermodynamics, binding free energy (Δ*G*_Bind_) computed for protein–ligand complex can be marked as the total sum of the enthalpy part (Δ*H*) and the entropy part (− *T*Δ*S*) of the whole system, as given in Eq. (1). In the present study, entropy contribution to the total binding free energy for the respective protein–ligand complexes was dropped because of its high computational cost and relatively low contribution in net binding free energy as reported earlier^44^. Hence, the enthalpy of the whole system was assigned equivalent to the net binding free energy of the protein–ligand complex and expressed as the sum of molecular mechanical energy (ΔE_MM_) and solvation free energy (ΔG_Sol_). Characteristically, ΔE_MM_ represents the collection of the intermolecular energy (ΔE_Int_), the electrostatic energy (ΔE_Ele_), and the van der Waals interactions (ΔE_vdW_) while ΔG_Sol_ marked for the total sum of polar (ΔG_Pol_) and non-polar (ΔG_Nonpol_) energies of the whole system under consideration. Hence, the binding free energy was computed for each docked protein–ligand complex under default parameters using Prime MM/GBSA module in Maestro-Schrödinger suite 2019.2^35,37^, as reported earlier^45,46^.

### System setup and explicit solvent molecular dynamics simulation

The selected docked complexes were subjected to classical molecular dynamics (MD) simulation under Linux environment on HP Z2 Microtower workstation using academic Desmond v5.6^47^ module in Maestro-Schrödinger suite 2018–4^48^. Since the implicit solvent model has been predicted to compute less accurate results against explicit solvent^49^, hence, the MD simulation system was prepared with the explicit solvent model, i.e., TIP4P (transferable intermolecular potential-4 point), as an orthorhombic box (10 × 10 × 10 Å buffer) and amended with 0.15 M salt to present physiological conditions. The complete simulation system was then neutralized by the addition of counter sodium and chloride ions, where ions were positioned at a 20 Å distance from ligand in the simulation system. Following, the complete system was subjected to energy minimization with maximum iterations of 2000 and 1.0 kcal/mol/Å convergence threshold as default parameters using system minimization tool in Desmond-Maestro v11.8 interface. Finally, each docked complex was simulated for 100 ns interval under default parameters with OPLS-2005 force field, and a total of 10,000 frames were collected at 10 ps interval during the simulation interval using molecular dynamics tool of academic Desmond v5.6^47^ module in Maestro-Schrödinger suite 2018–4^48^.

### Post-molecular dynamics simulation calculations

After MD simulation, firstly last snapshots were extracted from each 100 ns MD trajectories and studied for change in ligand conformation in the selected pocket by comparison to the initial docked poses and Sirt2-SirReal2 inhibitor complex by utilizing superimpose module in Maestro-Schrödinger suite 2018–4^48^. Moreover, these trajectories were further studied for root-mean-square deviation (RMSD), root-mean-square fluctuation (RMSF), and protein–ligand interaction profiling with the aid of Simulation Interaction Diagram module of Desmond v5.6 module^47^ Maestro-Schrödinger suite 2018–4^48^.

#### Essential dynamics

Essential dynamics, in terms of principal component analysis (PCA), is a statistical approach based on the computation and diagonalization of the covariance matrix for the Carbon-alpha (Cα) atoms to identify the collective modes of fluctuations in the protein structure. These generated orthogonal vectors or eigenvectors with the largest eigenvalues are called principal components (PCs). This method helps to collect the concerted fluctuations linked with the largest atomic vibrations that are essentially required for the protein function^50,51^. Normally, > 90% of the demonstrated total atomic motions in the protein structure can be characterized by about 20% of the principal axes, i.e. eigenvectors of the covariance matrix^51^. Thus, essential dynamics computation on each MD trajectory was performed using Bio3d package^52^ under R environment^53^, where all the Cα atoms of protein structure in 10,000 frames generated during 100 ns MD simulation were aligned to the initial pose for the reduction in root mean square variances amongst the corresponding residues in the structure of the protein.

#### Post-simulation binding free energy calculation

Following 100 ns MD simulation of screened complexes of Sirt2-drugs, poses were extracted at every 10 ps from the last 10 ns simulation trajectory of each complex for end-point binding free energy calculation via Prime MMGBSA module of the MM/GBSA protocol in Schrödinger suite 2019–2^35^, as mentioned in section “Molecular mechanics/generalized Born surface area calculation”. Herein, net binding free energy was computed under default parameters on the extracted snapshots from respective simulated systems, where protein–ligand complexes were processed by removal of counter ions and solvent molecules (TIP4P)^46,54^.

#### Quantum mechanics/molecular mechanics binding energy calculation

The hybrid Quantum Mechanics/Molecular Mechanics (QM/MM) calculations offer the precise quantification of the electronic interaction energies and chemical reactivities, but its application is limited to a small set of atoms due to high computational costs^55^. In this method, the protein–ligand complex is usually divided into two or three layers and each layer is treated separately with different levels of computational chemistry methods. Typically, the catalytic part or inhibitor bound region is marked as the lower layer for QM treatment while the treatment of the higher layer (protein environment) is executed under computationally less expensive and efficient methods such as classical molecular mechanics (MM) methods^46,56^. Hence, hybrid QM/MM energy for the protein–ligand complex is denoted as the sum of high-level QM energy of ligand (E_QM_), low-level molecular mechanics energy of the protein (E_MM_), and the medium level interaction energy of ligand with protein (E_QM-MM_), mathematically represented by Eq. (4) ^46^.

4 $${\text{E}}_{{{\text{QM}}/{\text{MM}}}} = {\text{E}}_{{{\text{QM}}}} + {\text{E}}_{{{\text{MM}}}} + {\text{E}}_{{{\text{QM}} - {\text{MM}}}} .$$

The QM/MM binding energy (∆E_QM/MM_) was calculated on the last snapshot extracted from 100 ns MD simulation of respective complexes as the difference in the QM/MM energy of the complex (E_QM/MM_) and the sum of the individual components, viz. ligand (E_QM_) and protein (E_MM_) energies, as depicted in Eq. (5).

5 $$\Delta {\text{E}}_{{{\text{QM}}/{\text{MM}}}} = {\text{E}}_{{{\text{QM}}/{\text{MM}}}} - \left( {{\text{E}}_{{{\text{QM}}}} + {\text{E}}_{{{\text{MM}}}} } \right).$$

The ONIOM (Own N-layered Integrated molecular Orbital and MM) method^57^ with electronic embedding as implemented in Gaussian 03 was used to compute the hybrid QM/MM binding energy calculations^46,57,58^, where protein environment was treated with a universal molecular mechanics force field (UFF) while the ligand-bound region was treated with dispersion corrected WB97XD/6-31G** density functional method as reported earlier^46^. In present calculations, the entropic energy contributions in the QM/MM binding energy have been ignored because of its comparatively less contribution in net binding free energy^44,46^.

### 3D-Quantitative structure–activity relationship models

The three-dimensional QSAR (3D-QSAR) algorithms are used to quantitatively predict the activity of 3-dimensionally aligned compounds^59^; hence, 3D-QSAR models provide affluence data about the exact molecular attributes important for biological activity/nonactivity and served as a significant predictive tool in the drug discovery process^60^. Thus, a set of known selective inhibitors of Sirt2 protein with diverse structures was collected from literature with their respective biological half-maximal inhibitory concentration activity (IC50) values. Following, their 2D structures were sketched in 2D interaction module and saved as 3D conformations in Schrödinger suite 2019–2^35^ for extra precision docking in the selective pocket of Sirt2 as mentioned in the earlier section “Molecular docking and docking validation”. Following compounds with considerable docking scores were considered for 3D-QSAR modeling using two approaches, viz. Atom-Based 3D-QSAR^61^ and Field-Based 3D-QSAR models^62^ using Schrödinger suite 2019–2^35^. Briefly, all the selected inhibitors, including SirReal2 inhibitor, were aligned using the Ligand Alignment tool with automatic selection parameters and considered for 3D-QSAR modeling. In both the 3D-QSAR models, inhibitors were divided into two datasets of the training set (70%) and test set (30%) in random manner to evaluate the robustness of the developed models. Of note, in the Atom-Based 3D-QSAR model, each atom is exemplified by a sphere with the van der Waals radius, in accordance with the atom type designated to each atom. Also, training set molecules are included with a regular grid of cubes, where each cube is characterized with up to six “bits”, demonstrating six different classes of atoms^63^. The atom types are hydrogen-bond donor (D), hydrophobic or nonpolar (H), negative ionic (N), positive ionic (P), electron-withdrawing (includes hydrogen-bond acceptors, W), and miscellaneous (X)^61,64^. Whereas Gaussian-based field style with default parameters was applied for Field-Based 3D-QSAR model development, including five characteristics such as Gaussian steric, Gaussian electrostatic, Gaussian hydrophobic, Gaussian hydrogen-bond acceptor, and Gaussian hydrogen-bond donor^61,64,65^. Later, a total of five-component, i.e., Partial Least Square (PLS) factor, model with regression statistics was generated for each 3D-QSAR modeling approaches under default parameters, where a maximum number of PLS factors in each model was 1/5 of the total number of training set molecules with 1 Å length of the sides of cubic volume elements. Moreover, the biological inhibition activity (IC50) of the known selective inhibitors of Sirt2 was converted into the logarithmic pIC50 scale; defined as pIC50 = − Log10(IC50), where IC50 is the micromolar concentration of the inhibitors exhibiting 50% inhibition of Sirt2 protein. Following, the best 3D-QSAR model was validated based on the standard deviation (SD), regression coefficient (R^2^), root-mean-squared error (RMSE), cross-regression coefficient (Q^2^), and Pearson correlation coefficient (Pearson-r) value for the internal test data set. Finally, activities of the test compounds (FDA-approved drugs) were also determined keeping the best model from each 3D-QSAR model in Schrödinger suite 2019–2^61,62,64^.

## Results

### Extra precision docking and binding pose analysis

**Figure 1:**
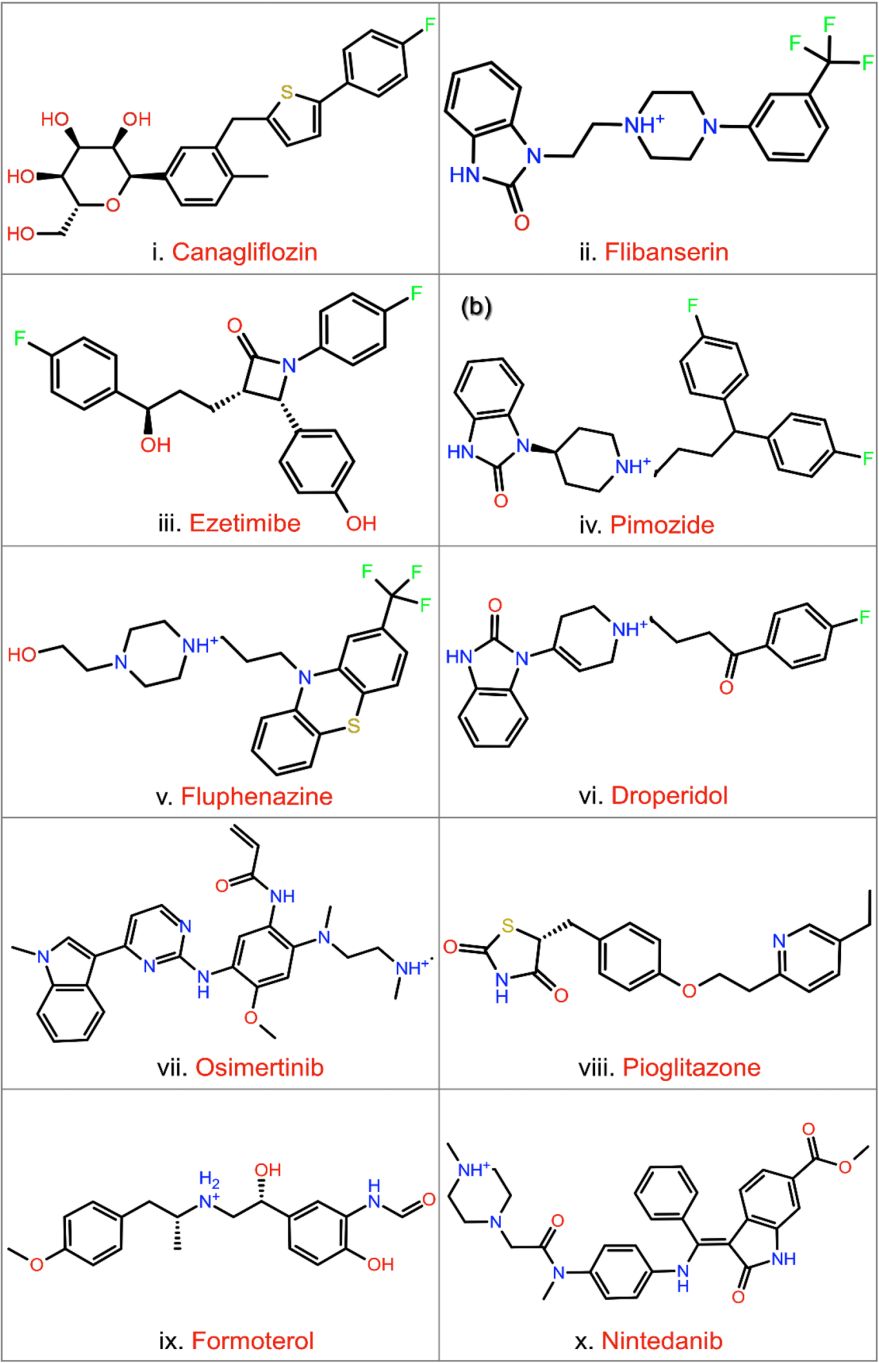
Structural formula and generic names for the potential FDA-approved drugs screened in the selective pocket of Sirt2 using the XP docking method. 2D images were sketched using academic Schrödinger-Maestro v12.4 suite^43^ (URL: https://www.schrodinger.com/freemaestro).

To discover the potential drugs with substantial binding affinity against the selective pocket of Sirt2, initially, the curated 2102 FDA-approved drug library was screened in the co-crystallized SirReal2 inhibitor binding pocket of Sirt2 crystal structure, and their binding affinities were accessed in terms of extra precision (XP) docking score corresponds to least docking RMSD value. This process results in the extraction of 43 drugs with a docking score between − 14.99 to − 2.91 kcal/mol in the selective pocket of Sirt2 (Supplementary Table S1). The list of screened drugs with ZINC ID, generic names, and targeted diseases is also mentioned in Supplementary Table S1. Based on the docking score, it can be suggested that screened drugs with docking scores > − 7 kcal/mol have an acceptable binding affinity towards active residues in the selective pocket of Sirt2. Hence, top ten drugs, viz. ZINC000043207238 (Canagliflozin), ZINC000052716421 (Flibanserin), ZINC000003810860 (Ezetimibe), ZINC000004175630 (Pimozide), ZINC000019203912 (Fluphenazine), ZINC000019796080 (Droperidol), ZINC000098023177 (Osimertinib), ZINC000000968326 (Pioglitazone), ZINC000000897256 (Formoterol), and ZINC000100014909 (Nintedanib), docked conformations with Sirt2 at highest docking score (> − 11 kcal/mol) and least RMSD values were marked as specific and selective putative inhibitors of Sirt2 (Fig. 1). Likewise, re-docking of SirReal2 inhibitor, as reference ligand (Supplementary Fig. S1), in the selective pocket of Sirt2 exhibited a significant docking score − 11.68 kcal/mol and intermolecular interactions as observed in the crystal structure^23^, these results support and validate the opted docking protocol for the identification of FDA-approved drugs as specific and selective inhibitors of Sirt2.

**Figure 2:**
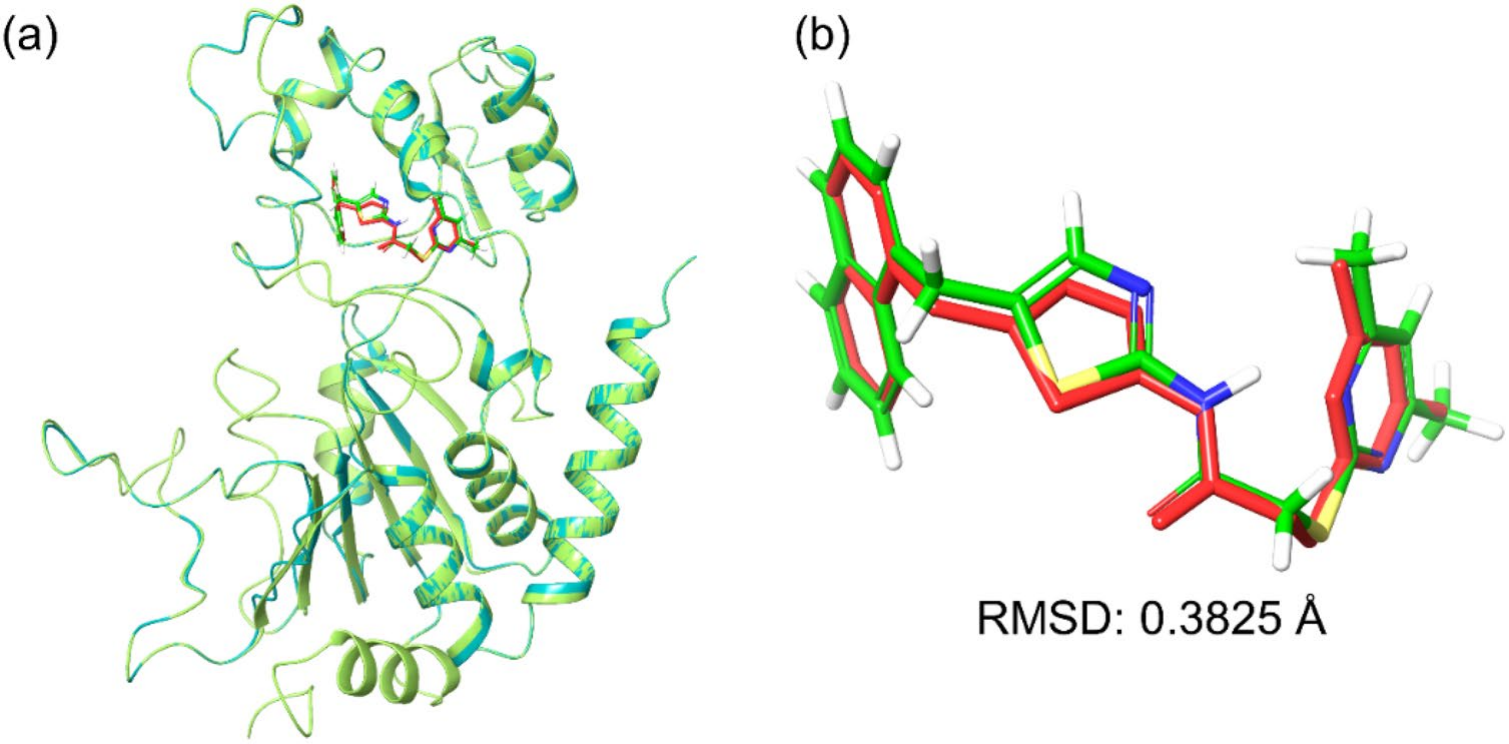
Superimposed molecular poses of reference docked complex Sirt2 (green color) with re-docked SirReal2 (green color) on co-crystallized SirReal2 inhibitor (red color) in the crystal structure of Sirt2 (cyan color). Here, complex alignment was performed with respect to ligand and position conformation of the aligned ligands was calculated in terms of RMSD values. Images were rendered using academic Schrödinger-Maestro v12.4 suite^43^ (URL: https://www.schrodinger.com/freemaestro).

To analyze the binding position occupied by the drugs in the docked complexes, initially, each docked pose of the screened ligands, i.e., FDA-approved drugs and re-docked SirReal2 inhibitor, were aligned to the native conformation of co-crystallized SirReal2 in the crystal structure of Sirt2. Remarkably, all the screened drugs showed residence in the selective binding cavity of Sirt2 structure with < 10 Å RMSD aligned to co-crystallized SirReal2 inhibitor conformation (Supplementary Fig. S2). Besides, re-docking of reference ligand, i.e., SirReal2 inhibitor, also demonstrated 0.3825 Å RMSD aligned to its co-crystallized conformation in the crystal structure of Sirt2 (Fig. 2). These observations suggested that docked ligands have occupied the selective pocket in Sirt2 as inhabited by the co-crystallized SirReal2 inhibitor; hence, endorses the used docking protocol in the identification of potent FDA-approved drugs as selective putative inhibitors of Sirt2.

**Figure 3.**
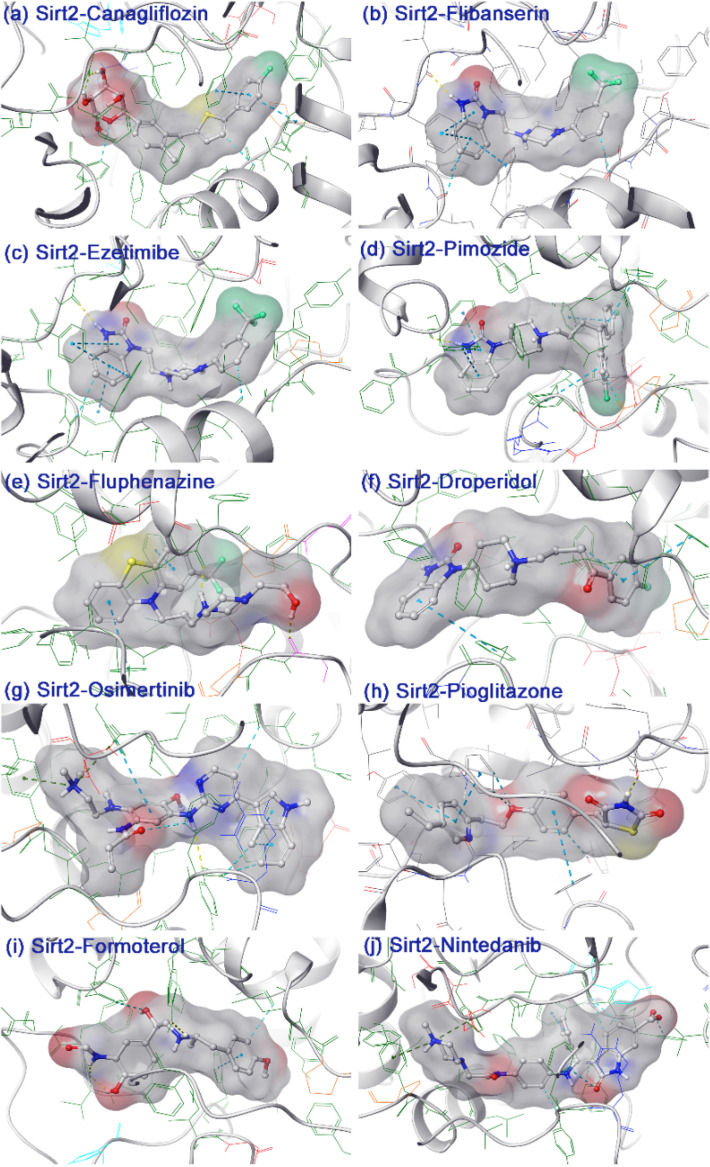
3D interaction map for the docked drugs, i.e., (**a**) Canagliflozin, (**b**) Flibanserin, (**c**) Ezetimibe, (**d**) Pimozide, (**e**) Fluphenazine, (**f**) Droperidol, (**g**) Osimertinib, (**h**) Pioglitazone, (**i**) Formoterol, and (**j**) Nintedanib, extracted within 4 Å space around the ligand in the selective pocket of Sirt2. Herein, a ligand surface was generated based on the charge of the atoms in the drug molecules. Academic Schrödinger-Maestro v12.4 suite^43^ has been utilized for rendering the images (URL: https://www.schrodinger.com/freemaestro).

Furthermore, each selected poses of FDA-approved drugs with Sirt2 were analyzed for docking energy and molecular contact formation around the docked ligand at 4 Å distance, including hydrogen bonding (H-bond), π–π/π–cation, hydrophobic, polar, negative, positive, and glycine interactions, against re-docked SirReal2 inhibitor in the selective pocket of Sirt2 (Fig. 3, Supplementary Figs. S3–S4). Among all the docked complexes, Sirt2-Canagliflozin complex revealed the highest −14.50 kcal/mol docking score via the formation of two hydrogen bonds (H-bond) at Val^233^ (O–H⋯O: 1.60 Å and O⋯H⋯O: 1.78 Å) residue and π–π interactions with Tyr^139^ and Phe^190^ residues, while Sirt2-Nintedanib complex showed the lowest docking energy of − 11.89 kcal/mol by a substantial contribution of π–π (Phe^234^ residue) and π-cations (Tyr^139^ and Phe^190^ residues) interactions (Table 1). Furthermore, docked conformation of Flibanserin (− 13.20 kcal/mol; H-bond: Val^233^ (N–H⋯O: 2.10 Å); π–π: Phe^119^, Phe^131^, and Phe^234^ (2)), Ezetimibe (− 12.78 kcal/mol; H-bond: Tyr^104^ (O–H⋯O: 1.97 Å) and Asp^170^ (O–H⋯O: 2.11 Å); π–π: Phe^96^, Phe^119^, Tyr^139^, and Phe^190^), Pimozide (− 12.73 kcal/mol; H-bond: Phe^119^ (N–H⋯O: 2.06 Å); π–π: Phe^96^, Phe^119^(2), Tyr^139^, Phe^190^, and Phe^234^), Fluphenazine (− 12.62 kcal/mol; H-bond: Leu^138^ (N–H⋯O: 1.79 Å); π–π: Phe^96^ and Phe^190^), Droperidol (− 12.18 kcal/mol; H-bond: Val^233^ (N–H⋯O: 2.12 Å); π–π: Phe^96^, Tyr^139^, and Phe^190^), Osimertinib (− 12.14 kcal/mol; H-bond: Tyr^104^ (N–H⋯O: 2.68 Å); π–π/*π–cation: Phe^119^(2), *Tyr^139^, and *Phe^190^), Pioglitazone (− 12.09 kcal/mol; H-bond: Val^233^ (N–H⋯O: 1.79 Å); π–π: Phe^96^, Tyr^139^, and Phe^190^), and Formoterol (− 12.02 kcal/mol; H-bond: Tyr^104^ (O–H⋯O: 2.63 Å); and Val^233^ (N–H⋯O: 1.86 Å and O–H⋯O: 2.26 Å); π–π: Phe^190^) drugs with Sirt2 were noted for considerable docking scores, which were noticeably contributed by hydrogen bond formations and π–π interactions with residues in selective pocket of Sirt2 (Table 1, Supplementary Fig. S3). Likewise, re-docking of SirReal2 inhibitor with Sirt2 exhibited − 11.68 kcal/mol docking score and formation of substantial π–π interactions with Phe^119^, Phe^131^, Phe^190^, and Phe^234^ residues (Table 1, Supplementary Fig. S4). Of note, all the docked complexes, including SirReal2 inhibitor, were noted for additional intermolecular contacts formation, i.e., hydrophobic, polar, negative, positive, and glycine interactions, indicated the contribution of intermolecular contacts in the stability of respective docked complexes (Table 1, Fig. 3, Supplementary Figs. S3–S4). Furthermore, screened drugs with Sirt2 displayed higher docking energy (> − 11 kcal/mol) compared to re-docked SirReal2 inhibitor and occupied the same interacting residues as observed in Sirt2-SirReal2 crystal complex (Table 1), demonstrated a high and common mode of selective Sirt2 inhibition by both selected drugs and SirReal2 inhibitor. Thus, molecular docking and molecular contacts profiling permit us to consider that selected FDA-approved drugs have the potential to inhibit Sirt2 protein through an action on a selective binding site to distort the active pocket as reported for SirReal2 inhibitor.

**Table 1:** Docking scores and molecular contact profiling for the docked FDA-approved drugs in the selective pocket of Sirt2.

S. no	Drugs	Docking score (kcal/mol)	H-Bond	π–π/*π–cation stacking	Hydrophobic	Polar	Negative	Positive	Glycine
1	Canagliflozin	− 14.50	Val^233^(2)	Tyr^139^, Phe^190^	Phe^96^, Tyr^104^, Phe^119^, Phe^131^, Leu^134^, Ala^135^, Leu^138^, Tyr^139^, Pro^140^, Phe^143^, Ile^169^, Phe^190^, Leu^206^, Ile^213^, Ile^232^, Val^233^, Phe^234^, Phe^235^	Thr^171^, His^187^	Asp^170^	Arg^97^	–
2	Flibanserin	− 13.20	Val^233^	Phe^119^, Phe^131^, Phe^234^(2)	Ile^93^, Phe^96^, Tyr^104^, Ile^118^, Phe^119^, Phe^131^, Leu^134^, Ala^135^, Leu^138^, Tyr^139^, Pro^140^, Phe^143^, Ile^169^, Phe^190^, Leu^206^, Ile^213^, Ile^232^, Val^233^, Phe^234^, Phe^235^	His^187^	Asp^170^	–	–
3	Ezetimibe	− 12.78	Tyr^104^, Asp^170^	Phe^96^, Phe^119^, Tyr^139^, Phe^190^	Ile^93^, Pro^94^, Phe^96^, Leu^103^, Tyr^104^, Ile^118^, Phe^119^, Phe^131^, Leu^134^, Ala^135^, Leu^138^, Tyr^139^, Pro^140^, Phe^143^, Ile^169^, Phe^190^, Leu^206^, Ile^213^, Ile^232^, Val^233^, Phe^234^, Phe^235^	Ser^88^, Asn^168^	Asp^95^, Asp^170^	–	–
4	Pimozide	− 12.73	Phe^119^	Phe^96^, Phe^119^ (2), Tyr^139^, Phe^190^, Phe^234^	Ile^93^, Pro^94^, Phe^96^, Leu^103^, Tyr^104^, Phe^119^, Phe^131^, Leu^134^, Ala^135^, Leu^138^, Tyr^139^, Pro^140^, Phe^143^, Ile^169^, Phe^190^, Leu^206^, Ile^213^, Ile^232^, Val^233^, Phe^234^, Phe^235^	Thr^171^	Asp^95^, Asp^170^	Arg^97^	–
5	Fluphenazine	− 12.62	Leu^138^	Phe^96^, Phe^190^	Ile^93^, Pro^94^, Phe^96^, Leu^103^, Tyr^104^, Phe^119^, Phe^131^, Leu^134^, Ala^135^, Leu^138^, Tyr^139^, Pro^140^, Phe^143^, Ile^169^, Phe^190^	–	Glu^137^, Asp^170^	–	Gly^92^, Gly^141^
6	Droperidol	− 12.18	Val^233^	Phe^96^, Tyr^139^, Phe^190^	Ile^93^, Phe^96^, Tyr^104^, Phe^119^, Phe^131^, Leu^134^, Ala^135^, Leu^138^, Tyr^139^, Pro^140^, Phe^143^, Ile^169^, Phe^190^, Leu^206^, Ile^213^, Ile^232^, Val^233^, Phe^234^, Phe^235^	Thr^171^	Asp^170^	Arg^97^	–
7	Osimertinib	− 12.143	Tyr^104^	Phe^119^(2), *Tyr^139^, *Phe^190^	Ile^93^, Pro^94^, Phe^96^, Leu^103^, Tyr^104^, Ile^118^, Phe^119^, Phe^131^, Leu^134^, Ala^135^, Leu^138^, Tyr^139^, Pro^140^, Phe^143^, Ile^169^, Phe^190^, Leu^206^, Ile^213^, Ile^232^, Val^233^, Phe^234^, Phe^235^		Glu^116^, Asp^170^	Arg^97^	–
8	Pioglitazone	− 12.09	Val^233^	Phe^96^, Tyr^139^, Phe^190^	Ile^93^, Phe^96^, Tyr^104^, Phe^119^, Phe^131^, Ala^135^, Leu^138^, Tyr^139^, Pro^140^, Phe^143^, Ile^169^, Phe^190^, Leu^206^, Ile^213^, Ile^232^, Val^233^, Phe^234^, Phe^235^	Thr^171^	Asp^170^	–	–
9	Formoterol	− 12.04	Tyr^104^, Val^233^(2)	Phe^190^	Phe^96^, Tyr^104^, Ile^118^, Phe^119^, Phe^131^, Leu^134^, Ala^135^, Leu^138^, Tyr^139^, Pro^140^, Phe^143^, Ile^169^, Phe^190^, Leu^206^, Ile^213^, Ile^232^, Val^233^, Phe^234^, Phe^235,^	His^187^	Asp^170^	–	–
10	Nintedanib	− 11.89	–	*Tyr^139^, *Phe^190^, Phe^234^	Phe^96^, Tyr^104^, Phe^119^, Phe^131^, Leu^134^, Ala^135^, Leu^138^, Tyr^139^, Pro^140^, Phe^143^, Ile^169^, Phe^190^, Leu^206^, Ile^213^, Ile^232^, Val^233^, Phe^234^, Phe^235^, Val^266^	Thr^171^, His^187^, Gln^267^	Asp^170^	Arg^97^	–
11	SirReal2 inhibitor	− 11.68	–	Phe^119^, Phe^131^, Phe^190^, Phe^234^	Ile^93^, Phe^96^, Tyr^104^, Ile^118^, Phe^119^, Phe^131^, Leu^134^, Ala^135^, Leu^138^, Tyr^139^, Pro^140^, Phe^143^, Ile^169^, Phe^190^, Ile^213^, Ile^232^, Val^233^, Phe^234^	His^187^, Thr^171^	Asp^170^	–	–

### Molecular mechanics/generalized born surface area analysis

**Figure 4:**
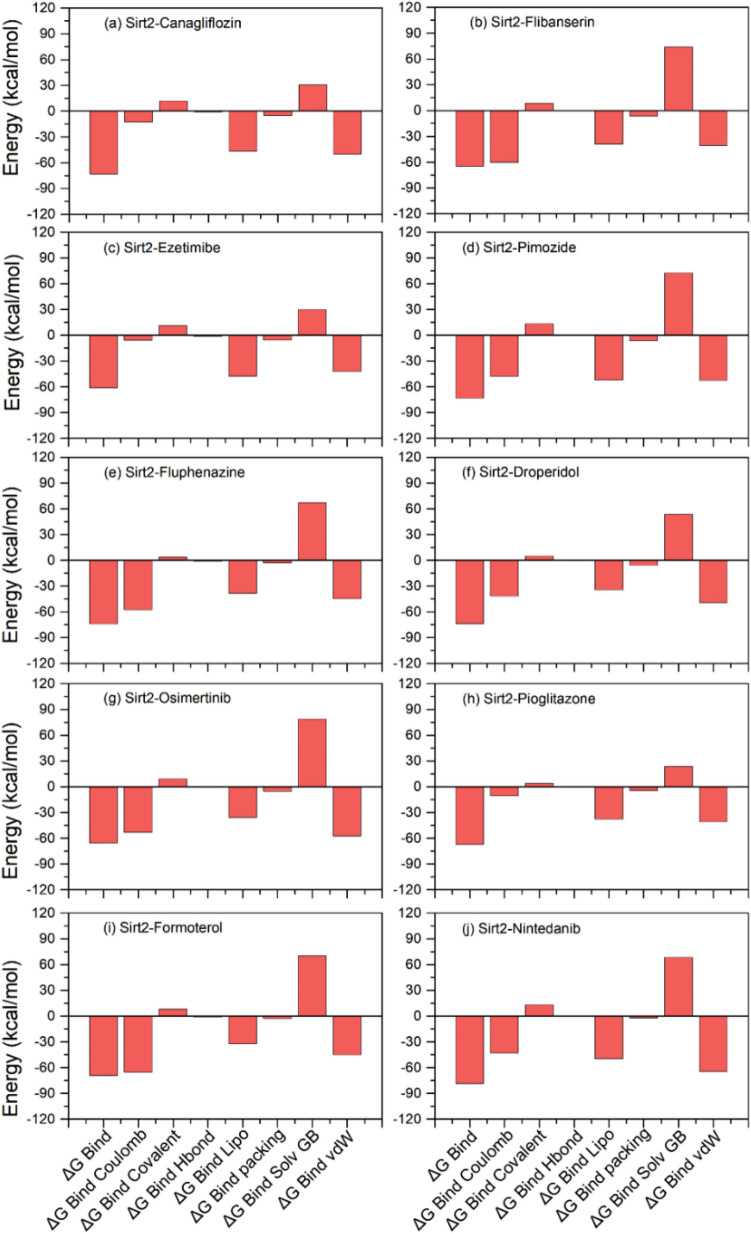
Estimation of binding free energy and individual dissociation energy components calculated for the docked complexes of FDA-approved drugs with Sirt2 using MM/GBSA method.

The molecular docking methods provide the spatial orientation of the docked ligand in the binding cavity of the receptor; hence, binding affinities have been suggested to consider for the docked complexes to avoid the pseudo positive hits collected from molecular docking analysis. Thereof, selected docked poses of the drugs with Sirt2 were analyzed for the binding free energy analysis by comparison to docked reference complex, i.e., Sirt2-SirReal2 inhibitor, via molecular mechanics/generalized Born surface area (MM/GBSA) method (Fig. 4, Supplementary Table S2).

Interestingly, docked Sirt2-drug complexes showed substantial binding affinity > − 60 kcal/mol, where Sirt2-Ezetimibe (− 61.33 kcal/mol) and Sirt2-Nintedanib (− 78.65 kcal/mol) complexes were noted for lowest and highest binding free energy values, respectively against Sirt2-SirReal2 inhibitor complex (− 96.22 kcal/mol) (Fig. 4, Supplementary Fig. S5). Furthermore, analysis of dissociation energy components demonstrated the significant contribution of ΔG_Bind Coulomb_, ΔG_Bind Lipo_, and ΔG_Bind vdW_ in the stability of docked complexes (Fig. 4, Supplementary Fig. S5). Based on these observations, selected docked poses of FDA-approved drugs with Sirt2 were concluded as potential candidates with an adequate binding affinity in the selective pocket of Sirt2 to act as a potent antagonist as SirReal2 inhibitor.

### Explicit solvent molecular dynamics simulation analysis

The molecular docked poses of potential drugs with Sirt2 were further considered for conformational stability, selective pocket occupancy, and intermolecular interactions profiling as a function of a 100 ns simulation interval via explicit solvent molecular dynamics (MD) simulation. The elucidation of docked conformation stability is crucially important to understand the inhibition of protein predicted by the molecular docking approach. In this context, MD simulation trajectory provides a conformational landscape of docked receptor-ligand complex at given conditions, such as temperature and pressure, with respect to simulation interval. Hence, MD simulation was performed on the selected docked conformations of Sirt2 with screened drugs for the 100 ns interval using Desmond v5.6 module of Schrödinger suite 2018–4.

#### Last pose and intermolecular interaction profiling

**Figure 5:**
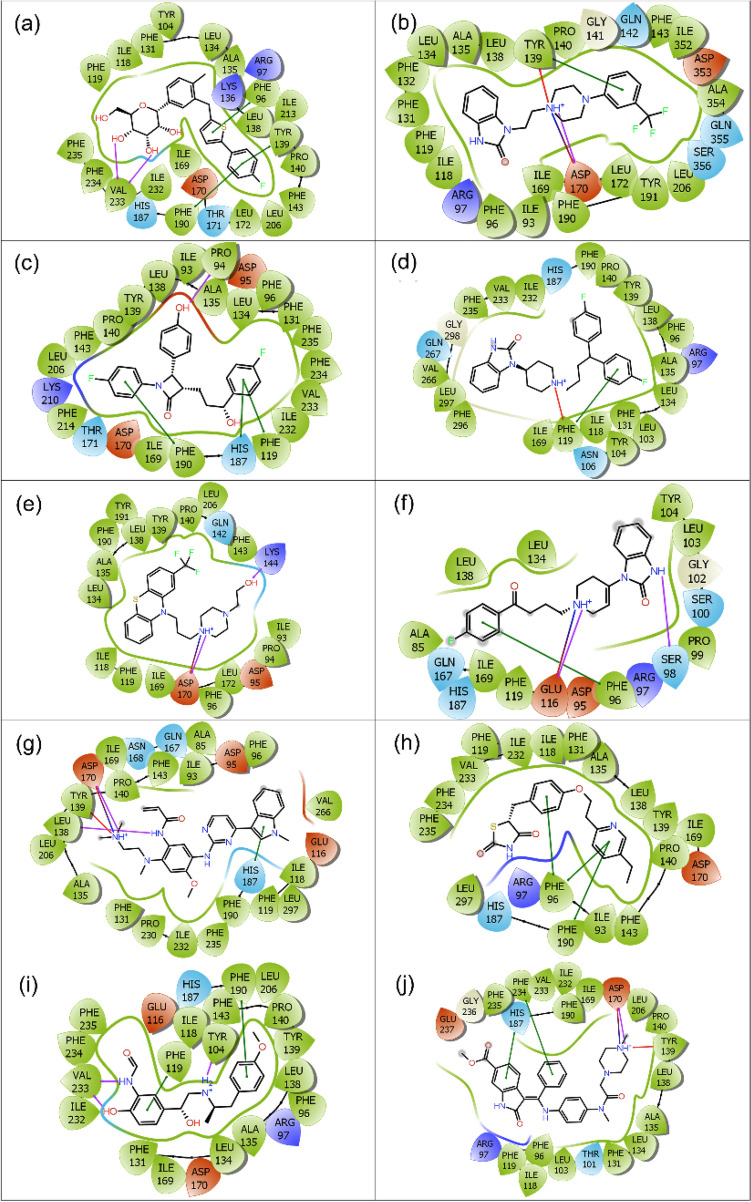
2D molecular contacts profiling for the last poses of Sirt2-FDA-approved drugs, viz. (**a**) Canagliflozin, (**b**) Flibanserin, (**c**) Ezetimibe, (**d**) Pimozide, (**e**) Fluphenazine, (**f**) Droperidol, (**g**) Osimertinib, (**h**) Pioglitazone, (**i**) Formoterol, and (**j**) Nintedanib, were extracted from 100 ns MD simulation. These poses exhibit hydrogen bond (pink arrows), π–π (green lines), π–cation (red lines), hydrophobic (green), polar (blue), negative (red), positive (violet), glycine (grey), and salt bridge (red-violet line) interactions in respective extracted snapshots. Images were rendered using academic Schrödinger-Maestro v12.4 suite^43^ (URL: https://www.schrodinger.com/freemaestro).

To determine the stability of docked drugs in the selective pocket of Sirt2, the last snapshots from each 100 ns MD trajectories were extracted and aligned to their respective docked poses in reference to ligand position (Supplementary Fig. S6–S7). Of note, all the selected drug poses showed < 10 Å RMSD, where highest and lowest RMSD values of 9.5465 and 1.5196 Å were noted for Sirt2-Droperidol and Sirt2-Nintedanib complexes, respectively by comparison to reference complex, viz. Sirt2-SirReal2 inhibitor (1.3246 Å). Moreover, extracted last snapshots from each MD trajectories were also studied for the formation of intermolecular contacts between the ligand and residues in the selective pocket of Sirt2 (Supplementary Table S3, Fig. 5, Supplementary Fig. S8). Herein, all the docked Sirt2 complexes with selected ligands, except Pimozide, Pioglitazone, and SirReal2 inhibitor, showed one or more than one hydrogen bond formation at Pro^94^, Ser^98^, Tyr^104^, Glu^116^, Leu^138^, Lys^144^, Asp^170^, and Val^233^ residues. Besides, π–π, π–cations, hydrophobic, polar, negative, positive, glycine, and salt bridge interactions were also noted in the extracted respective snapshots by comparison to Sirt2-SirReal2 inhibitor complex (Supplementary Table S3, Fig. 5, Supplementary Fig. S8). Interestingly, these molecular contact formations with active residues of Sirt2 protein were also noted in the respective initial docked poses of Sirt2 with selected drugs and SirReal2 inhibitor (Table 1). Conclusively, analysis of last pose conformation and molecular contacts profiling suggested the considerable occupancy and stability of selected drugs in the selective pocket of Sirt2 in reference to SirReal2 inhibitor.

To further obtained the insights into the stability of docked Sirt2-drugs complexes during MD simulation, each MD trajectories were computed for statistical analysis in terms of (a) root-mean-square-deviation (RMSD), (b) root-mean-square fluctuation (RMSF), and (c) protein–ligand contacts mapping by comparison to reference complex, i.e., Sirt2-SirReal2 inhibitor, as a function of 100 ns simulation interval.

#### RMSD and RMSF analysis

**Figure 6:**
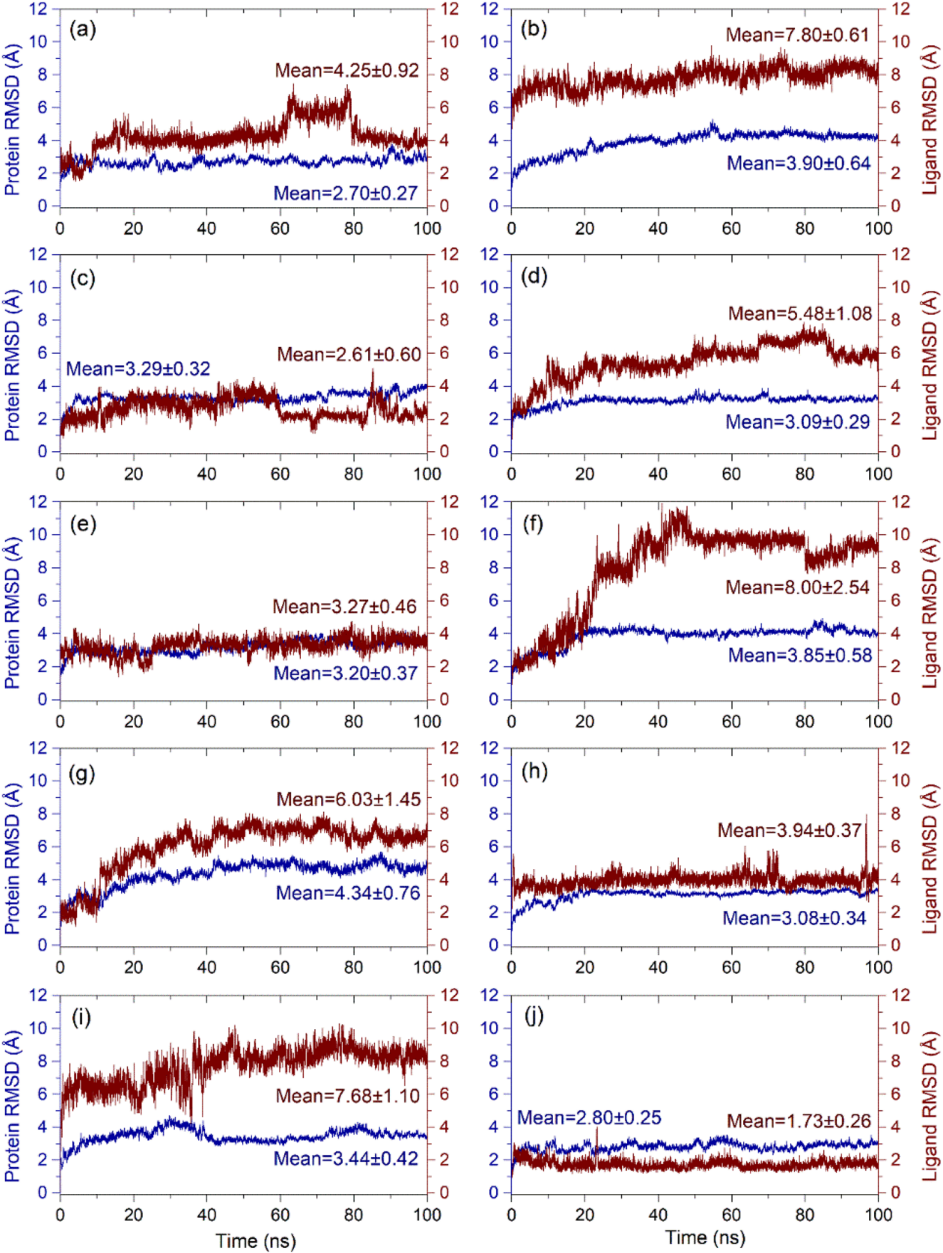
Calculated RMSD values for alpha carbon (Cα) atoms (blue curves) of Sirt2 protein and protein fit ligands (red curves), viz. (**a**) Canagliflozin, (**b**) Flibanserin, (**c**) Ezetimibe, (**d**) Pimozide, (**e**) Fluphenazine, (**f**) Droperidol, (**g**) Osimertinib, (**h**) Pioglitazone, (**i**) Formoterol, and (**j**) Nintedanib, were plotted with respect to 100 ns simulation interval.

Analysis of MD simulations in terms of root-mean-square deviations (RMSD) of average simulated poses computed from all the frames generated during simulation in reference to the initial structure as a function of time trajectory can be used to examine the convergence of simulated protein–ligand complex. Thereof, initially, RMSD values for protein (Cα) and protein fit ligands, viz. FDA-approved drugs and SirReal2 inhibitor, were extracted from each 100 ns simulation trajectories with respect to initial pose by comparison to free Sirt2 structure (Fig. 6, Supplementary Figs. S9–S10). The Cα atoms in Sirt2 complexed with screened drugs showed mean deviations (< 4 Å), except for Sirt2-Osimertinib complex (4.34 ± 0.74 Å) against free Sirt2 protein (3.15 ± 0.54 Å) during 100 ns simulation interval (Fig. 6, Supplementary Fig. S9). Meanwhile, Cα atoms of Sirt2 docked with SirReal2 inhibitor revealed a stable RMSD < 2.2 Å till 70 ns followed by considerable elevation and state of equilibrium with a mean 2.64 ± 0.40 Å RMSD at the end of MD simulation (Supplementary Fig. S10). Likewise, RMSD analysis of protein fit ligands exhibited acceptable deviations (< 5 Å) during the first 40 ns followed by a state of equilibrium, except for Flibanserin, Pimozide, Droperidol, Osimertinib, and Formoterol drugs, was noted with > 4 Å RMSD values at end of 100 ns MD simulation (Fig. 6). Besides, simulation trajectory of reference complex, i.e., Sirt2-SirReal2 inhibitor, also demonstrated considerable stability and steadiness in SirReal2 inhibitor with mean 2.95 ± 0.31 Å RMSD during MD simulation (Supplementary Fig. S10). It is considerable to mentioned that among the screened drugs, only Ezetimibe (2.61 ± 0.60 Å), Fluphenazine (3.27 ± 0.46 Å), Pioglitazone (3.94 ± 0.37 Å), and Nintedanib (1.73 ± 0.26 Å), displayed the most acceptable deviations and equilibrium throughout the simulation process by comparison to SirReal2 inhibitor (2.95 ± 0.31 Å) (Fig. 6, Supplementary Fig. S10). Hence, these drugs, viz. Ezetimibe, Fluphenazine, Pioglitazone, and Nintedanib, were marked for substantial stability in the selective pocket of Sirt2 against other selected FDA-approved drugs and SirReal2 inhibitor.

Furthermore, the flexibility of Sirt2 and screened drugs in respective complexes against free Sirt2 protein and Sirt2-SirReal2 inhibitor complex was monitored via RMSF analysis of respective 100 ns MD simulation trajectories (Supplementary Figs. S11–13). Notably, residues of the receptor in all the docked complexes with screened drugs demonstrated a similar RMSF (< 2.0 Å) pattern by comparison to free Sirt2 protein, except relatively significant changes in RMSF (~ 4.0–6.5 Å) values were noted in the docked Sirt2 with Ezetimibe, Fluphenazine, Pioglitazone, and Nintedanib drugs against the free protein (~ 5.0–6.5 Å) at a region noted for the interaction of ligands, loop region, and C-terminal of Sirt2 protein during simulation interval (Supplementary Figs. S11–12). Similarly, a variation in RMSF (~ 3.0–6.5 Å) value was also monitored for interacting residues of Sirt2 with SirReal2 inhibitor during MD simulation (Supplementary Fig. S13). Additionally, RMSF computed for the atoms of docked ligands showed an acceptable fluctuation value with an exception in atoms intermingling with residues in the selective pocket of Sirt2 by comparison to SirReal2 inhibitor, which indicates the substantial complex stability during simulation interval (Supplementary Figs. S12–S13). Together, these results demonstrated the stability of docked drugs against SirReal2 inhibitor in the selective pocket of Sirt2 during 100 ns MD simulation.

#### Protein–ligand interaction mapping

**Figure 7:**
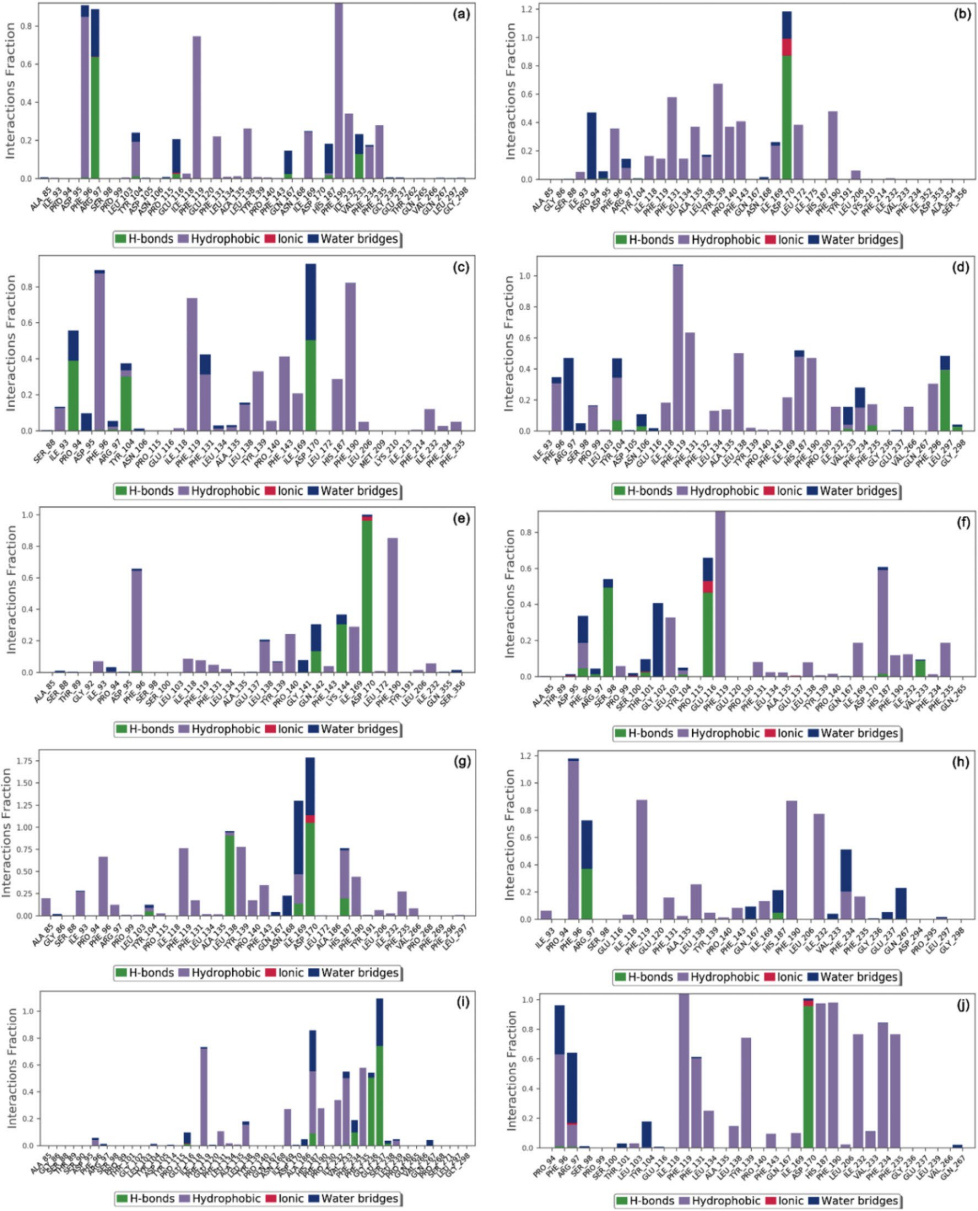
Protein–ligand interaction mapping for Sirt2 docked with selected FDA-approved drugs, i.e., (**a**) Canagliflozin, (**b**) Flibanserin, (**c**) Ezetimibe, (**d**) Pimozide, (**e**) Fluphenazine, (**f**) Droperidol, (**g**) Osimertinib, (**h**) Pioglitazone, (**i**) Formoterol, and (**j**) Nintedanib, extracted from respective 100 ns MD trajectories.

The stability of docked Sirt2-drugs complexes was further evaluated in terms of intermolecular interaction formation, including hydrogen bonding, hydrophobic interactions, ionic interactions, and water bridge formation, during the simulation interval against docked Sirt2-SirReal2 inhibitor complex (Fig. 7, Supplementary Fig. S14). Remarkably, all the docked drugs showed substantial interactions with the residues in the selective pocket of Sirt2 structure during 100 ns simulation interval (Fig. 7); these residues were also noted for molecular contact formations in the respective docked complexes (Table 1).

Of note, extracted protein–ligand maps for Sirt2 with screened drugs exhibited significant hydrogen and hydrophobic interactions in reference to Sirt2-SirReal2 inhibitor, which displayed only hydrophobic and water bridge formation (Supplementary Fig. S14). Furthermore, screened drugs were noted for hydrogen bond and hydrophobic interactions with the essential residues in the selective pocket of Sirt2 at 30% of the total simulation interval (Supplementary Fig. S15). However, Sirt2-SirReal2 inhibitor complex exhibited only hydrophobic and π–π interactions at 30% of the total 100 ns MD simulation (Supplementary Fig. S16). It is important to mention that screened drugs and SirReal2 inhibitor shared common residues in intermolecular interaction maps extracted from respective 100 ns MD simulation trajectories, which were also noticed in the respective docked complexes (Table 1). Conclusively, these results indicated the similar binding pose of screened drugs with substantial stability in the selective pocket of Sirt2 against SirReal2 inhibitor.

### Post-molecular dynamics simulation analysis

#### Essential dynamics analysis

Essential dynamics in terms of principal component analysis (PCA), a covariance matrix mathematical technique, was used to determine the domain dynamics and atomic distance in Sirt2 structure docked with screened drugs against SirReal2 inhibitor and free Sirt2 protein from respective 100 ns simulation trajectories. Initially, the proportion of variance (%) as Eigen fraction for the mean square positional variations of the covariance matrix was extracted as a function of 20 eigenmodes (Supplementary Figs. S17–S19). Interestingly, all the extracted Sirt2 conformations showed a steep drop in the percentage of variance corresponds to the initial three eigenmodes and covered more than > 60% of the total proportion of variance in each simulated Sirt2-drug complexes, except Pimozide, Fluphenazine, Droperidol, and Pioglitazone were noted for < 60% variance by comparison to Sirt2 docked with SirReal2 inhibitor (53.4% variance) (Supplementary Figs. S17–S18). A similar steep down in proportion of variance with compact values was also recorded against eigenvalue in the free Sirt2 structure (67.7% variance) (Supplementary Fig. S19). These results indicated that docked drugs have induced significant variations in the conformational fluctuations against SirReal2 inhibitor in the selective pocket of Sirt2 by comparison to the free Sirt2 structure. Following six eigenmodes, each system depicted an elbow point and non-significant changes in the Eigen fraction till 20 eigenvalues (Supplementary Figs. S17–S19). Together, these interpretations indicated the stability of respective complexes and marked for the relatively higher induced fluctuations in Sirt2 structure against SirReal2 inhibitor that disturbed the functional motions in Sirt2 with respect to time.

**Figure 8:**
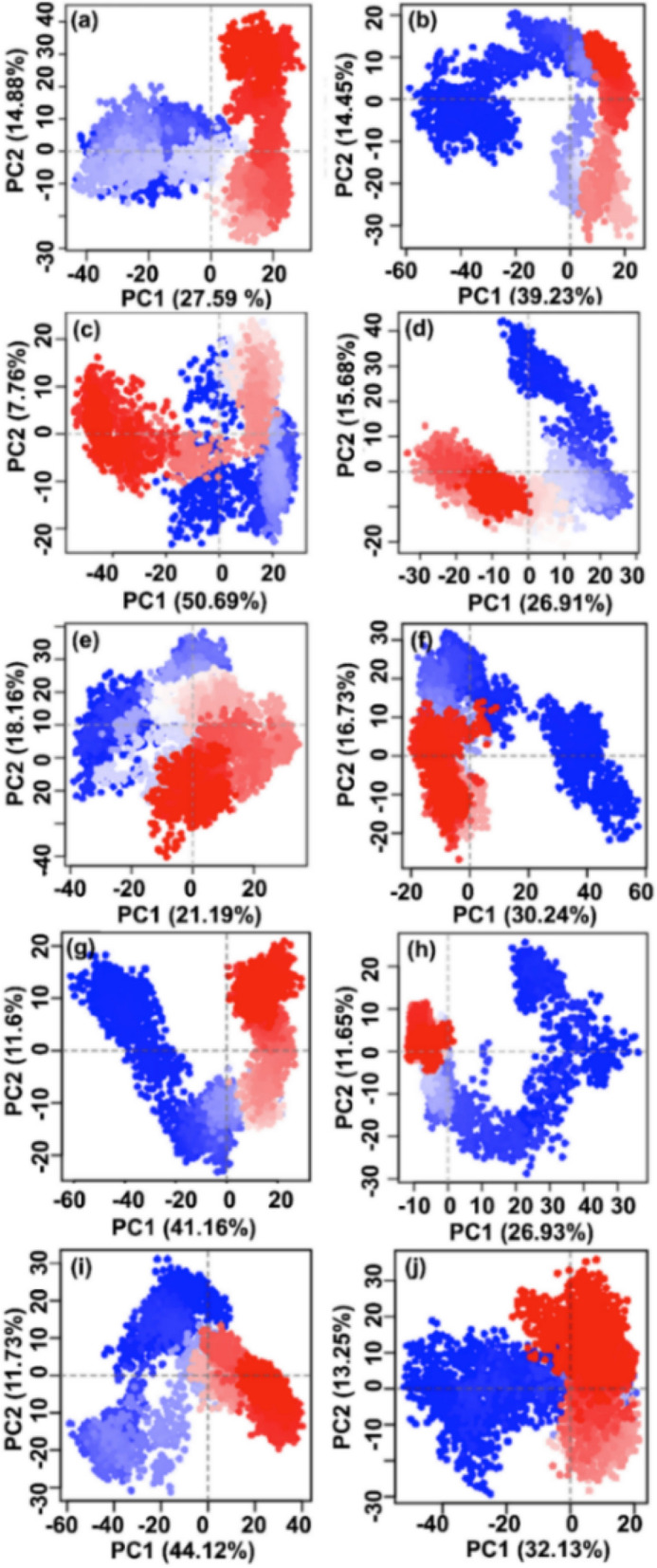
Score plots for the computed principal components (PC1 vs PC2) from 100 ns MD simulation trajectories of Sirt2 docked with selected drugs, i.e. (**a**) Canagliflozin, (**b**) Flibanserin, (**c**) Ezetimibe, (**d**) Pimozide, (**e**) Fluphenazine, (**f**) Droperidol, (**g**) Osimertinib, (**h**) Pioglitazone, (**i**) Formoterol, and (**j**) Nintedanib. The incessant color scale from blue to white to red directs the periodic jumps between the structural poses of Sirt2 as a function of the 100 ns simulation interval. Images has been rendered in Bio3d package (Released version 2.4-1; URL: http://thegrantlab.org/bio3d/)^52^ under R environment (R version 4.0.4; URL: http://mirror.fcaglp.unlp.edu.ar/CRAN/)^53^.

Furthermore, the first three eigenvectors or principal components (PCs) were extracted and plotted for Sirt2 structure docked with screened drugs and SirReal2 inhibitor as cluster groups from the respective MD simulation trajectories. Remarkably, all three extracted PCs indicated substantial dynamic motions between − 60 and 60 coordinates by comparison to the first PCs extracted for Sirt2 protein docked with SirReal2 inhibitor (− 30 and 30 coordinates) and free Sirt2 structure (− 30 and 30 coordinates) as shown in Fig. 8, Supplementary Figs. S18–S21. Also, the 2D score plots for extracted PCs demonstrate the variation in the clusters during the 100 ns MD simulation interval via gradient change in color from blue to red, which represents the substantial periodic bounces between the various conformational poses of the protein in docked states with drugs against SirReal2 inhibitor and free Sirt2 structure. Convincingly, dynamic motions of clusters in each extracted PCs for the respective protein structure docked with screened drugs suggested the induction of collective fluctuation by docked drugs in Sirt2 protein against SirReal2 inhibitor and free Sirt2 structure as a function of 100 ns MD simulation interval. Hence, screened drugs were concluded with significant distortion in Sirt2 structure by comparison to SirReal2 inhibitor and supported as putative inhibitors to induced rearrangement in the selective pocket of Sirt2.

#### End-point binding free energy calculation

**Figure 9:**
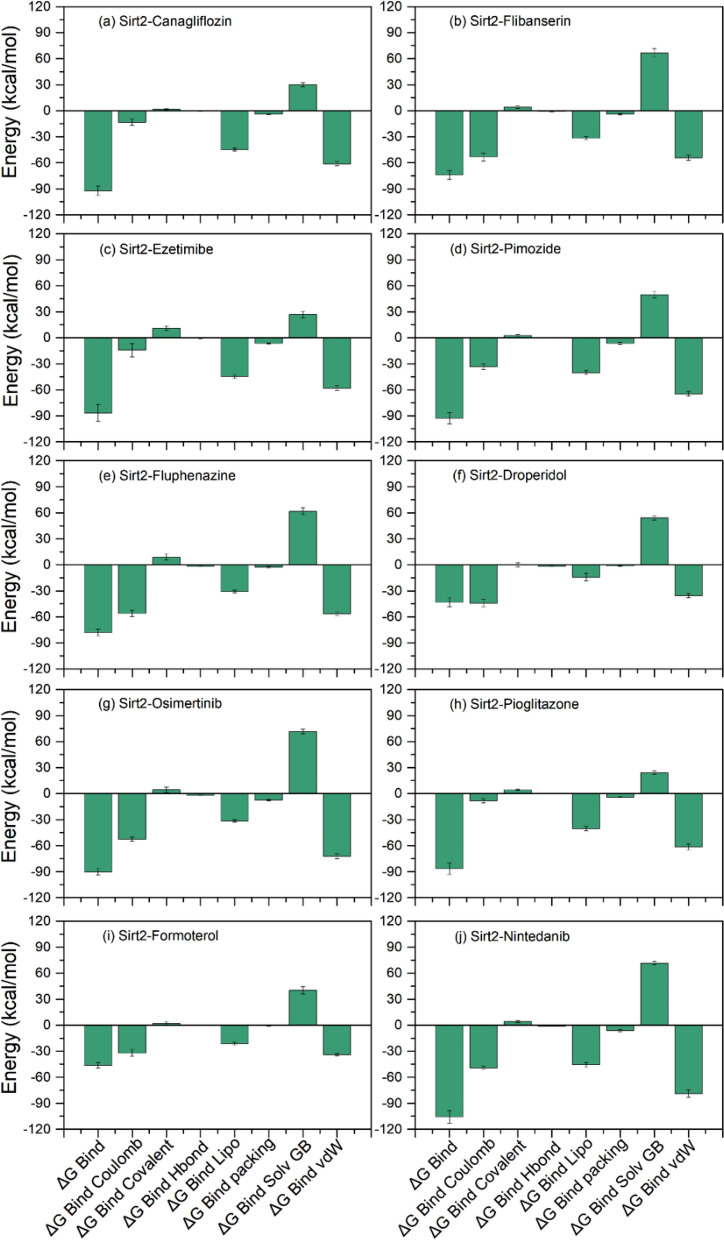
End-point binding free energy (kcal/mol) and dissociation energy components values computed for extracted snapshots of Sirt2 with screened FDA-approved drugs, i.e., (**a**) Canagliflozin, (**b**) Flibanserin, (**c**) Ezetimibe, (**d**) Pimozide, (**e**) Fluphenazine, (**f**) Droperidol, (**g**) Osimertinib, (**h**) Pioglitazone, (**i**) Formoterol, and (**j**) Nintedanib, from respective 100 ns MD simulation trajectories.

Molecular mechanics generalized Born surface area (MM/GBSA) based end-point binding free energy method by considering thermodynamics and desolvation parameters was applied on the extracted protein–ligand complexes from the last 10 ns of 100 ns MD simulation (Supplementary Table S4, Fig. 9, Supplementary Fig. S22). The end-point binding free energy and energy dissociation components, i.e., ΔG_Bind Coulomb_, ΔG_Bind Covalent_, ΔG_Bind Hbond_, ΔG_Bind Lipo_, ΔG_Bind Packing_, ΔG_Bind Solv GB_, and ΔG_Bind vdW_, calculated for each simulated Sirt2 docked complexes are given in Supplementary Table S4. Remarkably, all the complexes of screened drugs exhibited considerable binding free energy (> − 70 kcal/mol), except for Sirt2-Formoterol complex (− 46.2 ± 3.17 kcal/mol) and Sirt2-Droperidol complex (− 43.32 ± 4.98 kcal/mol). Moreover, the complexes of Sirt2 with Canagliflozin (− 92.37 ± 5.22 kcal/mol), Pimozide (− 92.96 ± 6.54 kcal/mol), Osimertinib (− 90.3 ± 3.62 kcal/mol), and Nintedanib (− 105.58 ± 7.28 kcal/mol) drugs showed considerable binding free energy against Sirt2-SirReal2 inhibitor (− 99.82 ± 5.21), suggested the substantial interaction and occupancy of potent drugs in the selective pocket of Sirt2 (Fig. 9, Supplementary Fig. S22). Furthermore, calculated dissociation energy components for each complex suggested the significant contribution of ΔG_Bind Coulomb,_ ΔG_Bind Lipo,_ and ΔG_Bind vdW_ to the net binding free energy of the complexes while ΔG_Bind Covalent_ and ΔG_Bind Solv GB_ were detected for unfavorable energy contribution to the ligand-binding with protein in respective complexes.

#### Quantum mechanics/molecular mechanics binding energy calculation

The last snaps from each 100 ns MD trajectories were studied for hybrid QM/MM binding energy to further establish the affinity and stability of the screened drugs against SirReal2 inhibitor in the selective pocket of Sirt2 protein. The cumulative energy calculations, including, docking score, binding free energy before and MD simulation, and hybrid QM/MM binding energy values for screened complexes are given in Table 2. It can be observed that all the screened drugs, except Canagliflozin, Flibanserin, Droperidol, and Formoterol, docked with Sirt2 exhibited superior QM/MM binding energy by comparison to SirReal2 inhibitor. Of note, substantial QM/MM binding energy of − 329.01 and − 386.04 kcal/mol were noted for Sirt2-Fluphenazine and Sirt2-Nintedanib, respectively against other drugs (< − 107 kcal/mol) and Sirt2-SirReal2 inhibitor (− 69.74 kcal/mol). It is important to mention that although an increase in negative binding free energy after MD simulation directed to the stability of the docked complexes, computed hybrid QM/MM binding energy values were not consistent with docking and binding free energy scores calculated for the respective docked complexes. However, based on the QM/MM binding energy, Fluphenazine and Nintedanib drugs exhibit substantially better interactions and stability in the selective pocket of Sirt2 protein against SirReal2 inhibitor.

**Table 2:** Cumulative energy data for the screened drugs and reference inhibitor in the selective pocket of Sirt2 protein.

S.no	Complexes	Energy (kcal/mol)			
		Docking score	MM/GBSA score (Docked complex)	MM/GBSA score (MD trajectory)	QM/MM binding energy
1	Sirt2-Canagliflozin	− 14.499	− 73.26	− 92.37 ± 5.22	− 62.14
2	Sirt2-Flibanserin	− 13.203	− 64.75	− 74.10 ± 5.11	− 37.47
3	Sirt2-Ezetimibe	− 12.784	− 61.33	− 86.86 ± 9.68	− 83.06
4	Sirt2-Pimozide	− 12.731	− 73.31	− 92.96 ± 6.54	− 107.41
5	Sirt2-Fluphenazine	− 12.619	− 73.97	− 77.99 ± 3.83	− 329.01
6	Sirt2-Droperidol	− 12.177	− 73.73	− 43.33 ± 4.98	− 43.37
7	Sirt2-Osimertinib	− 12.143	− 65.54	− 90.30 ± 3.62	− 71.89
8	Sirt2-Pioglitazone	− 12.091	− 67.18	− 86.74 ± 6.56	− 86.10
9	Sirt2-Formoterol	− 12.037	− 69.14	− 46.21 ± 3.17	− 55.61
10	Sirt2-Nintedanib	− 11.886	− 78.65	− 105.58 ± 7.28	− 386.04
11	Sirt2-SirReal2 inhibitor	− 11.684	− 96.22	− 99.82 ± 5.21	− 69.74

### 3D-QSAR models analysis

In this study, Atom-Based 3D-QSAR and Field-Based 3D-QSAR models were developed using the 39 known selective inhibitors, including SirReal2 inhibitors, of Sirt2 protein with biological activity (IC50 = 0.02–57 µM) to predict the activity of selected FDA-approved drugs as putative inhibitors of Sirt2. Supplementary Figure S23 shows the 2D structures and names for the selective inhibitors of Sirt2 extracted from the respective references. Since the collected known inhibitors were validated by in vitro inhibition assay, these inhibitors were initially studied for the binding affinity at the selective pocket of Sirt2 protein using a stringent XP docking approach. Interestingly, all the collected known inhibitors showed substantial docking scores (− 12.297 to − 6.693 kcal/mol) (Supplementary Table S5) and interactions with the residues in the selective pocket of Sirt2 (Supplementary Fig. S24). Notably, docking scores for the known selective inhibitors were comparatively less than the selected FDA-approved drugs and interacting residues were the same as noted in the docked Sirt2-FDA-approved drug complexes and Sirt2-SirReal2 inhibitor complexes (Table 1, Supplementary Fig. S24). Based on the docking and intermolecular interaction analysis, it was concluded that 39 known selective inhibitors of Sirt2, including SirReal2 inhibitor, can be considered for the 3D-QSAR model generation to predict the activity of selected FDA-approved drugs. Following, Atom-Based 3D-QSAR (Supplementary Tables S6–S7) and Field-Based 3D-QSAR (Supplementary Table S8–S9) models were developed and validated to elucidate the functional activity of the selected FDA-approved drugs using Partial Least Squares (PLS) regression analysis. In both, 3D-QSAR models, 70% of the dataset ligands were randomly segregated to form the training set while the rest of the 30% ligands were treated as the test set for internal validation. Following stringent analysis of the developed models, PLS-5 for both the types of 3D-QSAR models was considered to be the best model with considerable regression coefficients R^2^, R^2^ CV, RSME, Q^2^, and Pearson-r coefficient values (Supplementary Tables S6–S9), and hence, the developed models were considered for further prediction of screened FDA-approved drugs. For the visual depiction of the developed 3D-QSAR models, ligands with two sets of compounds, i.e., with least activity (JFD00244, Cambinol, Sirtinol and 1(A21) and high activity (Compound 69, Compound 67, Compound 66, and Compound 29), were superimposed to render the respective 3D descriptors calculated from the models.

**Figure 10:**
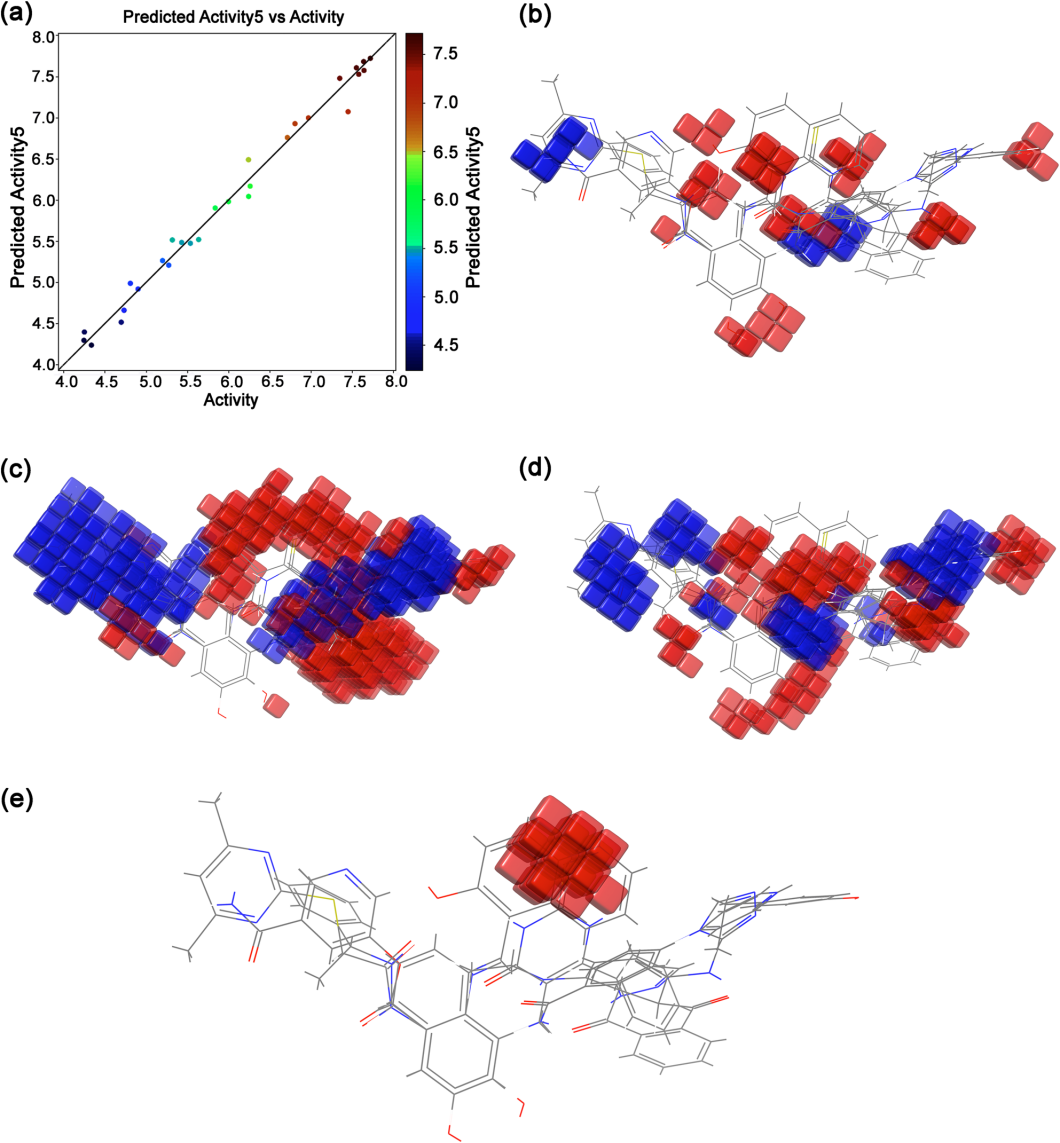
Atom Based 3D-QSAR model development for the known selective inhibitors of Sirt2; (**a**) scatter plot exhibiting experimental versus predicted activities of the training set, (**b**) hydrogen donor atoms map, (**c**) hydrophobic atoms map, (**d**) electron-withdrawn atom map, and (**e**) other contributing factors map. Images were rendered using academic Schrödinger-Maestro v12.0 suite^35^ (URL: https://www.schrodinger.com).

Atom-Based 3D-QSAR renders the information regarding hydrogen bond donor, hydrophobic, electron-withdrawing, positive ionic, and negative ionic features where atoms were considered. Statistical analysis indicated the PLS-5 with a higher regression coefficient (R^2^) value of 0.987 cross-validated correlation coefficient (R^2^ CV) of 0.8047, RMSE of 0.92, Q^2^ of 0.2846, and Pearson-r of 0.5504 for the training set. The actual and predicted pIC50 values of the dataset ligands for atom-based 3D-QSAR are shown in Supplementary Table S8 and the regression scatter plot for the training set is shown in Fig. 10. Moreover, the Atom-Based 3D-QSAR model displays the 3D features as cubes or occlusions that mark the model and color corresponding to the sign of their coefficient values, where blue color indicates the positive coefficients and red color links with negative coefficients. Thus, a positive coefficient represents an increase, and a negative coefficient stands for a decrease in the activity. These features assist in the identification of functional groups or substituents that are required or undesirable at a certain position in a molecule. It can be monitored that the region linked with hydrophobic atoms sites and electron-withdrawn atom regions indicates higher blue occlusion against hydrogen donor atoms regions and other factors contributing sites (Fig. 10), suggested the favorable regions for activity elucidated by developed 3D-QSAR model.

**Figure 11:**
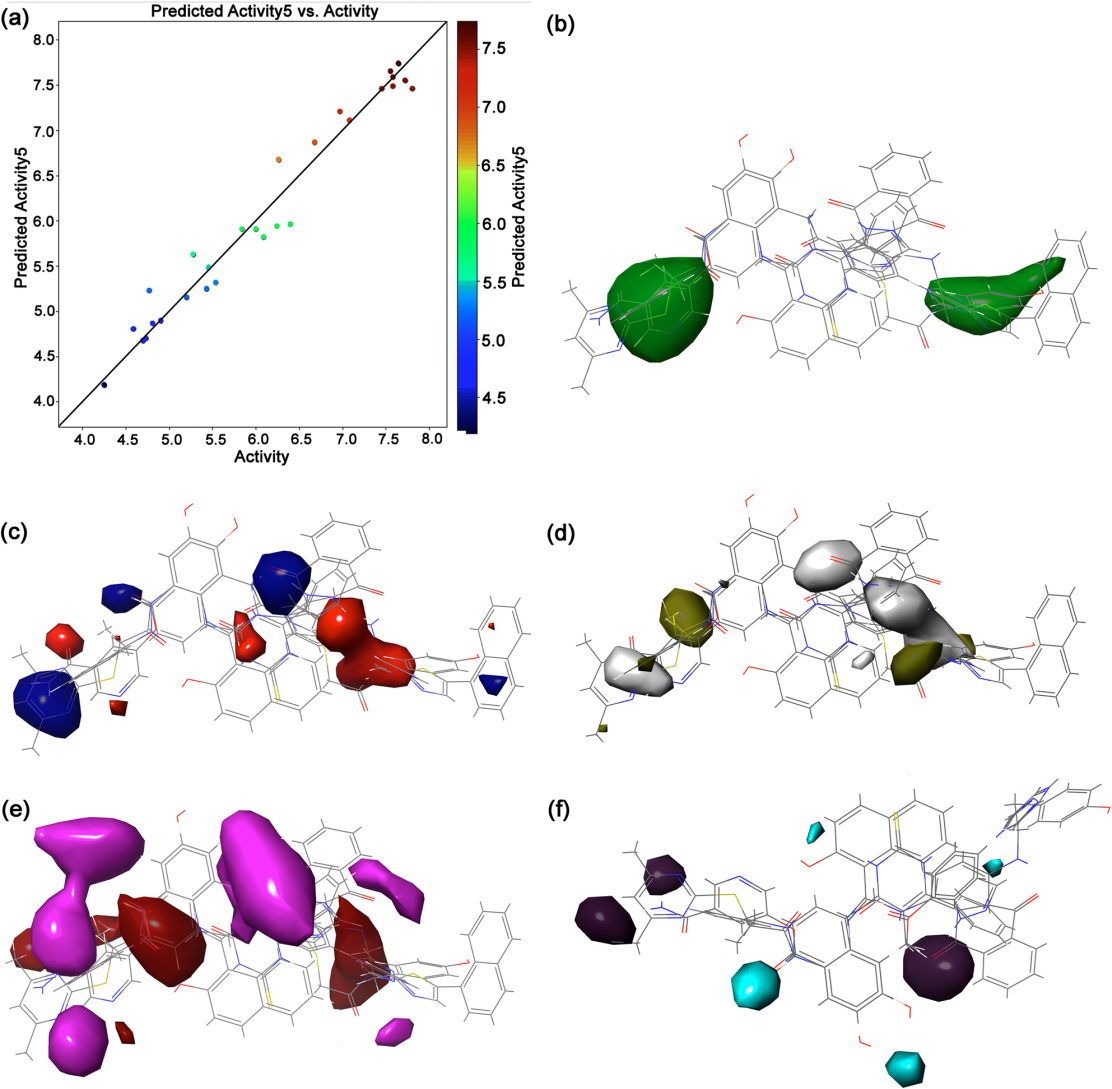
Field Based 3D-QSAR model development for the known selective inhibitors of Sirt2; (**a**) scatter plot exhibiting experimental versus predicted activities of the training set, (**b**) Gaussian sterric (**c**) Gaussian electrostatic, (**d**) Gaussian hydrophobic, (**e**) Gaussian hydrogen acceptor, (**f**) Gaussian hydrogen donor, counter surface maps plotted on the aligned ligands. Academic Schrödinger-Maestro v12.0 suite^35^ was used to renders the images (URL: https://www.schrodinger.com).

Furthermore, the Field-Based 3D-QSAR model considers the pIC50 as a dependent variable, whereas, steric, electrostatic, hydrophobic, hydrogen bond donor, and hydrogen bond acceptor potential fields are treated as independent variables. Of note, Gaussian fractions, such as steric, hydrophobic, and H-bond acceptor, are known to contribute as the major constituents for the biological activity of the compounds. In this model, actual and predicted pIC50 values of the dataset ligands along with statistical analysis are given in Supplementary Table S8–S9 while regression scatter plot for the training set is provided in Fig. 11. Herein, regression analysis concluded the PLS-5 model with a higher regression coefficient (R^2^) value of 0.9639, cross-validated correlation coefficient (R^2^ CV) of 0.596, RMSE of 0.92, Q^2^ of 0.3205, and Pearson-r of 0.8744 for the training set (Supplementary Table S9). Moreover, the green contour for Gaussian steric groups indicated the favorable regions for the substituents attached to the aligned ligands while electrostatic contours associated with Gaussian electrostatic fields in blue and red color region around the substituents of the aligned ligands depicts the favor and disfavors the activity, respectively (Fig. 11). Moreover, the yellow and white contour for the hydrophobic groups indicated favor and disfavor to the activity in the developed model while hydrogen bond acceptors magenta and red-colored contour distribution were also noted to disfavor and favor the activity, respectively (Fig. 11). Likewise, contours for hydrogen bond donor shown violet-colored region around the multi aligned ligands exhibits favorable regions for activity along with gray region associated with disfavor activity in the 3D-QSAR model (Fig. 11).

Moreover, the validated models of Atom-Based 3D-QSAR and Field-Based 3D-QSAR were used to predict the activity of selected FDA-approved drugs (Table 3). Among all the screened drugs, Nintedanib shows higher activity of pIC50 = 6.051 µM and pIC50 = 5.94 µM in Atom-Based 3D-QSAR and Field-Based 3D-QSAR models, respectively. Of note, Nintedanib was also observed with substantial stability in the selective pocket of Sirt2 and showed higher binding energy before and after MD simulation (Table 2). Based on these results, selected FDA-approved drugs were advocated for superior biological activity as putative selective inhibitors of Sirt2 protein and suggested to be considered for further experimental analysis in the development of potent drugs against Sirt2 protein-associated diseases and disorders.

**Table 3:** Predicted activity values for the selected FDA-approved drugs using developed and validated 3D-QSAR models.

S.no	Drugs	Atom-based 3D-QSAR	Field-based 3D-QSAR
		Predicted Activity5 (pIC50)	Predicted Activity5 (pIC50)
1	Canagliflozin	5.447	4.796
2	Flibanserin	5.245	4.540
3	Ezetimibe	4.951	5.707
4	Pimozide	5.540	4.534
5	Fluphenazine	5.214	4.824
6	Droperidol	5.488	4.523
7	Osimertinib	5.451	4.840
8	Pioglitazone	4.860	5.575
9	Formoterol	5.105	4.912
10	Nintedanib	6.051	5.940

## Discussion

Several studies have been reported for the human isotype Sirt2 in the modulation of cancer pathogenesis, inflammation, metabolic diseases, and neurodegeneration, projected the regulation of Sirt2 activity as a promising approach for pharmaceutical intrusions^66^. In this context, functions of Sirt2 in the cellular environment to some extend have been elucidated and several crystal structures in complex with selective and/or potent inhibitors were also resolved^30–32^. However, these reported inhibitors lack either specificity, potency, or drug-likeness^23^; hence, result in constant demand for Sirt2 inhibitors with selective and drug-like physicochemical properties.

The discovery of a selective SirReal2 inhibitor, which inhibited Sirt2 protein by induced rearrangement of the active site, has revealed a yet-unexploited binding pocket for the screening and development of novel Sirt2 inhibitors^23^. Hence, we have screened 2102 FDA-approved drugs in the selective pocket of Sirt2 to identify the lead drugs which can inhibit Sirt2 by binding in the same selective pocket of SirReal2 inhibitor using the computational modeling and dynamics simulations. Initially, screening of FDA-approved drugs in the selective pocket of Sirt2 yields 48 drugs from the collection of 2102 compounds by stringent XP docking protocol of GLIDE tool in Schrödinger suite. This method was used to reduce the possibility of pseudo positive hits via consideration of polar interactions, Coulombic, hydrogen bond, hydrophobic contacts, van der Waals, metal binding, freezing rotatable bonds, water desolvation energy, binding affinity enriching interactions, and the penalty for buried polar groups in XP scoring approach^41^. Following, docked conformations of top 10 drug candidates with least docking RMSD and highest negative docking energy (> − 11 kcal/mol) were extracted as putative potent inhibitors of Sirt2. The primary purpose of the study was to differentiate the most stable conformations of the docked ligands based on their score value which can inhibit the target protein^67^. Remarkably, all the poses of selected drugs showed substantial alignment on the SirReal2 inhibitor native conformation in the X-ray crystal structure, demonstrated the occupancy of docked ligands in the selective pocket of Sirt2. Because intermolecular interactions in the docked protein–ligand complex are indicators to understand the complex stability and provide insights into protein inhibition mechanism^54,68^, molecular contact profiling was extracted for each docked pose of drugs and re-docked SirReal2 inhibitor with Sirt2. Of note, each drug and SirReal2 inhibitor was noted for intermolecular interactions with the hydrophobic cavity of Sirt2 resides by coupling of Rossmann fold domain and zinc-binding domains (Table 1)—a region for the deacylation of ε-amino groups of lysines, as found in the X-ray crystal structure of Sirt2-SirReal2 inhibitor^23^. Interestingly, the binding of SirReal2 inhibitor in the selective pocket has been associated with a disturbance of Sirt2 activity, indicates that screened drugs may exhibit a similar mode of Sirt2 inhibition. As molecular docking is a widely used computational method in structure-based drug design as computationally efficient, low-cost, and simple scoring functions to compute the binding affinity for receptor and ligand under docking simulation, but these methods lack essential energy parameters, such as solvation free energy are completely ignored in most of the docking protocols. Thereof, binding free energies generated via docking scores lack high accuracy^69–73^. This results in associated obscurity to differentiate the docked poses with comparable binding affinities^74^. To eliminate such anomalies, MM/GBSA method has been suggested to evaluate the docked poses for the determination of structural stability and calculation of binding affinities for receptor-ligand complexes^74–76^. Hence, collected docked conformations for selected drugs/SirReal2 inhibitor with Sirt2 were subjected to binding free energy via MM/GBSA method to estimate the net protein–ligand interaction energy^77,78^, revealed the considerable binding affinities of screened drugs with Sirt2 against reference ligand, i.e. SirReal2 inhibitor.

Since the free energy of the system drives all the molecular processes, including molecular association chemical reaction, protein folding, etc., MD simulation of the biomolecular system is the most important method used to determine accurate free energy of the docked complexes^74^. Therefore, in drug discovery, MD simulation is exclusively used to predict the change in docked conformation and interaction profiling at the atomic level^74,79,80^. Of note, MD simulation with the implicit solvent model is generally less reliable and has been observed with dissociation of a ligand from the binding pocket of the receptor^49^. Also, the force field acts a central role in molecular simulation as it governs all the intermolecular interactions of a given system^81^. Thus, all the docked complexes, including Sirt2-drugs and Sirt2-SirReal2 inhibitor poses, were studied under OPLS-2005 force field in explicit (TIP4P) water solvent MD simulation. The MD trajectories demonstrated significant stability and considerable interactions for Ezetimibe, Fluphenazine, Pioglitazone, and Nintedanib drugs docked in the selective pocket of Sirt2; these results highlight the screened drugs as a potential selective putative inhibitor of Sirt2. Also, essential dynamics analysis, generally used for the collection of functional motions in the protein structure via PCA^50,82^, on the respective MD trajectories revealed a substantial increment in residual fluctuations in Sirt2 structure by comparison to free Sirt2 structure and reference docked complex, i.e. Sirt2-SirReal2 inhibitor. Similar results were also documented earlier in docked complexes of G-protein-coupled receptor 119^83^ and Fructose transporter GLUT5^84^. Hence, these observations provide pieces of evidence that screened drugs can significantly disturb the catalytic pocket of Sirt2 by comparison to SirReal2 inhibitor.

To further understand the accuracy and efficiency of the docked inhibitors, end-point free energy calculations are usually computed on MD trajectories in structure-based drug design^74^. Among the various available methods, MM/GBSA method combined with MD simulations to calculate the binding free energy provides a good balance between computational efficiency and accuracy for end-point binding free energy calculations^74^. Herein, Sirt2-Nintedanib complex (− 105.58 ± 7.28 kcal/mol) was established with the most significant end-point free binding energy among the selected Sirt2-drug docked complexes in reference to Sirt2-SirReal2 inhibitor complex (− 99.82 ± 5.21 kcal/mol). Also, as compared to docking scores and binding free energy values, the ranking performance of the screened ligands can be improved by quantum mechanics (QM) treatment on the ligands^46,74^. Besides, a combination of QM/MM molecular docking protocols^85–87^ and MM/GBSA calculations was recently employed to reproduce the crystal structure geometries of protein–ligand complexes with halogen bonding^88^. Also, it has been shown that the prediction of the binding energy can be improved by utilizing dispersion corrected functional for the high-level quantum mechanical calculations as compared to commonly used methods such as B3LYP/DFT and MP2/DFT calculations^46^. Hence, final MD snapshots of the simulated complexes were studied by hybrid QM/MM binding energy, where the ligand-bound region was treated with WB97XD/6-31G** density functional to estimate the non-covalent electronic interaction energies while MM/UFF force field was used for the protein environment. It can be observed that, contrary to docking scores and MM/GBSA binding free energies, the hybrid QM/MM binding energies are most negative (> − 320 kcal/mol) for Sirt2-Fluphenazine and Sirt2-Nintedanib complexes, supports them as strong selective inhibitors of Sirt2. It was estimated that contribution of only electronic energy calculated in this study, which is by the virtue of the molecular geometry and potential energy of the ligands in the selective pocket of Sirt2 protein, comparison with docking scores and binding free energy obtained by molecular mechanics calculations is not an appropriate approach. However, the calculated hybrid QM/MM binding energy can be considered to identify the highly potent ligands from the screened ligand datasets.

Moreover, the application of 3D-QSAR models to quantitatively estimate the activity of new chemical entities has been reported^59,89–91^. A QSAR model is generally defined as a mathematical equation that associates the chemical structure with their biological and physiochemical properties; hence, these models hold the advantage in the activity predictions^92^. Analysis of 3D-QSAR is mainly focused on the variation in 3D structural features such as hydrophobic distribution, electrostatic distribution, hydrogen-bond forming, and orientation of the chemical substituents that affect the biological activity^93,94^. The main advantage of the 3D-QSAR modeling is that it eliminates the problems such as restriction in the estimation of the stereochemistry of the tested dataset and lack of recognition ability in search of active compounds endured by the classical 2D-QSAR^94,95^. Besides, application of 3D-QSAR such as Atom-Based 3D-QSAR model has been suggested as an expedient method against pharmacophore-based 3D-QSAR in that the former scrutinizes the complete molecular space while the latter does not incorporate the area outside the pharmacophore model^96^. Also, Field-Based 3D-QSAR models are known to dispose of the limitations associated with crude approximations linked with 3D structures of aligned ligands by considering the molecule electrostatic, hydrophobic, and steric fields to predict the biological activity or inactivity^62^. Thereof, two different 3D-QSAR approaches, viz. Atom-based 3D-QSAR and Field-Based 3D-QSAR models, were developed and validated using 39 known selective inhibitors of Sirt2 to predict the biological activity of selected FDA-approved drugs as putative inhibitors of Sirt2. Interestingly, both the 3D-QSAR models suggested the Nintedanib drug with the highest activity for Sirt2, which further supports the results of free energy calculations and MD simulations analysis.

In conclusion, we have identified Ezetimibe, Fluphenazine, Pioglitazone, and Nintedanib drugs as the most potent selective and specific inhibitors of Sirt2 protein from FDA-approved drugs using computational calculations which seems to mimic the binding poses and stability of SirReal2 inhibitor to inhibit Sirt2 protein. Moreover, we also established the key intermolecular interactions in the respective complexes and the contribution of dissociation energy components that substantially contributed to the stability of respective docked protein–ligand complexes. The observed stability and activity of screened potent drugs as putative selective inhibitors of Sirt2 protein can be probe by in vitro experiments for the specificity against Sirt2 in reference to other isotypes, such as Sirt1 and Sirt3.

## Conclusion

This study was conducted to discover the selective inhibitors of Sirt2 protein which can disturb the catalytic pocket of the protein by docking in a special hydrophobic pocket at the interface of Rossmann fold domain and zinc-binding domains. After stringent molecular simulations and quantum chemical calculations, Ezetimibe, Fluphenazine, Pioglitazone, and Nintedanib drugs were identified with ideal stability and interactions in the selective pocket of Sirt2. Moreover, conformational changes occurred in the residues, and atoms of the ligands were also mapped in docked complexes during simulations. These details are enormously useful to comprehend the mechanistic insights aspects of the inhibition processes, involved in Sirt2 via screened ligands, and assist to understand the stability and interaction profiles in protein–ligand complexes. Also, developed 3D-QSAR models suggested the substantial activity of selected FDA-approved drugs for Sirt2 inhibition. Since the documented drugs belong to the FDA-approved drug category, respective drugs can be further studied as a novel class of specific and selective Sirt2 inhibitors for exploring their efficacy and regulation in disease-relevant Sirt2 phenotypic disease models.

## Supplementary Information

Supplementary Information.
