# Supplementary material for: Drug repurposing for ligand-induced rearrangement of Sirt2 active site-based inhibitors via molecular modeling and quantum mechanics calculations
Source: Sci Rep. 2021 May 13;11:10169. doi: 10.1038/s41598-021-89627-0 (PMC8119977; doi:10.1038/s41598-021-89627-0)
Supplement: Supplementary file 1 — Supplementary Information. [file 41598_2021_89627_MOESM1_ESM.pdf]

Supplementary information

Drug repurposing for ligand-induced rearrangement of Sirt2 active site-based inhibitors via molecular modeling and quantum mechanics calculations

[1]Shiv Bharadwaj[ǂ], [2]Amit Dubey[ǂ], [3]Nitin Kumar Kamboj, [4]Amaresh Kumar Sahoo*, [1]Sang Gu Kang**, [5]Umesh Yadava***

[1]Department of Biotechnology, Institute of Biotechnology, College of Life and Applied Sciences, Yeungnam University, 280 Daehak-Ro, Gyeongsan, Gyeongbuk 38541, Republic of Korea

[2]Computational Chemistry and Drug Discovery Division, Quanta Calculus Pvt. Ltd., Kushinagar 274203, India

[3]School of Physical Sciences, DIT University, Dehradun, UK, 248001 India

[4]Department of Applied Sciences, Indian Institute of Information Technology Allahabad, Allahabad 211015, Uttar Pradesh, India

[5]Department of Physics, Deen Dayal Upadhyay Gorakhpur University, Gorakhpur, India.

[ǂ]Authors contributed equally.

***Corresponding authors**

AKS: asahoo@iiita.ac.in

SGK: kangsg@ynu.ac.kr

UY: u_yadava@yahoo.com

S1. Results

S1.1. Extra precision docking and binding pose analysis

Table S1. List of FDA-approved drugs collected after extra precision molecular docking in the selective pocket of Sirt2.

S.no.	Drug	RMSD	Docking score (kcal/mol)	Generic name	Approved for diseases
1	ZINC000043207238	0.009	-14.499	Canagliflozin	Type 2 diabetes mellitus
2	ZINC000052716421	0.045	-13.203	Flibanserin	Hypoactive sexual desire disorder (HSDD)
3	ZINC000003810860	0.026	-12.784	Ezetimibe	High blood cholesterol
4	ZINC000004175630	0.038	-12.731	Pimozide	Tourette syndrome
5	ZINC000019203912	0.049	-12.619	Fluphenazine	Schizophrenia and psychotic symptoms
6	ZINC000019796080	0.02	-12.177	Droperidol	Antipsychotic
7	ZINC000098023177	0.027	-12.143	Osimertinib	Non-small-cell lung carcinomas
8	ZINC000000968326	0.012	-12.091	Pioglitazone	Type 2 diabetes
9	ZINC000000897256	0.041	-12.037	Formoterol	COPD
10	ZINC000100014909	0.039	-11.886	Nintedanib	Idiopathic pulmonary fibrosis?
11	ZINC000000968328	0.041	-11.844	Rosiglitazone	Type 2 diabetes
12	ZINC000001550477	0.007	-11.747	Lapatinib	Breast cancer and other solid tumors
13	ZINC000003800008	0.009	-11.699	Dostinex	Dopamine receptor agonist on D_2 receptors
14	ZINC000019418959	0.015	-11.644	Trifluoperazine	Schizophrenia
115	ZINC000000968327	0.032	-11.636	Pioglitazone	Type 2 diabetes
16	ZINC000000643153	0.022	-11.608	Nizoral	Antifungal medication
17	ZINC000001550499	0.047	-11.601	Cinacalcet	Tertiary hyperparathyroidism
18	ZINC000003823475	0.023	-11.346	Eletriptan	Migraine
19	ZINC000011681534	0.048	-11.313	Nebivolol	High blood pressure and heart failure
20	ZINC000019632618	0.035	-11.179	Imatinib	Anti-cancer
21	ZINC000003932831	0.01	-10.958	Dutasteride	Antiandrogen
22	ZINC000019594557	0.048	-10.939	Meclizine	Antihistamine
23	ZINC000028957444	0.021	-10.925	Brilinta	Antagonist of the P2Y(12) receptor
24	ZINC000084441937	0.005	-10.733	Tetracycline	Antibiotic
25	ZINC000000606383	0.044	-10.637	Sertaconazole	Antifungal medication
26	ZINC000001530912	0.008	-10.504	Emedastine	Antihistamine
27	ZINC000001530579	0.013	-10.446	Coreg	High blood pressure
28	ZINC000001895505	0.036	-10.011	Bazedoxifene	Selective estrogen receptor modulator
29	ZINC000003802417	0.045	-9.982	Entereg	Postoperative ileus (POI)
30	ZINC000019796158	0.004	-9.878	Loxapine	Schizophrenia
31	ZINC000004474405	0.03	-9.768	Latisse	High blood pressure and glaucoma
32	ZINC000094566093	0.036	-9.707	Doxazosin	enlarged prostate and high blood pressure
33	ZINC000003776633	0.011	-9.004	Acrivastine	?allergies and hay fever.
34	ZINC000000538658	0.017	-8.641	Samsca	congestive heart failure,
35	ZINC000052955754	0.013	-8.537	Erqotamine	acute migraine attacks.
36	ZINC000060325170	0.028	-8.462	cobimetinib	Melanoma
37	ZINC000070466416	0.046	-8.254	cabozantinib	Medullary thyroid cancer andrenal cell carcinoma
38	ZINC000000596731	0.012	-7.786	Tikosvn	Antiarrhythmic agent.
39	ZINC000000538483	0.02	-7.3	Trazodone	Antidepressant medication
40	ZINC000003776970	0.041	-6.628	Cefprozil Anhydrous)	Ear infections, skin infections, and bacterial infections
41	ZINC000022443609	0.02	-5.913	Plerixafor	Immunostimulant
42	ZINC000003874185	0.05	-5.802	Mefloquine	Anti-malaria
43	ZINC000006716957	0.015	-2.913	Nilotinib	Chronic myeloid leukemia (CML).

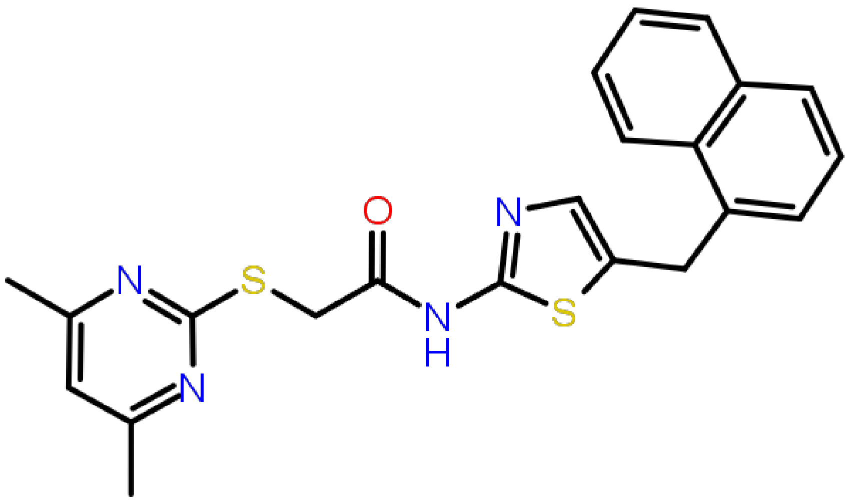

Figure S1. 2D structural formula for the co-crystallized compound, i.e. 2-[(4,6-dimethylpyrimidin-2-yl)sulfanyl]-N-[5-(naphthalen-1-ylmethyl)-1,3-thiazol-2-yl]acetamide or SirReal2 inhibitor selected as reference ligand against FDA approved drugs for the selective pocket of Sirt2. 2D image is sketched using academic Schrödinger-Maestro v12.4 suite [43] (URL: https://www.schrodinger.com/freemaestro)

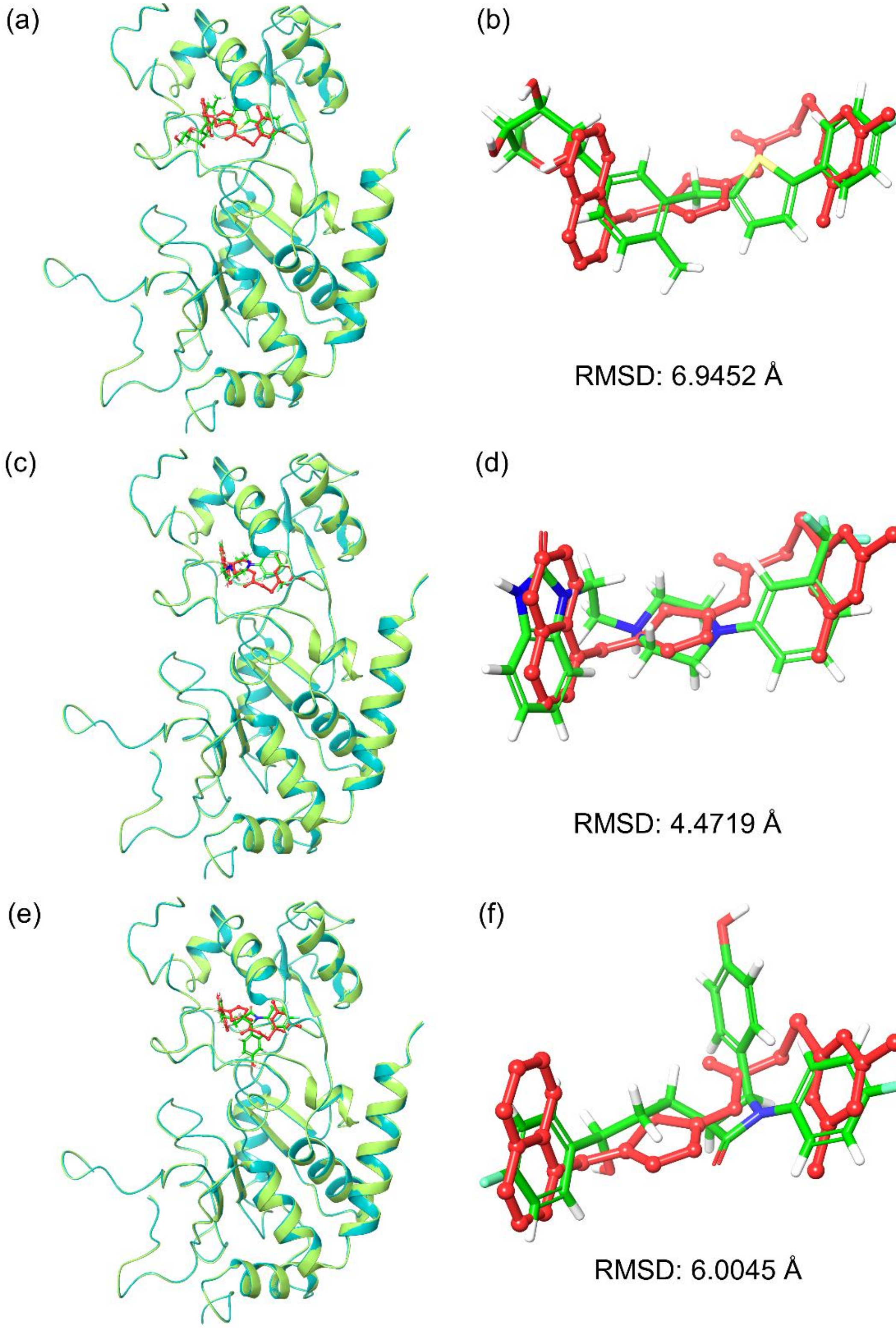
(a)
(b)
RMSD: 6.9452 Å
(c)
(d)
RMSD: 4.4719 Å
(e)
(f)
RMSD: 6.0045 Å

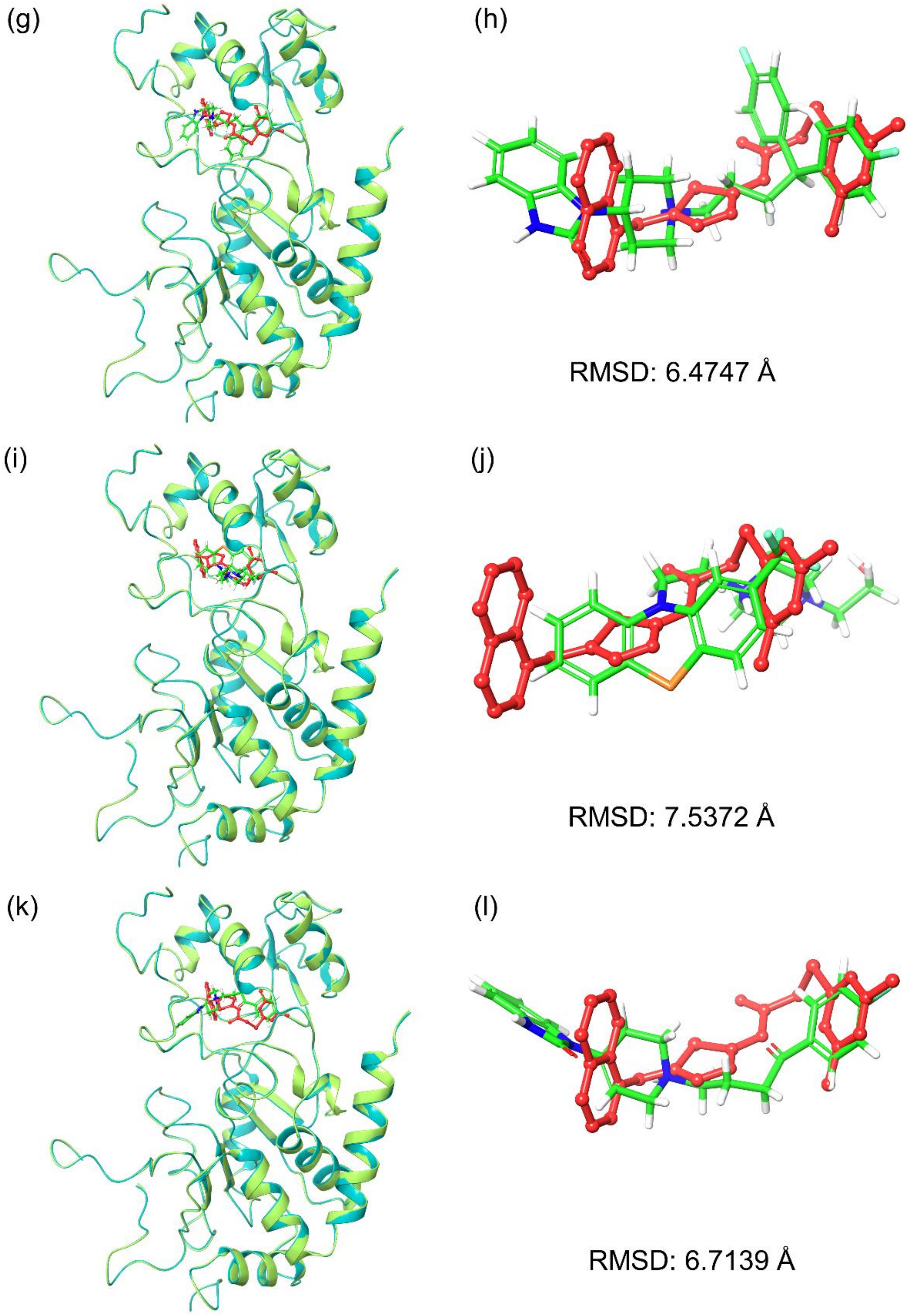
(g)
(h)
RMSD: 6.4747 Å
(i)
(j)
RMSD: 7.5372 Å
(k)
(l)
RMSD: 6.7139 Å

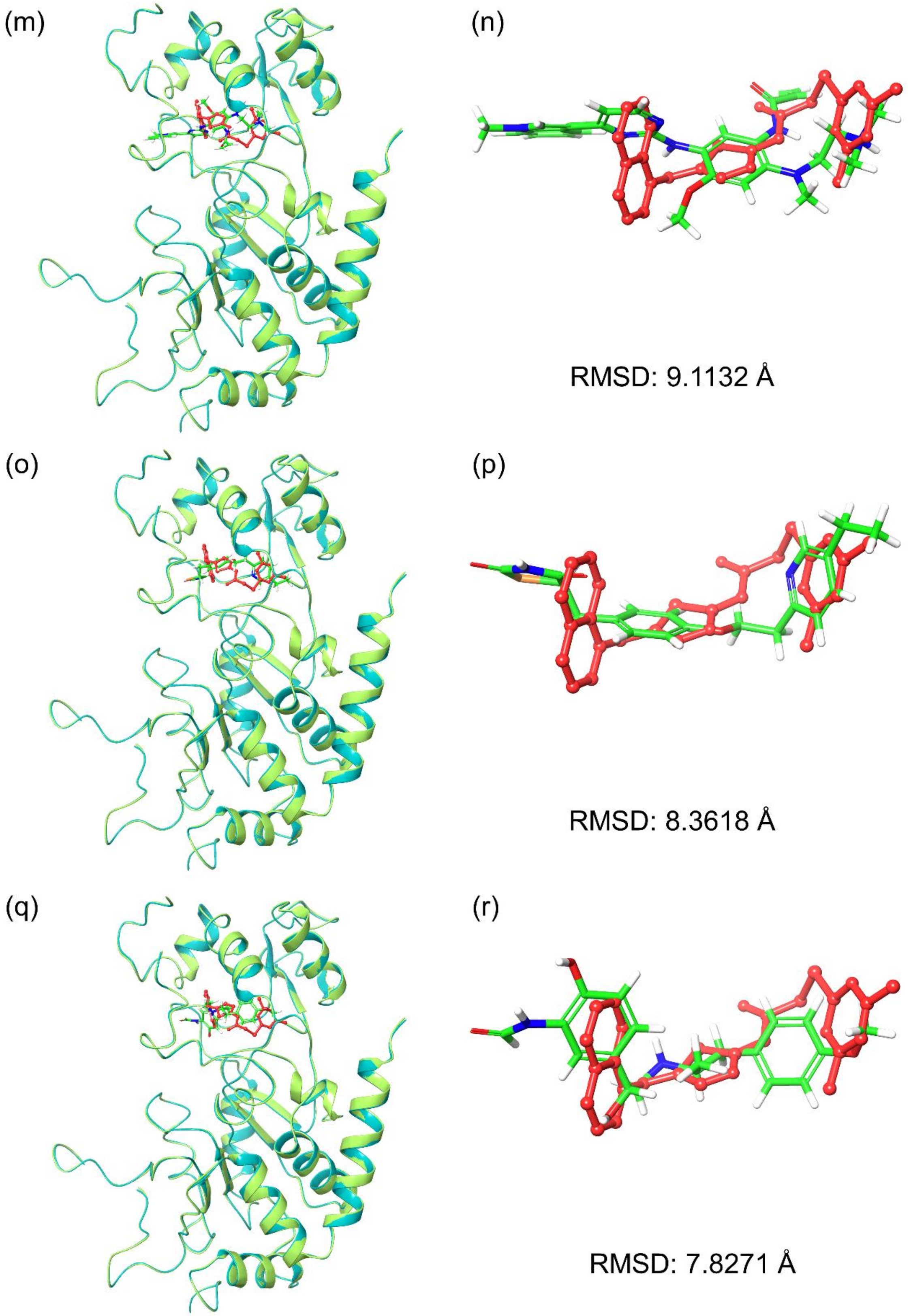
(m)
(n)
RMSD: 9.1132 Å
(o)
(p)
RMSD: 8.3618 Å
(q)
(r)
RMSD: 7.8271 Å

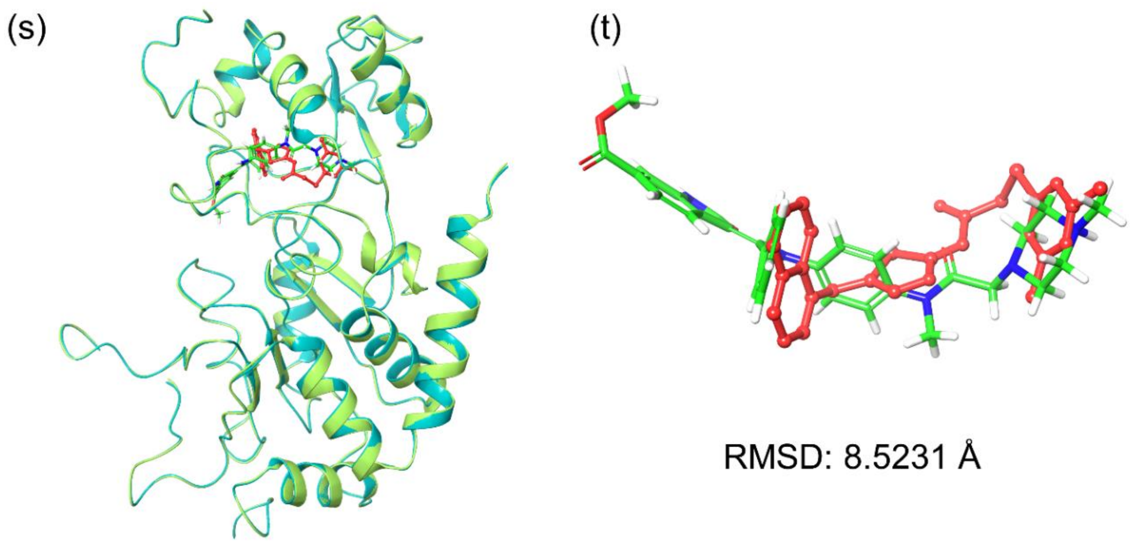

Figure S2. Superimposed molecular docked poses of Sirt2 protein (green color) with selected FDA-approved drugs (green color), i.e. (a-b) Canagliflozin, (c-d) Flibanserin, (e-f) Ezetimibe, (g-h) Pimozide, (i-j) Fluphenazine, (k-l) Droperidol, (m-n) Osimertinib, (o-p) Pioglitazone, (q-r) Formoterol, and (s-t) Nintedanib, as selective inhibitors on co-crystalized SirReal2 (red color) in crystal structure of Sirt2 (cyan color). Herein, complex alignment was performed with respect to ligand while position conformation for the aligned ligands were calculated in terms of RMSD values. Images were rendered using academic Schrödinger-Maestro v12.4 suite[43] (URL: https://www.schrodinger.com/freemaestro)

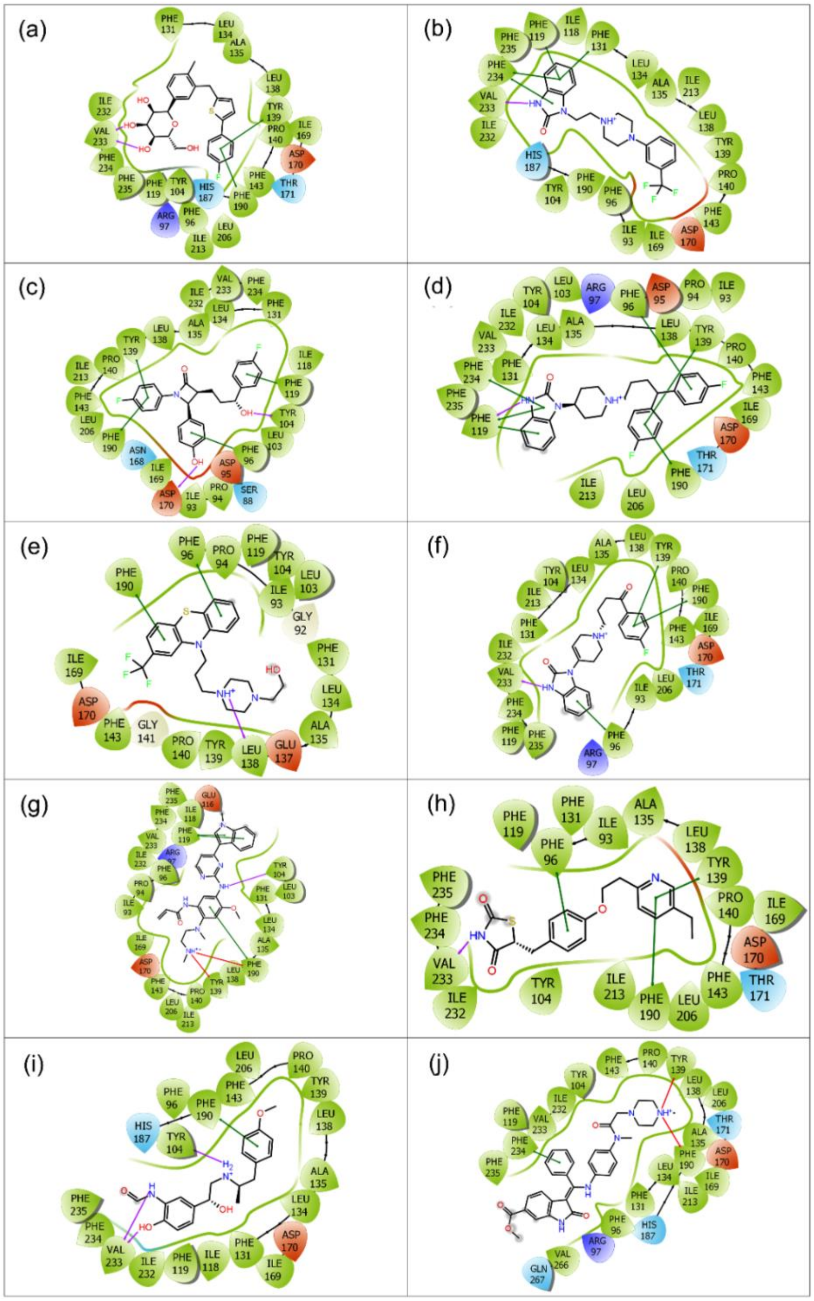

Figure S3. 2D molecular contact map for the molecular docked selected FDA-approved drugs, viz. (a) Canagliflozin, (b) Flibanserin, (c) Ezetimibe, (d) Pimozide, (e) Fluphenazine, (f) Droperidol, (g) Osimertinib, (h) Pioglitazone, (i) Formoterol, and (j) Nintedanib, in the selective pocket of Sirt2. The docked complexes exhibited hydrogen bond (pink arrows), π-π (green lines), π-cation (red lines), hydrophobic (green), polar (blue), negative (red), positive (violet) and glycine (grey) interactions are depicted for respective docked complexes. Images were rendered using academic Schrödinger-Maestro v12.4 suite[43] (URL: https://www.schrodinger.com/freemaestro)

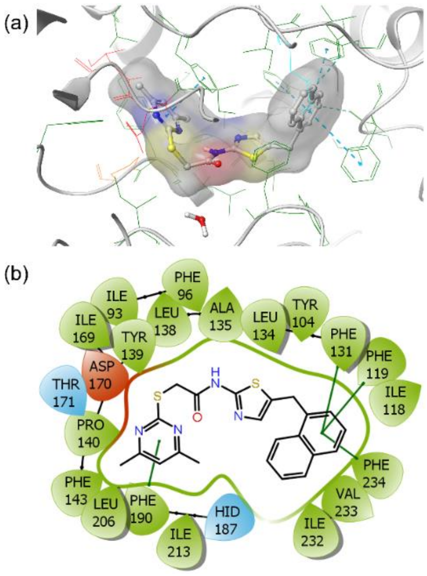

Figure S4. 3D and 2D molecular contact map for the molecular docked SirReal2 inhibitor as reference ligand in the selective pocket of Sirt2. In 2D interaction map, the docked complexes exhibited π-π (green lines), hydrophobic (green), polar (blue), and negative (red) interactions are depicted for respective docked complexes. Images were rendered using academic Schrödinger-Maestro v12.4 suite[43] (URL: https://www.schrodinger.com/freemaestro)

S1.2. Molecular mechanics/generalized Born surface area analysis

Table S2. Molecular mechanics generalized Born surface area (MM/GBSA) binding free energy and dissociation energy components (kcal/mol) computed for the selected drugs as selective inhibitors of Sirt2 against reference ligand on the respective molecular docked poses before molecular dynamics simulation.

MMGBSA Components (kcal/mol)	Canagliflozin	Flibanserin	Ezetimibe	Pimozide	Fluphenazine	Droperidol	Osimertinib	Pioglitazone	Formoterol	Nintedanib	SirReal2
ΔG_{Bind}	-73.26	-64.75	-61.33	-73.31	-73.97	-73.73	-65.54	-67.18	-69.14	-78.65	-96.22
$\Delta G_{Bind\ Coulomb}$	-12.61	-60.46	-5.84	-47.75	-57.55	-41.75	-53.3	-10.91	-65.36	-42.8	-9.9
$\Delta G_{Bind\ Covalent}$	11.95	8.33	11.17	13.25	3.81	4.88	9.07	3.81	7.94	13.05	0.95
$\Delta G_{Bind\ Hbond}$	-1.03	-0.03	-1.11	-0.23	-1.02	-0.71	-0.79	-0.53	-1.19	-0.39	-0.01
$\Delta G_{Bind\ Lipo}$	-46.79	-39.23	-47.51	-52.19	-38.31	-34.25	-36.03	-37.89	-32.38	-49.74	-46.43
$\Delta G_{Bind\ Packing}$	-5.19	-6.35	-5.6	-6.44	-3.05	-6.03	-5.5	-4.54	-2.93	-2.19	-6.94
$\Delta G_{Bind\ Solv\ GB}$	30.64	73.82	29.88	72.54	66.87	53.41	78.5	23.73	70.06	68.07	37.26
$\Delta G_{Bind\ vdW}$	-50.24	-40.82	-42.32	-52.49	-44.72	-49.29	-57.49	-40.86	-45.28	-64.64	-71.17

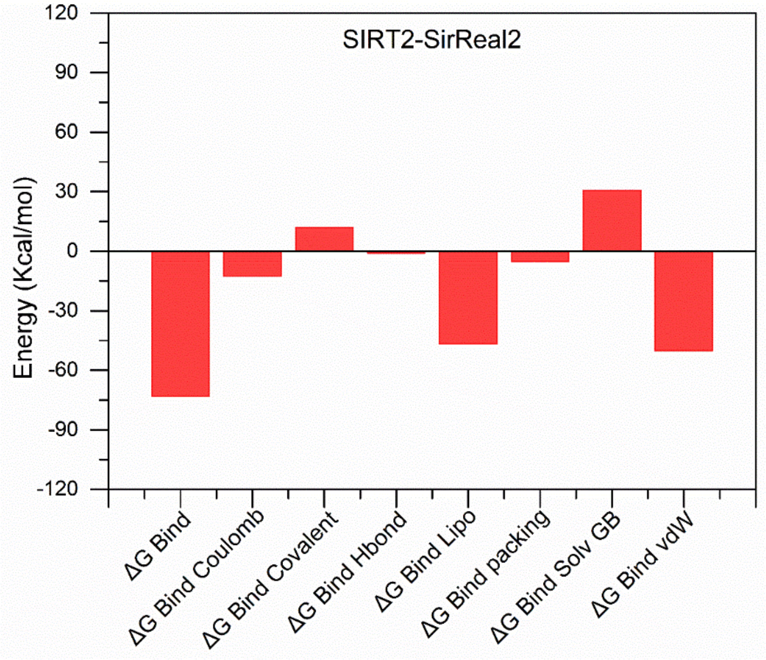

Figure S5. Graphical representation for the binding free energy and individual dissociation energy components calculated for the docked complex of SirReal2 inhibitor with Sirt2 using MM/GBSA method.

S1.3. Explicit solvent molecular dynamics simulation analysis

S1.3.1. Last pose and intermolecular interaction profiling

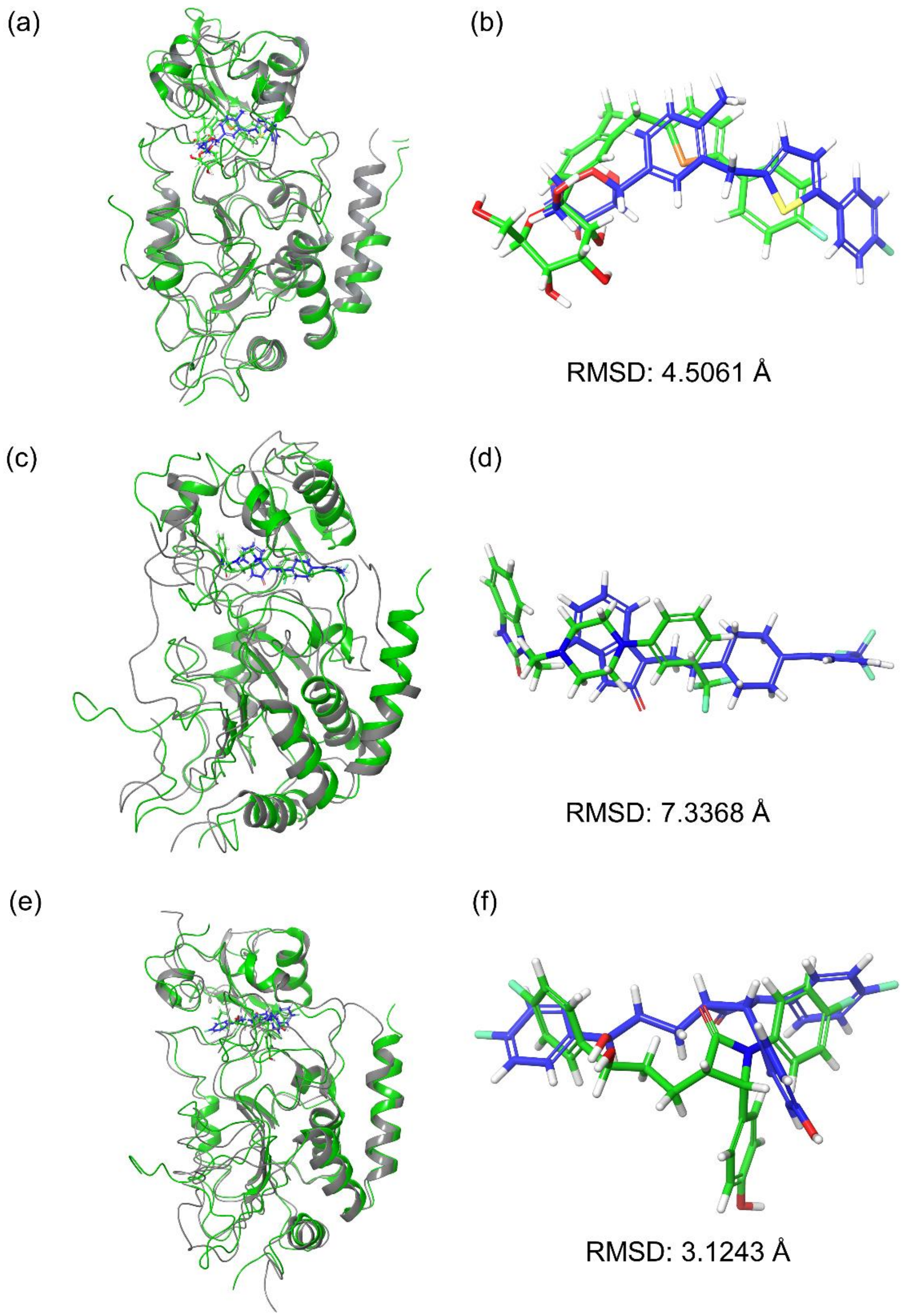

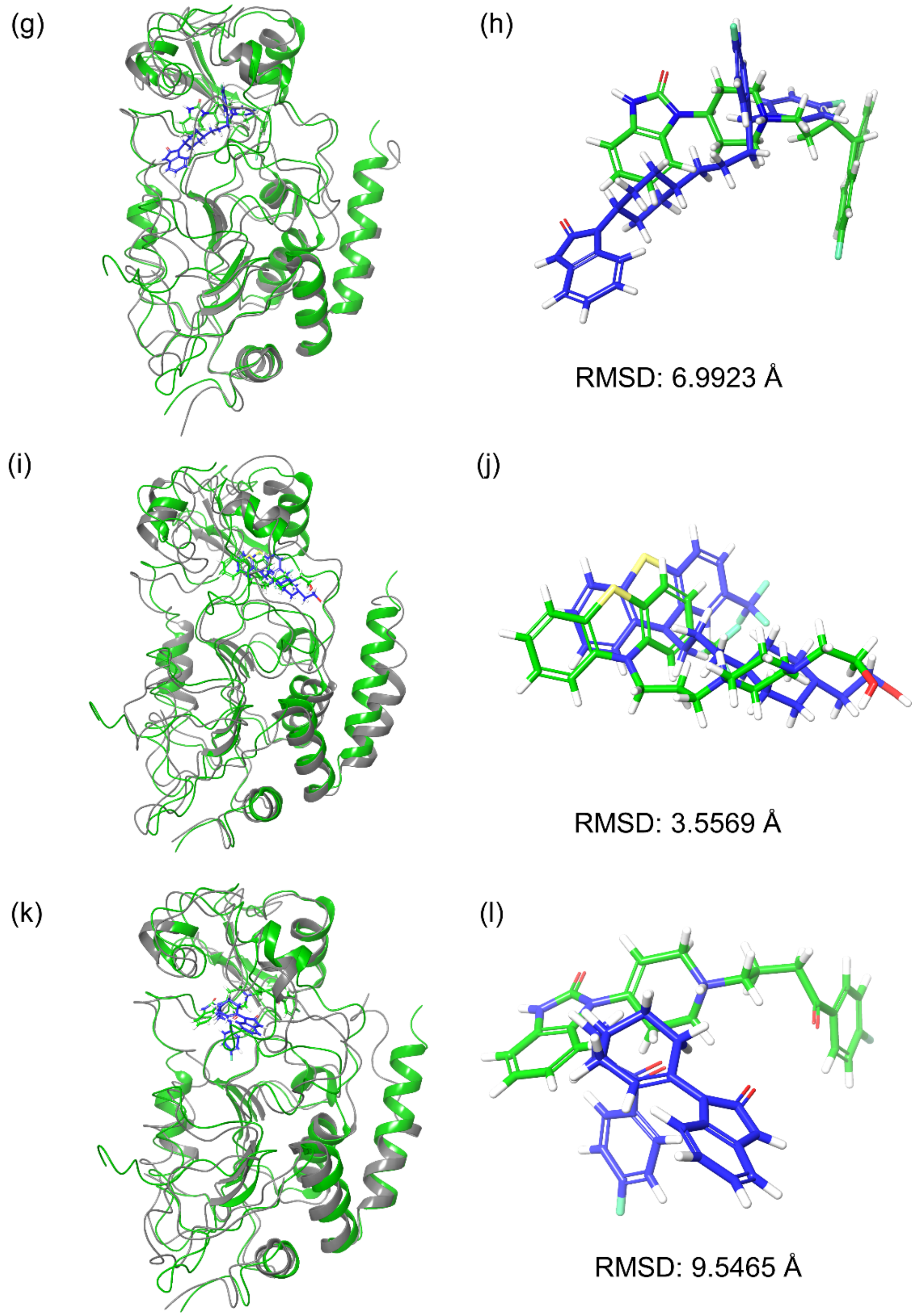
(g)
(h)
RMSD: 6.9923 Å
(i)
(j)
RMSD: 3.5569 Å
(k)
(l)
RMSD: 9.5465 Å

(m)

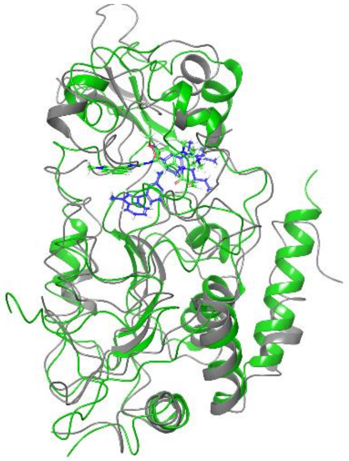

(n)

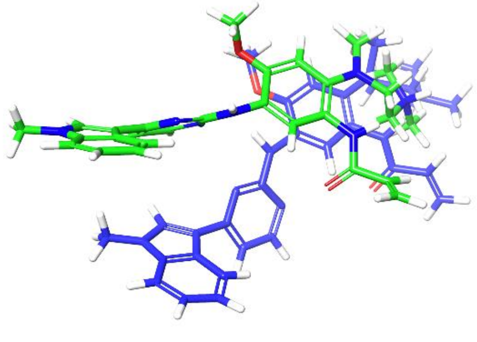

RMSD: 5.6512 Å

(o)

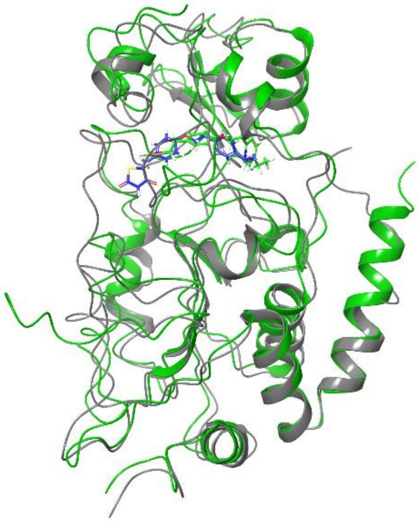

(p)

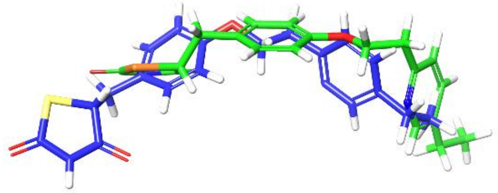

RMSD: 4.3583 Å

(q)

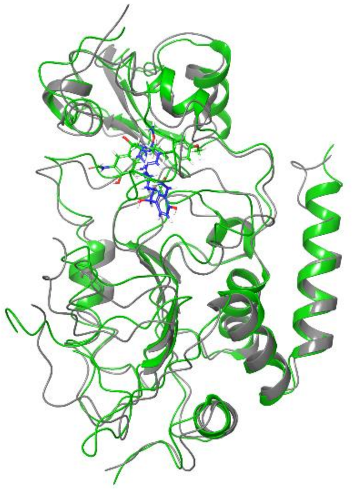

(r)

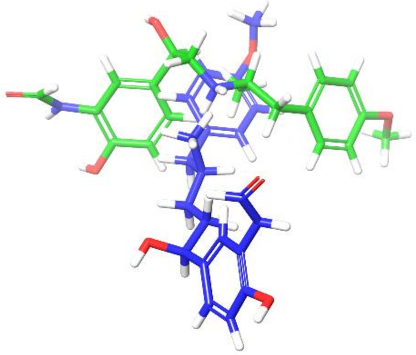

RMSD: 8.4034 Å

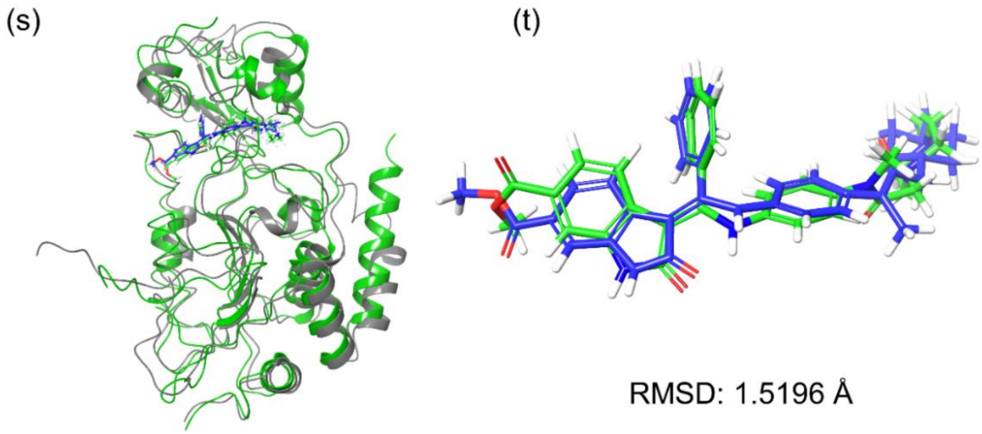

Figure S6. Superimposed molecular docked (green color ligand and protein) poses on the respective last snaps (blue color ligand and gray color protein) from 100 ns MD simulation for the Sirt2 complexes with selected drugs, i.e. (a-b) Canagliflozin, (c-d) Flibanserin, (e-f) Ezetimibe, (g-h) Pimozide, (i-j) Fluphenazine, (k-l) Droperidol, (m-n) Osimertinib, (o-p) Pioglitazone, (q-r) Formoterol, and (s-t) Nintedanib. Herein, pose alignment was performed with respect to ligand and respective RMSD values crossponding to change in conformation were calculated. Images were rendered using academic Schrödinger-Maestro v12.4 suite[43] (URL: https://www.schrodinger.com/freemaestro)

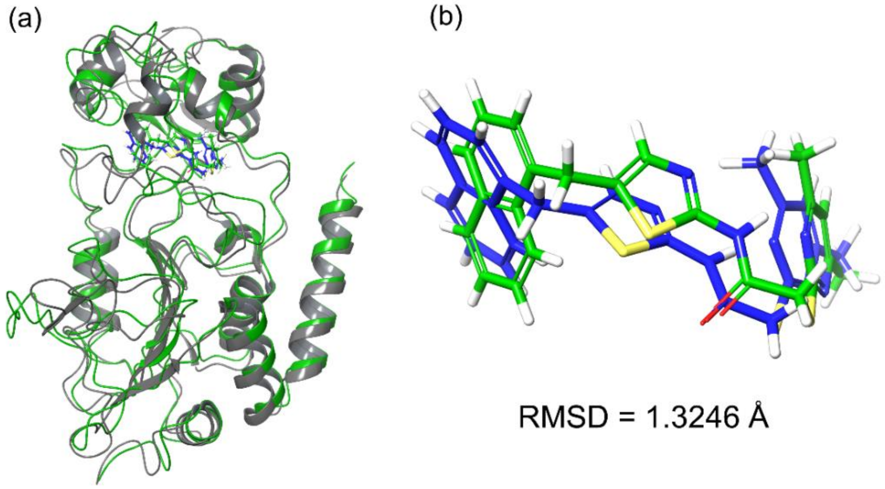

Figure S7. Superimposed molecular docked complex (green color ligand and protein) on the last snap (blue color ligand and gray color protein) extracted from 100 ns MD simulation for the Sirt2 -SirReal2 inhibitor complex. Herein, pose alignment was performed with respect to ligand

and respective RMSD values crossponding to change in conformation were calculated. Images were rendered using academic Schrödinger-Maestro v12.4 suite[43] (URL: https://www.schrodinger.com/freemaestro)

Table S3. List of intermolecular interactions in the extracted last snapshot of Sirt2 complexes with selected drugs and SirReal2 inhibitor after 100 ns molecular dynamics simulation.

S. no.	Compound	H-Bond	π-π/ *π- cation stacking	Hydrophobic	Polar	Negative	Positive	Glycine /*salt bridge
**1**	Canagliflozin	Val^{233}(2)	Phe^{96}, Tyr^{139}, Phe^{190}	Phe^{96}, Tyr^{104}, Ile^{118}, Phe^{119}, Phe^{131}, Leu^{134}, Ala^{135}, Leu^{138}, Tyr^{139}, Pro^{140}, Phe^{143}, Ile^{169},Leu^{172}, Phe^{190}, Leu^{206}, Ile^{213}, Ile^{232}, Val^{233}, Phe^{234}, Phe^{235}	Thr^{171}, His^{187}	Asp^{170}	Arg^{97}	--
**2**	Flibanserin	Asp^{170}	Tyr^{139}, *Tyr^{139}	Ile^{93}, Phe^{96}, Ile^{118}, Phe^{119}, Phe^{131}, Phe^{132}, Leu^{134}, Ala^{135}, Leu^{138}, Tyr^{139}, Pro^{140}, Phe^{143}, Ile^{169}, Leu^{172}, Phe^{190}, Tyr^{191}, Leu^{206}, Ile^{213}, Ile^{352}, Ala^{354}	Gln^{142}, Gln^{355}, Ser^{356}	Asp^{353}, Asp^{170}	Arg^{97}	Gly^{141}, *Asp^{170}
**3**	Ezetimibe	Pro^{94}	Phe^{119}, His^{187}, Phe^{190}	Ile^{93}, Pro^{94}, Phe^{96}, Phe^{119}, Phe^{131}, Leu^{134}, Ala^{135}, Leu^{138}, Tyr^{139}, Pro^{140}, Phe^{143}, Ile^{169}, Phe^{190}, Leu^{206}, Phe^{214}, Ile^{232}, Val^{233}, Phe^{234}, Phe^{235}	Thr^{171}, His^{187}	Asp^{95}, Asp^{170}	Lys^{210}	--
**4**	Pimozide	--	Phe^{119} *Phe^{119}	Phe^{96}, Leu^{103}, Tyr^{104}, Ile^{118}, Phe^{119}, Phe^{131}, Leu^{134}, Ala^{135}, Leu^{138}, Tyr^{139}, Pro^{140}, Ile^{169}, Phe^{190}, Ile^{232}, Val^{233}, Phe^{235}, Val^{266}, Leu^{297}, Phe^{296}	Asn^{106}, His^{187}, Gln^{267}	--	Arg^{97}	--
**5**	Fluphenazine	Lys^{144}, Asp^{170}	--	Ile^{93}, Pro^{94}, Phe^{96}, Leu^{103}, Ile^{118}, Phe^{119}, Leu^{134}, Ala^{135}, Leu^{138}, Tyr^{139}, Pro^{140}, Phe^{143}, Ile^{169}, Leu^{172}, Phe^{190}, Tyr^{191}, Leu^{206}	Gln^{142}	Asp^{95}, Asp^{170}	Lys^{144}	*Asp^{170}
**6**	Droperidol	Ser^{98}, Glu^{116}	Phe^{96}, *Glu^{116}	Ala^{85}, Phe^{96}, Pro^{99}, Leu^{103}, Tyr^{104}, Phe^{119}, Leu^{134}, Leu^{138}, Ile^{169}	Ser^{98}, Ser^{100}, Gln^{167}, His^{187}	Asp^{95}, Glu^{116}	Arg^{97}	Glu^{116}
**7**	Osimertinib	Leu^{138}, , Asp^{170}	His^{187}, *Tyr^{139}	Ala^{85}, Ile^{93}, Phe^{96}, Ile^{118}, Phe^{119}, Phe^{131}, Ala^{135}, Leu^{138}, Tyr^{139}, Pro^{140}, Phe^{143}, Ile^{169}, Phe^{190}, Leu^{206}, Pro^{230}, Ile^{232}, Phe^{235}, Val^{266}, Leu^{297}	Gln^{167}, Asn^{168}, His^{187}	Asp^{95}, Glu^{116}, Asp^{170}	--	*Asp^{170}
**8**	Pioglitazone	--	Phe^{96} (2), Phe^{190}	Ile^{93}, Phe^{96}, Ile^{118}, Phe^{119}, Phe^{131}, Ala^{135}, Leu^{138}, Tyr^{139}, Pro^{140}, Phe^{143}, Ile^{169}, Phe^{190}, Ile^{232}, Val^{233}, Phe^{234}, Phe^{235}, Leu^{297}	His^{187}	Asp^{170}	Arg^{97}	--
**9**	Formoterol	Tyr^{104}, Val^{233}(2)	Phe^{119}, Phe^{190}	Phe^{96}, Tyr^{104}, Ile^{118}, Phe^{119}, Phe^{131}, Leu^{134}, Ala^{135}, Leu^{138}, Tyr^{139}, Pro^{140}, Phe^{143}, Ile^{169}, Phe^{190}, Leu^{206}, Ile^{232}, Val^{233}, Phe^{234}, Phe^{235}	His^{187}	Glu^{116}, Asp^{170}	Arg^{97}	--
**10**	Nintedanib	Asp^{170}	His^{187}, Phe^{234}, *Tyr^{139}	Phe^{96}, Leu^{103}, Ile^{118}, Phe^{119}, Phe^{131}, Leu^{134}, Ala^{135}, Leu^{138}, Tyr^{139}, Pro^{140}, Ile^{169}, Phe^{190}, Leu^{206}, Ile^{232}, Val^{233}, Phe^{234}, Phe^{235}	Thr^{101}, His^{187}	Asp^{170}, Glu^{237}	Arg^{97}	Gly^{236},* Asp^{170}
**11**	SirReal2 inhibitor	--	Phe^{119}, Phe^{131}, Phe^{234}	Ile^{93}, Pro^{94}, Phe^{96}, Leu^{103}, Ile^{118}, Phe^{119}, Phe^{131}, Leu^{134}, Ala^{135}, Leu^{138}, Tyr^{139}, Pro^{140}, Phe^{143}, Ile^{169}, Phe^{190}, Leu^{206}, Ile^{213}, Ile^{232}, Val^{233}, Phe^{234}	His^{187}	Asp^{170}	--	--

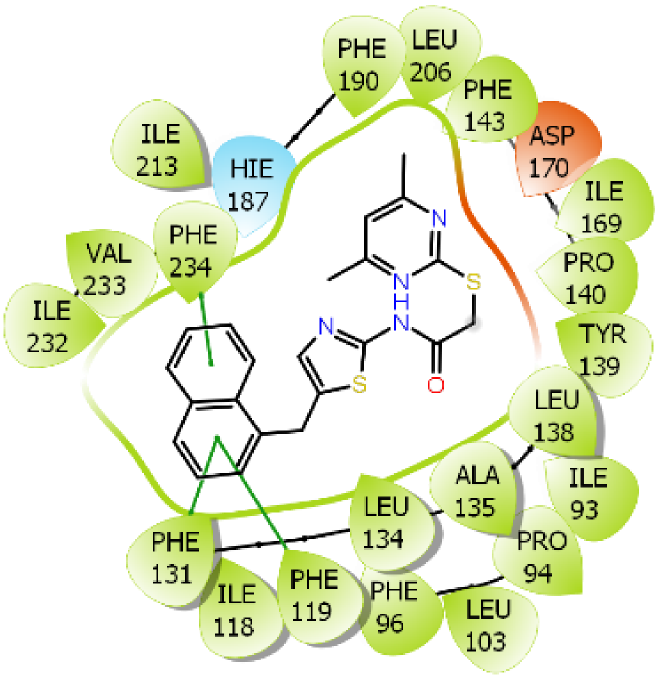

Figure S8. 2D molecular contact profiling for last poses of Sirt2-SirReal2 inhibitor after 100 ns MD simulation. Herein, π-π (green lines), hydrophobic (green), polar (blue), and negative (red) interactions are depicted between ligand and residues of the protein. Images were rendered using academic Schrödinger-Maestro v12.4 suite[43] (URL: https://www.schrodinger.com/freemaestro)

S1.3.2. RMSD and RMSF analysis

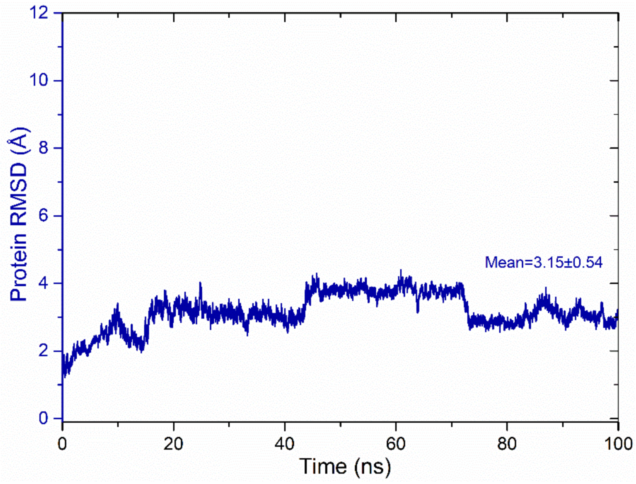

Figure S9. Calculated RMSD values for alpha carbon atoms (blue curves) of Sirt2 protein only plotted as function of 100 ns simulation interval.

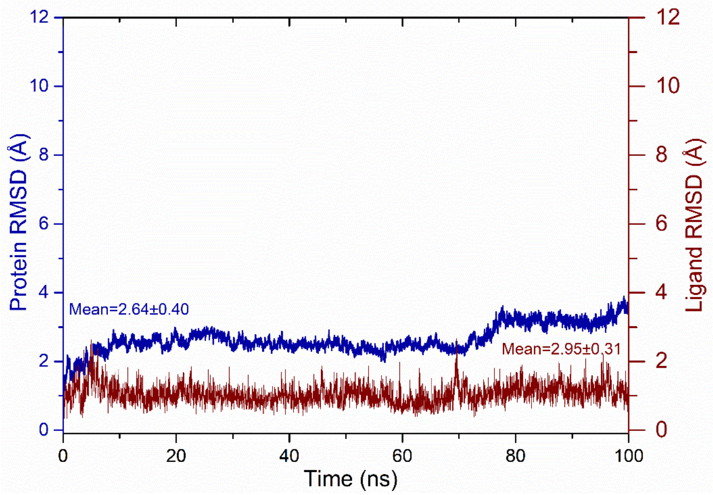

Figure S10. Calculated RMSD values for alpha carbon atoms (blue curves) of Sirt2 and reference ligand SirReal2 (red curves) plotted as function of 100 ns simulation interval.

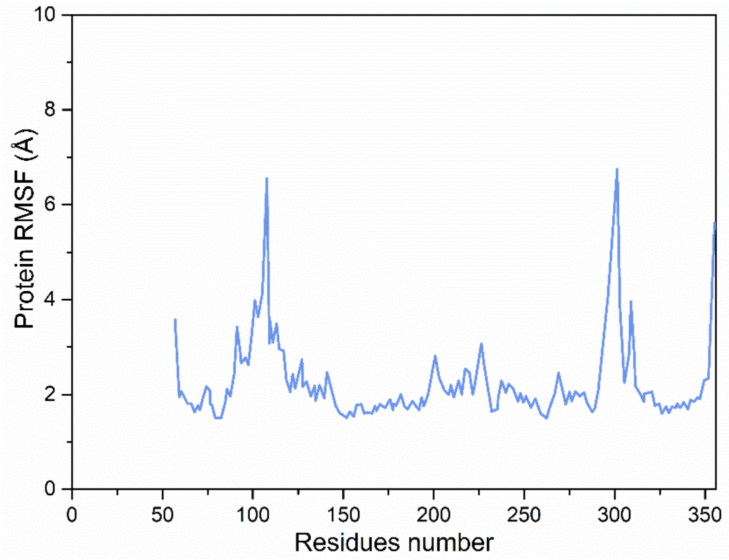

Figure S11. Protein RMSF plots extracted for free Sirt2 protein from 100 ns molecular dynamics simulation

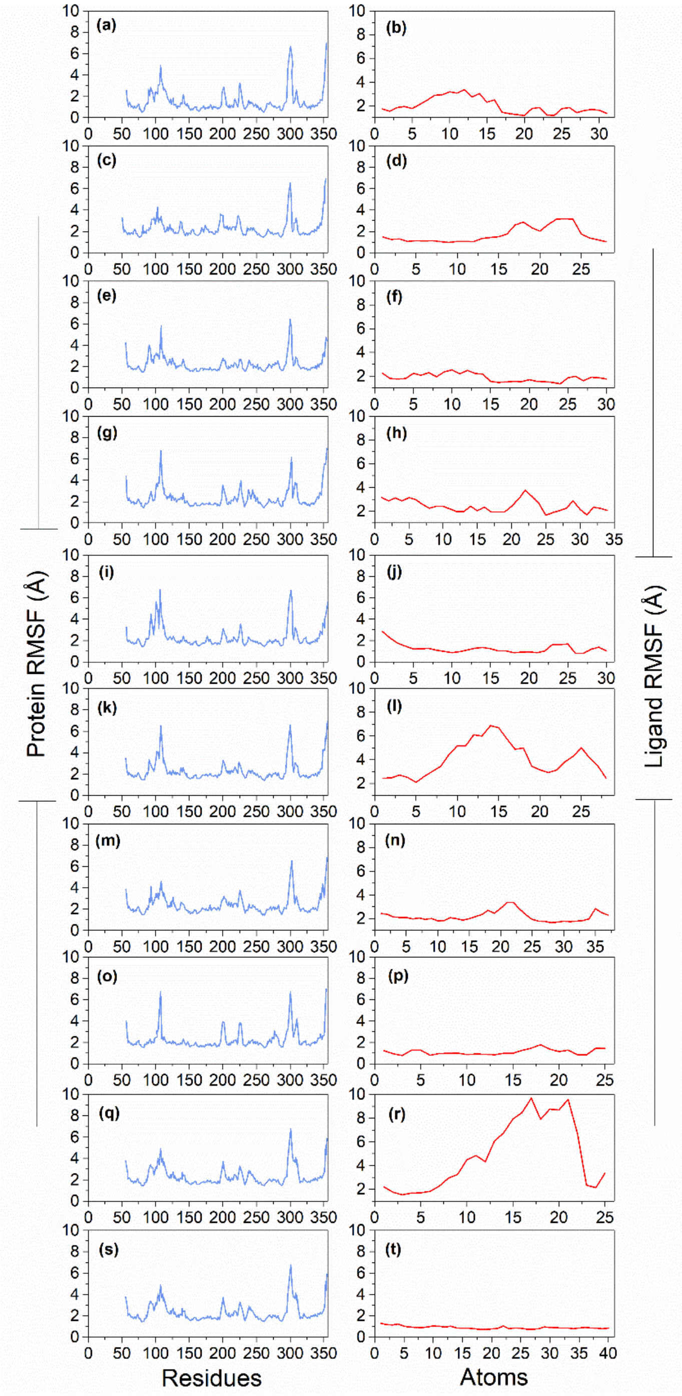

Figure S12. Protein and Ligand RMSF plots extracted for the docked complexes of Sirt2 with selected drugs, i.e. (a-b) Canagliflozin, (c-d) Flibanserin, (e-f) Ezetimibe, (g-h) Pimozide, (i-j) Fluphenazine, (k-l) Droperidol, (m-n) Osimertinib, (o-p) Pioglitazone, (q-r) Formoterol, and (s-t) Nintedanib, from 100 ns molecular dynamics simulation.

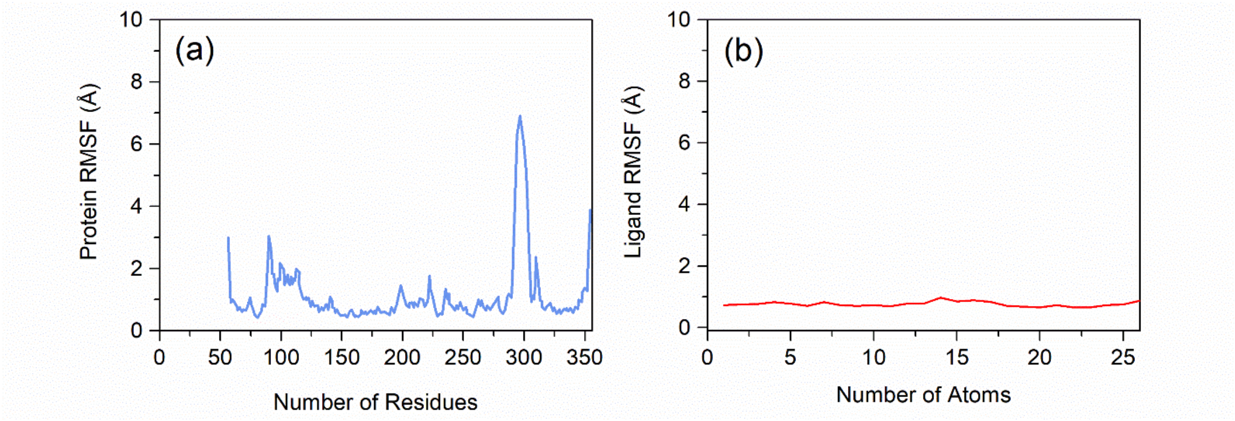

Figure S13. Protein and Ligand RMSF plots extracted for the docked complexes of SIRT2 with SirReal2 during 100 ns simulation interval.

S1.3.3. Protein-ligand interaction mapping

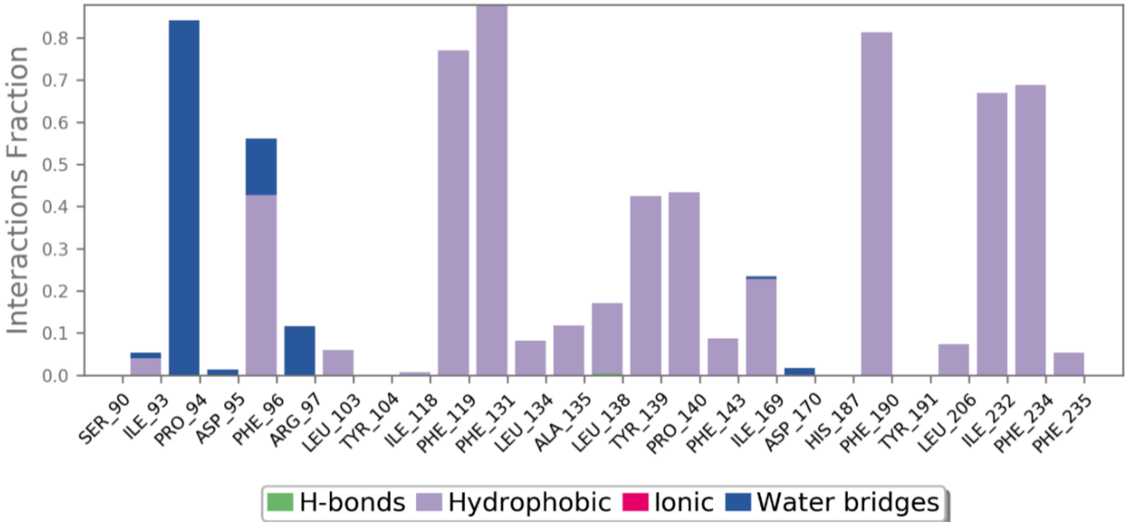

Figure S14. Protein-ligand interactions mapping for Sirt2 with SirReal2 extracted from respective 100 ns molecular dynamics simulations trajectories.

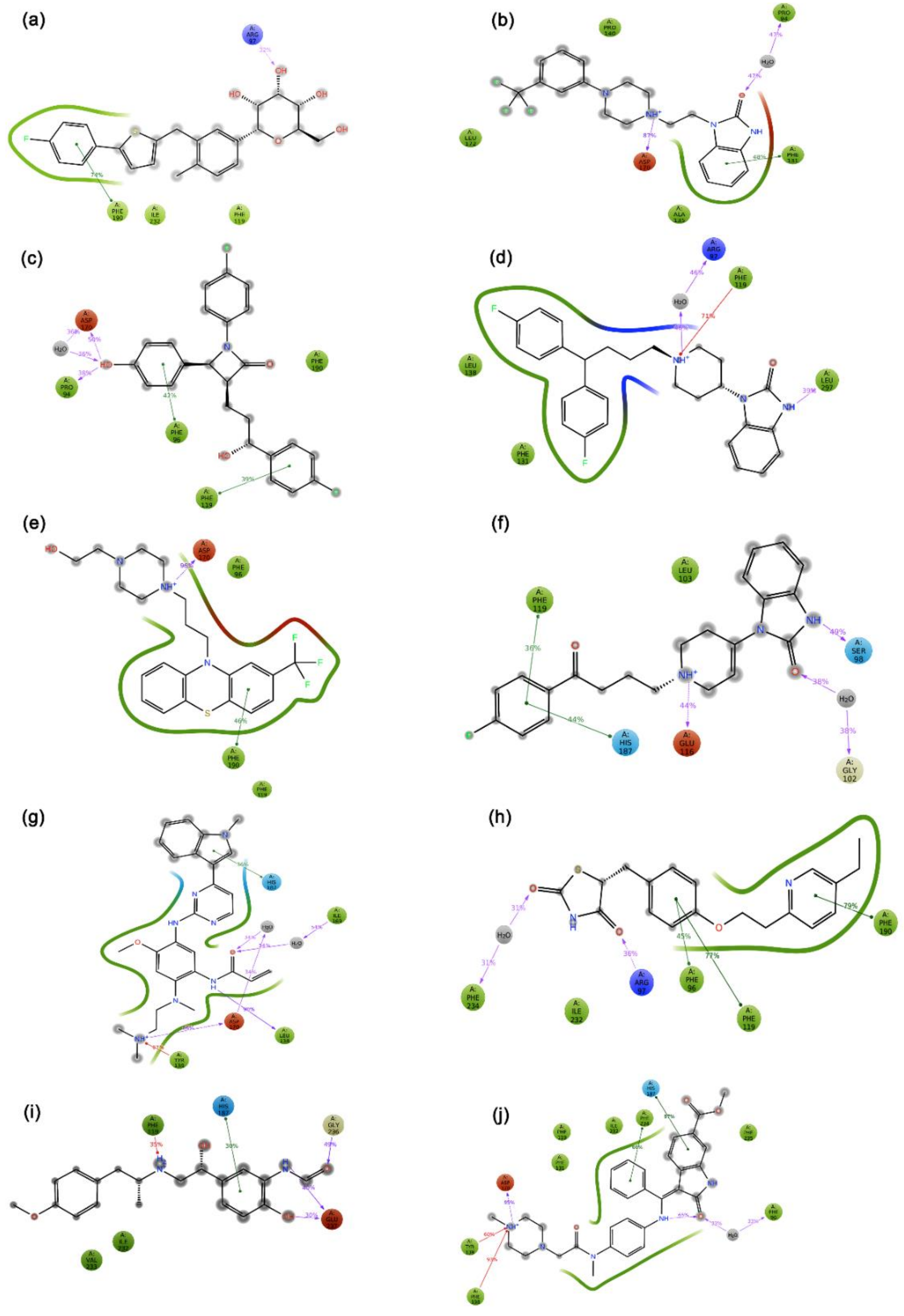

Figure S15. 2D protein-ligand interaction maps extracted for the selected drugs, viz. (a) Canagliflozin, (b) Flibanserin, (c) Ezetimibe, (d) Pimozide, (e) Fluphenazine, (f) Droperidol, (g)

Osimertinib, (h) Pioglitazone, (i) Formoterol, and (j) Nintedanib, in the selective pocket of Sirt2. Herein, hydrogen bond (pink arrows), π-π (green lines), π-cation (red lines), hydrophobic (green), polar (blue), negative (red), and positive (violet) interactions are captured for respective complexes at 30% of the total molecular dynamics simulation. Images were rendered using academic Schrödinger-Maestro v12.4 suite[43] (URL: https://www.schrodinger.com/freemaestro)

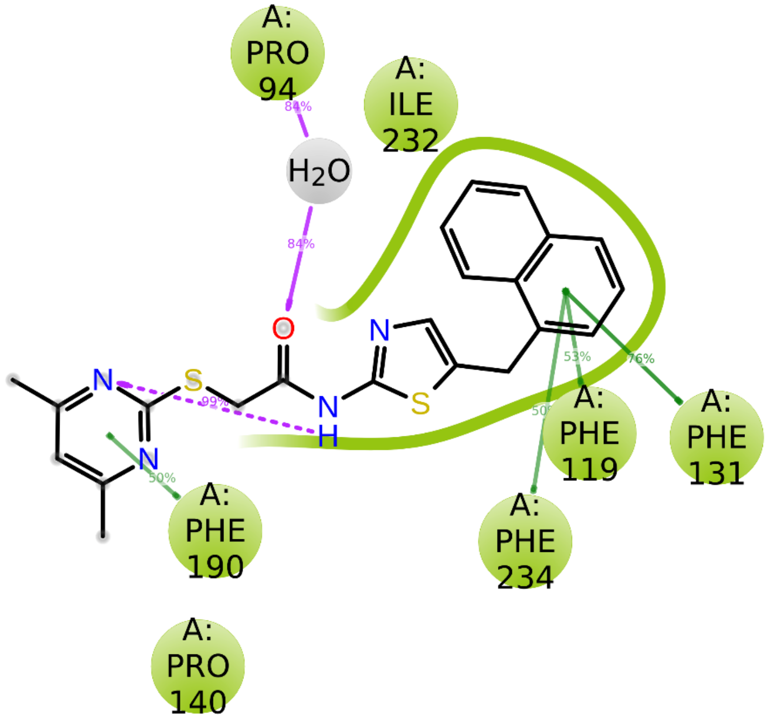

Figure S16. 2D protein-ligand interaction maps extracted for the SirReal2 inhibitor in the selective pocket of Sirt2. Herein, hydrogen bond (pink arrows), π-π (green lines), and hydrophobic (green) interactions are captured for respective complexes at 30% of the total molecular dynamics simulation. Images were rendered using academic Schrödinger-Maestro v12.4 suite[43] (URL: https://www.schrodinger.com/freemaestro).

S1.4. Post-molecular dynamics simulation analysis

S1.*4.1. Essential dynamics analysis*

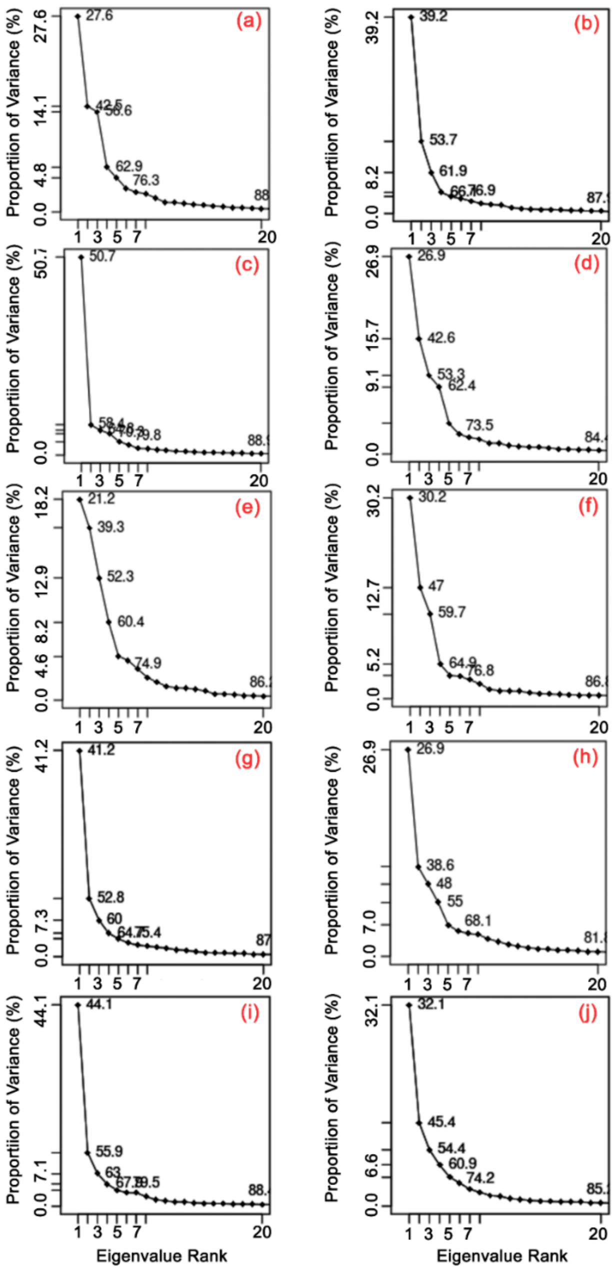

Figure S17. The percentage of total mean square displacement of residue positional variations recorded in each dimension is categorized by equivalent eigenvalue (PCs) for SIRT2 docked with

screened drugs, i.e. (a) Canagliflozin, (b) Flibanserin, (c) Ezetimibe, (d) Pimozide, (e) Fluphenazine, (f) Droperidol, (g) Osimertinib, (h) Pioglitazone, (i) Formoterol, and (j) Nintedanib.

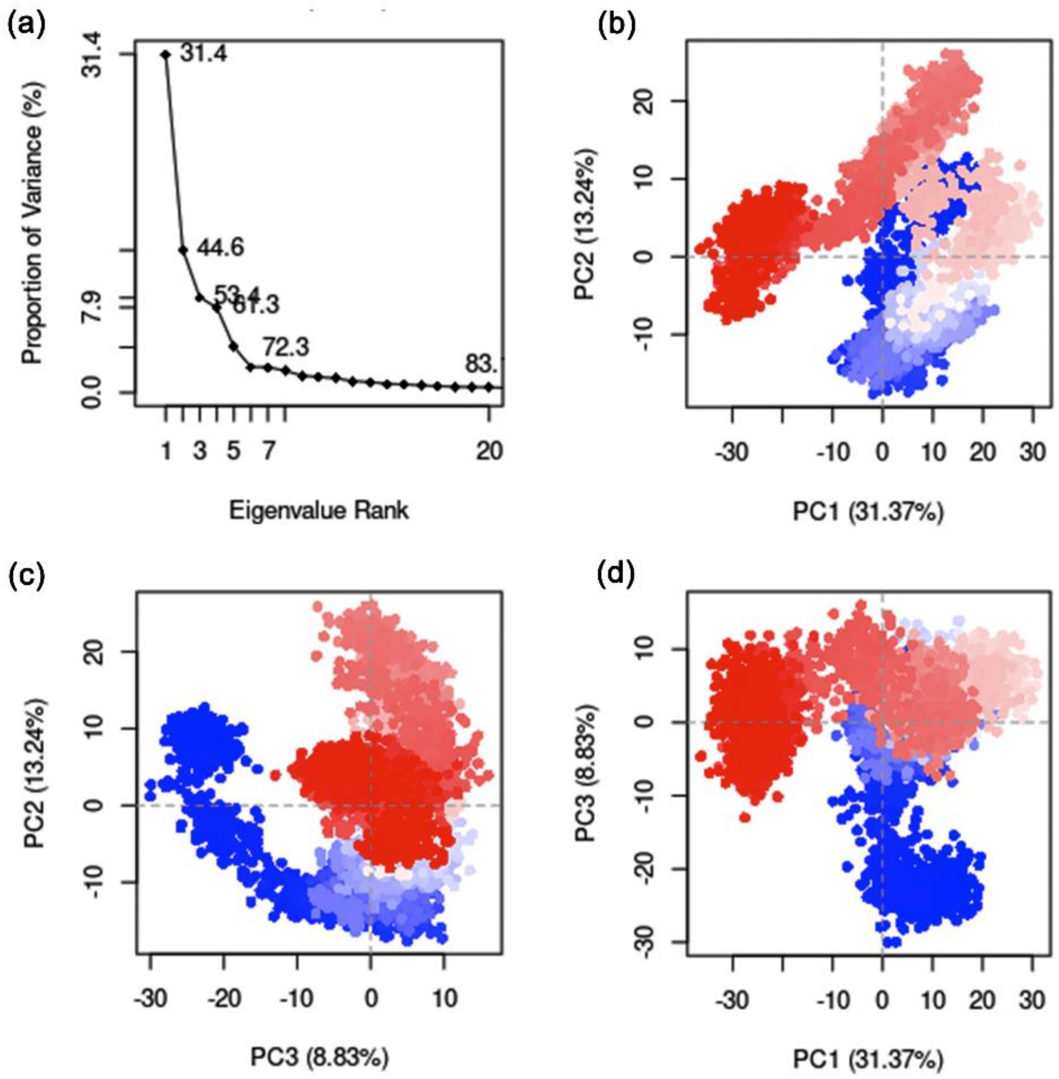

Figure S18. Essential Dynamics analysis for the reference complex SIRT2-SirReal2 molecular simulation trajectory; (a) Total percentage of mean square displacement in SIRT2 residues position logged in each direction are grouped by equivalent eigenvalue (PCs) for the Cα carbon atoms extracted from 100 ns simulation interval, (c-d) first three principal components extracted and plotted using 5000 frame shots from 100 ns molecular dynamics simulations of SIRT2-SirReal2 complex. In 2D plots, the incessant color scale from blue to white to red directs the periodic jumps between the structural poses of SIRT2 as function of 100 ns simulation interval.

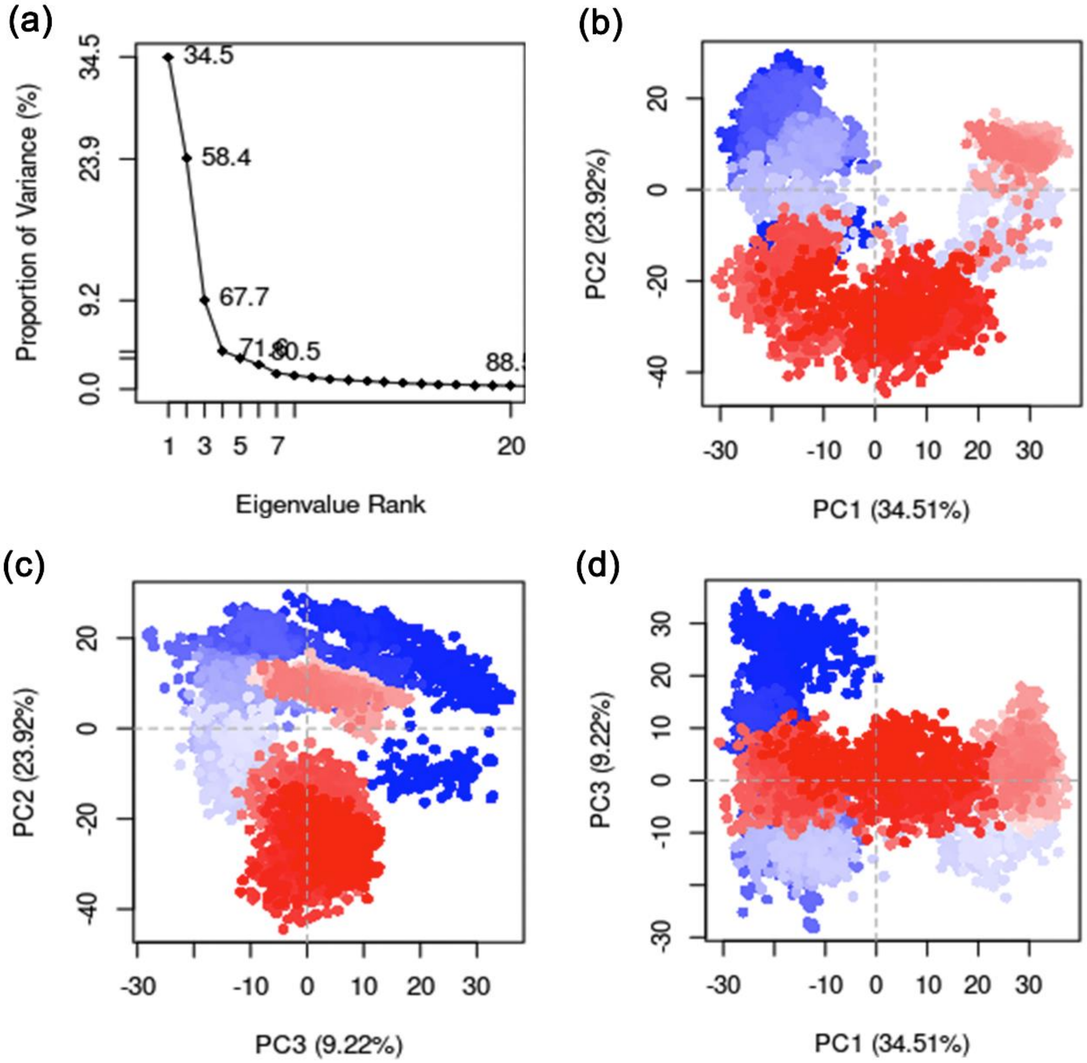

Figure S19. Essential Dynamics analysis for free SIRT2 protein molecular simulation trajectory; (a) Total percentage of mean square displacement in SIRT2 residues position logged in each direction are grouped by equivalent eigenvalue (PCs) for the Cα carbon atoms extracted from 100 ns simulation interval, (c-d) first three principal components extracted and plotted using 5000 frame shots from 100 ns molecular dynamics simulations of free SIRT2 protein. In 2D plots, the incessant color scale from blue to white to red directs the periodic jumps between the structural poses of SIRT2 as function of 100 ns simulation interval.

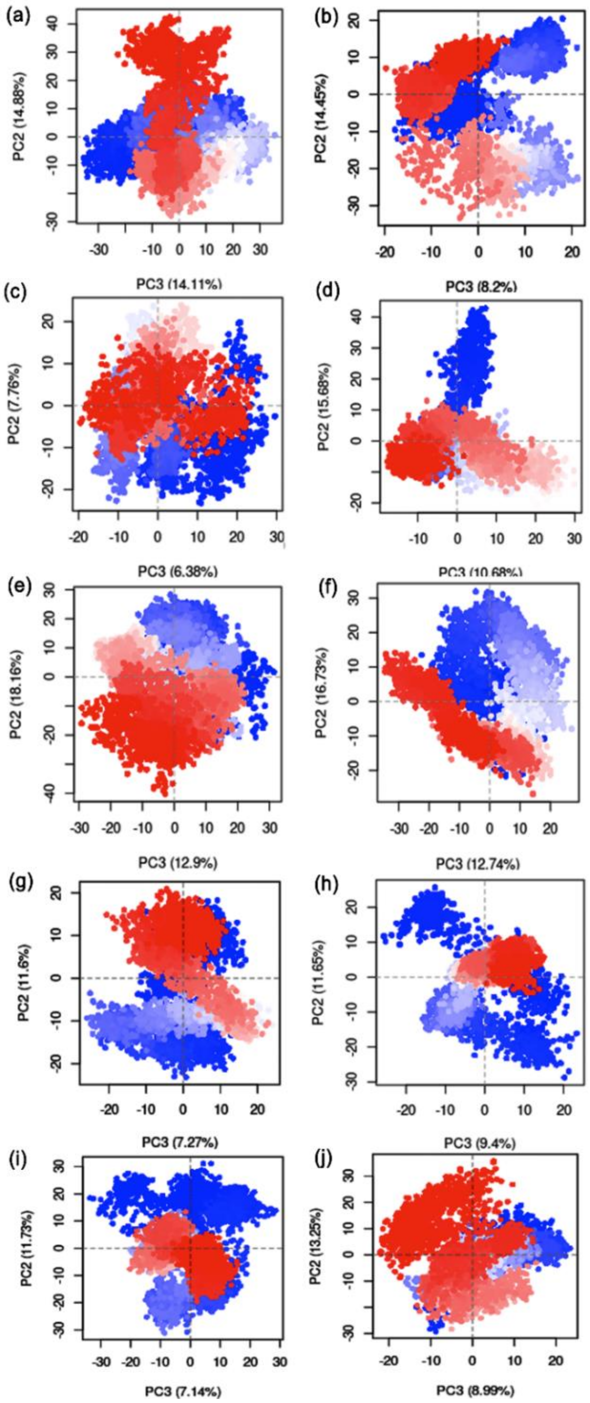

Figure S20. Principal components (PC2 vs PC3) analysis for 100 ns MD simulation trajectories of SIRT2 docked with selected drugs, i.e. (a) Canagliflozin, (b) Flibanserin, (c) Ezetimibe, (d) Pimozide, (e) Fluphenazine, (f) Droperidol, (g) Osimertinib, (h) Pioglitazone, (i) Formoterol, and (j) Nintedanib. The incessant color scale from blue to white to red directs the periodic jumps between the structural poses of SIRT2 as function of 100 ns simulation interval.

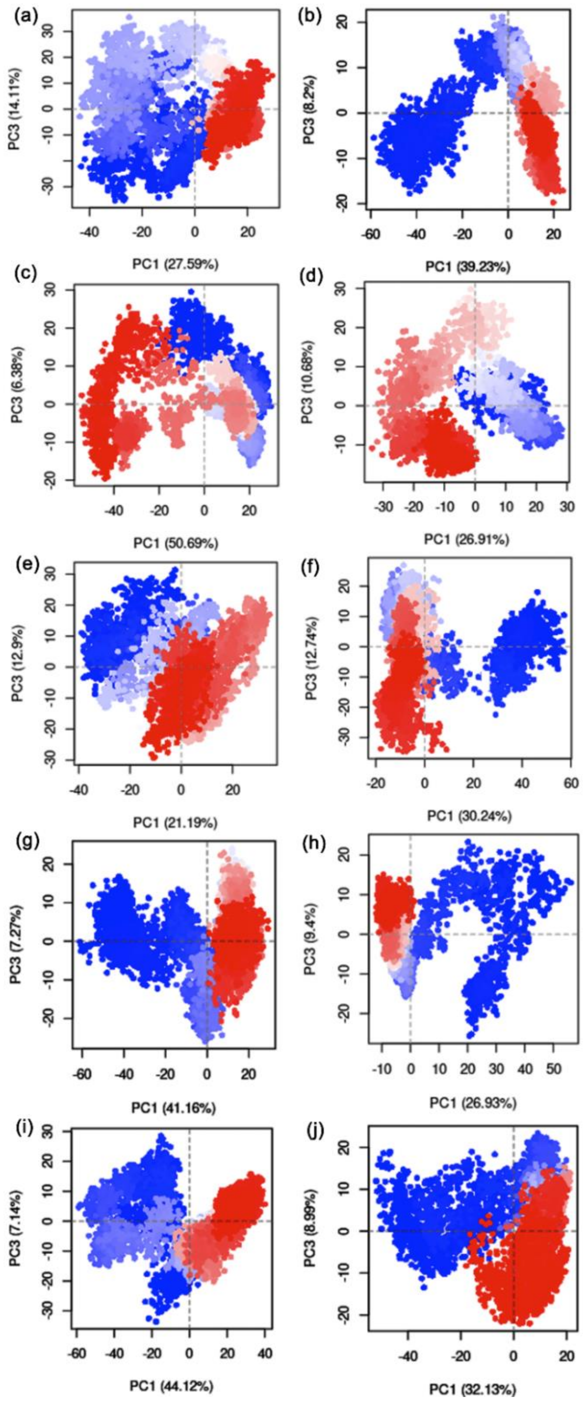

Figure S21. Principal components (PC3 vs PC1) analysis for 100 ns MD simulation trajectories of SIRT2 docked with selected drugs, i.e. (a) Canagliflozin, (b) Flibanserin, (c) Ezetimibe, (d) Pimozide, (e) Fluphenazine, (f) Droperidol, (g) Osimertinib, (h) Pioglitazone, (i) Formoterol, and (j) Nintedanib. The incessant color scale from blue to white to red directs the periodic jumps between the structural poses of SIRT2 as function of 100 ns simulation interval.

S1.4.2. End-point binding free energy calculation

Table S4. Molecular mechanics generalized Born surface area (MM/GBSA) binding free energy and energy dissociation components (kcal/mol) computed for the Sirt2 complexes with selected drugs against reference ligand on 100 ns MD simulation trajectories.

Components (kcal/mol)	Canagliflozin	Flibanserin	Ezetimibe	Pimozide	Fluphenazine	Droperidol	Osimertinib	Pioglitazone	Formoterol	Nintedanib	SirReal2
ΔG_{Bind}	-92.37±5.22	-74.1±5.118	-86.85±9.68	-92.96±6.54	-77.98±3.83	-43.32±4.98	-90.3±3.62	-86.74±6.56	-46.2±3.17	-105.58±7.28	-99.82±5.21
$\Delta G_{Bind\ Coulomb}$	-13.43±3.6	-53.46±4.7	-14.4±7.64	-33.37±3.05	-56.09±3.52	-44.17±4.61	-52.72±2.36	-8.38±2.52	-32.1±4.05	-49.19±1.64	-11.26±2.57
$\Delta G_{Bind\ Covalent}$	1.53±0.58	3.99±1.37	10.9±2.55	2.45±0.9	8.76±3.55	-0.09±2.36	4.29±2.91	4.12±0.71	1.91±1.92	4.11±1.45	2.512±0.81
$\Delta G_{Bind\ Hbond}$	-0.3±0.35	-0.97±0.17	-0.68±0.19	-0.05±0.06	-1.55±0.25	-1.55±0.22	-1.79±0.28	-0.22±0.35	-0.17±0.19	-1.11±0.13	-0.01±0.01
$\Delta G_{Bind\ Lipo}$	-44.68±1.61	-31.68±1.71	-44.66±2.02	-40.32±2.42	-30.92±1.52	-14.3±4.46	-31.5±1.38	-40.42±2.5	-21.22±1.33	-45.69±2.6	-43.62±2.04
$\Delta G_{Bind\ Packing}$	-4.15±0.39	-4.12±0.79	-6.65±0.73	-6.5±1.31	-2.94±0.99	-1.35±0.53	-7.54±0.81	-4.28±0.37	-0.72±0.54	-6.28±1.15	-6.66±0.64
$\Delta G_{Bind\ Solv\ GB}$	29.92±2.41	66.49±4.96	26.73±3.71	49.55±3.63	61.44±3.96	54.02±2.37	71.4±2.75	24.03±1.96	40.19±4.1	71.5±1.86	27.6±1.85
$\Delta G_{Bind\ vdW}$	-61.25±2.33	-54.34±2.82	-58.07±2.7	-64.71±2.56	-56.68±2.04	-35.86±2.25	-72.43±2.91	-61.58±3.49	-34.09±1.26	-78.92±4.08	-68.39±2.33

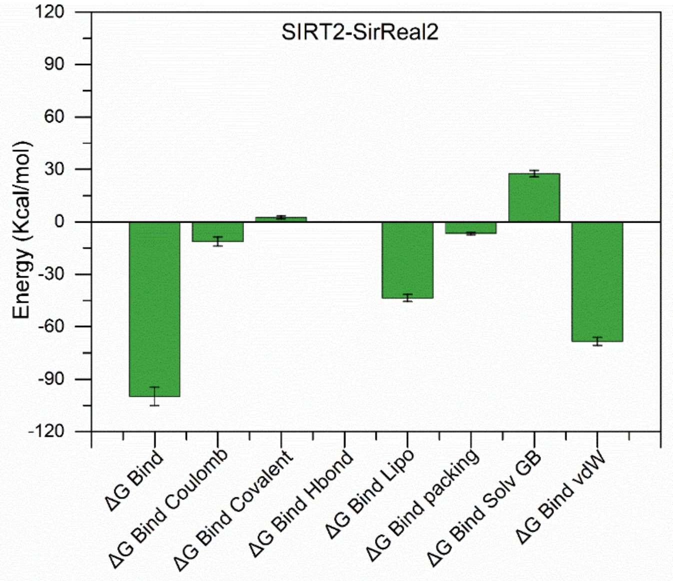

Figure S22. Graphical representation for the total Molecular mechanics generalized Born surface area (MM/GBSA) binding free energy and energy dissociation components were calculated for the molecular docked reference ligand in the selective pocket of Sirt2 from 100 ns MD simulation trajectory.

S1.5. 3D-QSAR models analysis

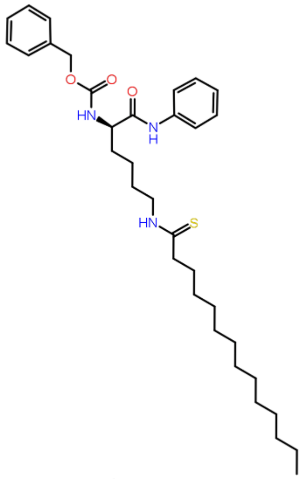

1. TM

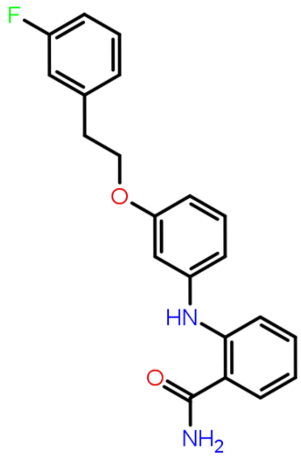

2. 7

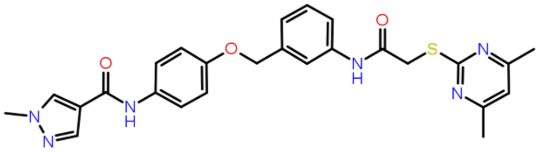

3. LC5

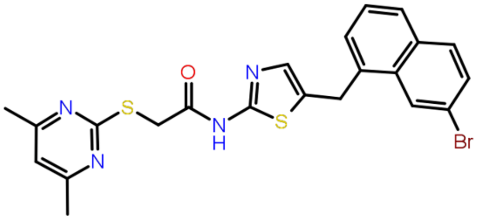

4. 5GN

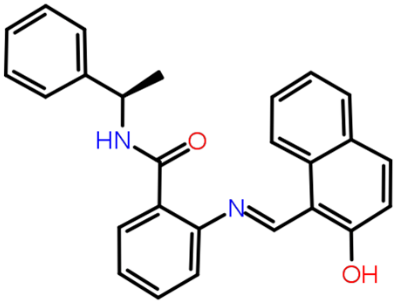

5. Sirtinol

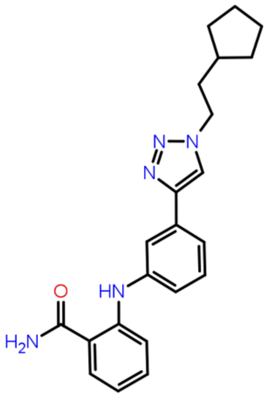

6. A1B11

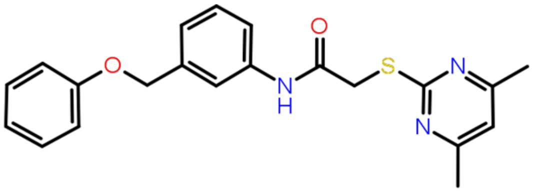

7. A21

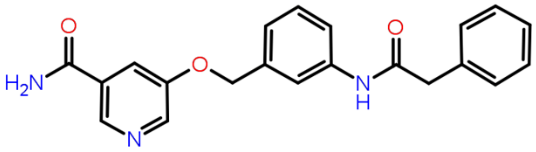

8. Compound 17

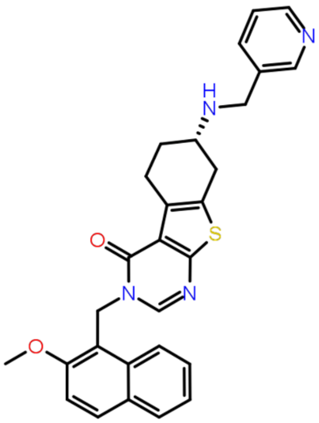

9. ICL-SIRT078

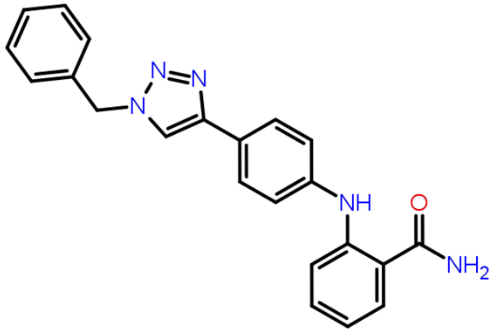

10. A2B57

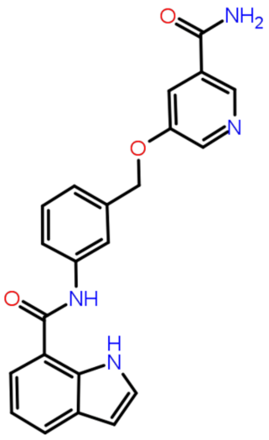

11. Compound 28

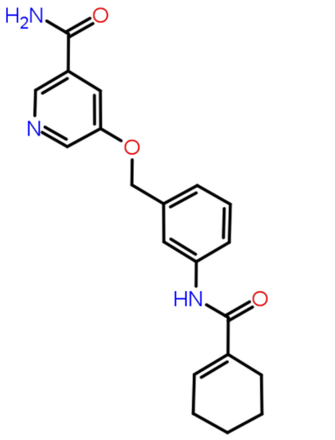

12. Compound 14

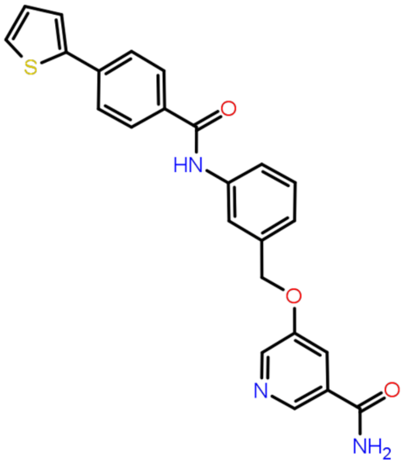

13. Compound 61

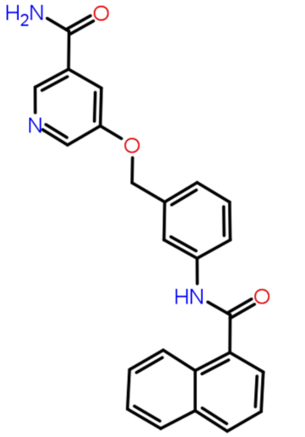

14. Compound 20

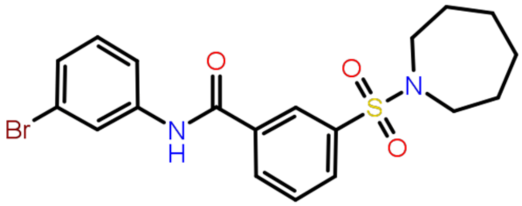

15. AK-7

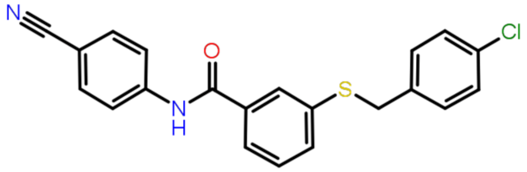

16. 16

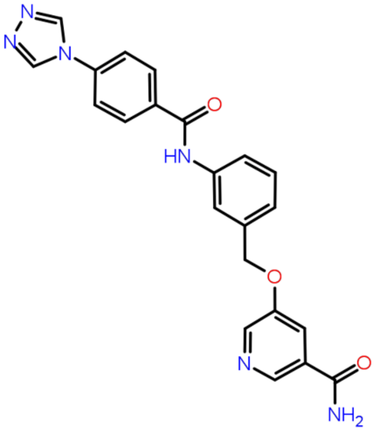

17. Compound 66

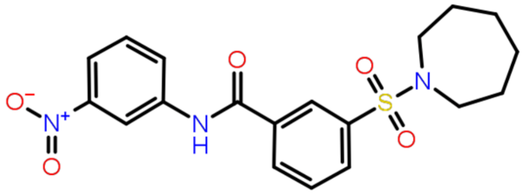

18. AK-1

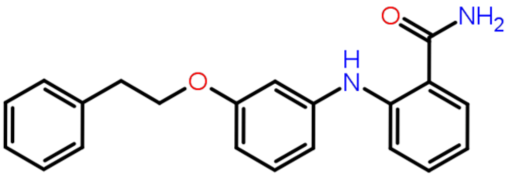

19. 7

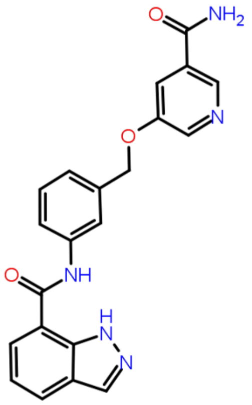

20. Compound 29

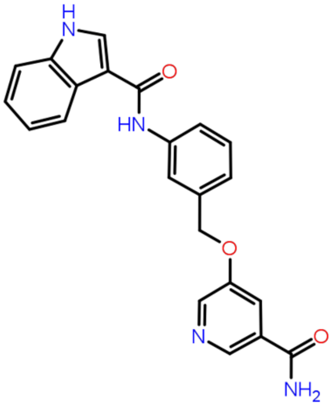

21. Compound 27

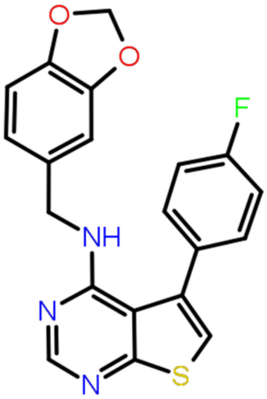

22. AEM1

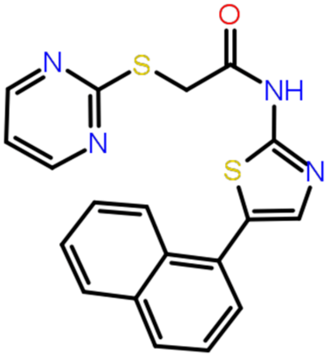

23. SirReal3

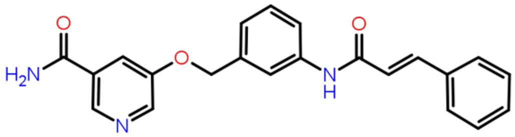

24. Compound 19

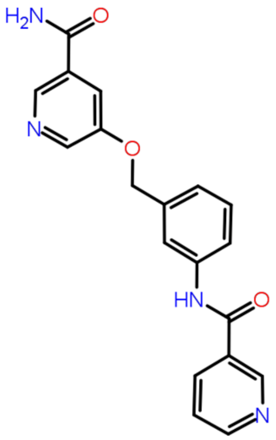

25. Compound 101

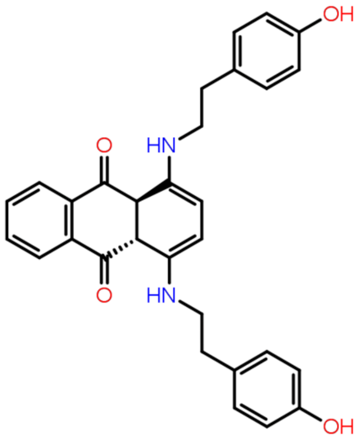

26. JED00244

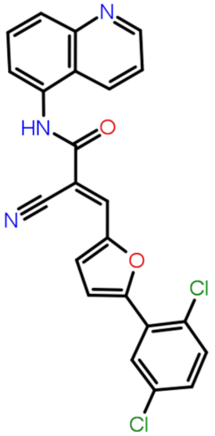

27. AGK2

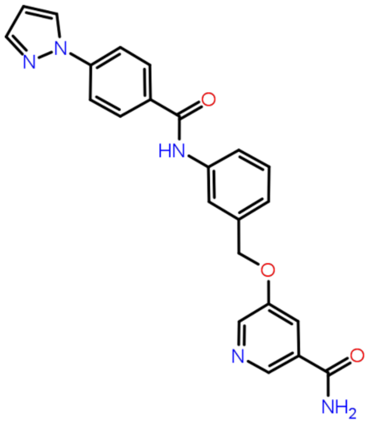

28. Compound 64

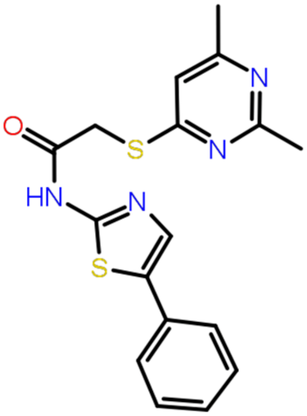

29. SirReal1

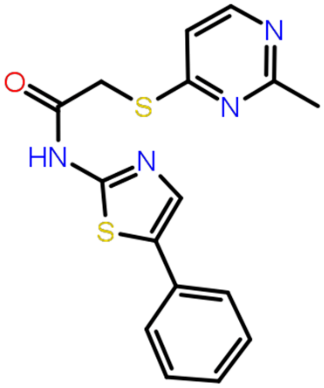

30. SirReal4

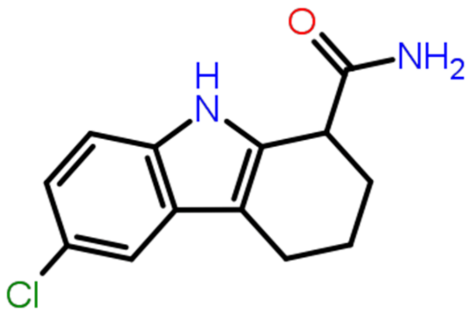

31. EX-527

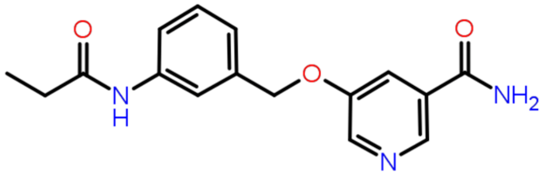

32. Compound 11

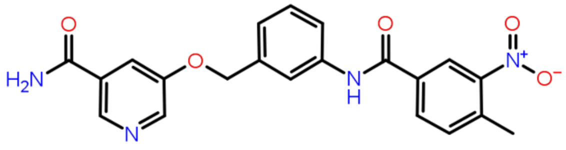

33. Compound 10

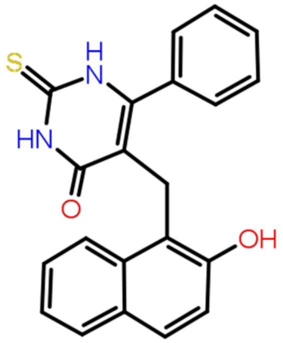

34. Cambinol

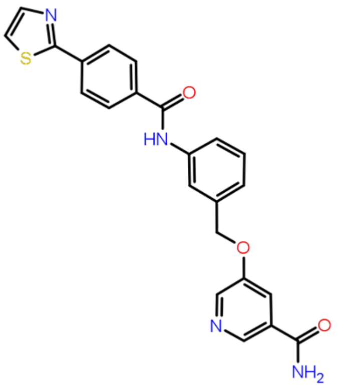

35. Compound 62

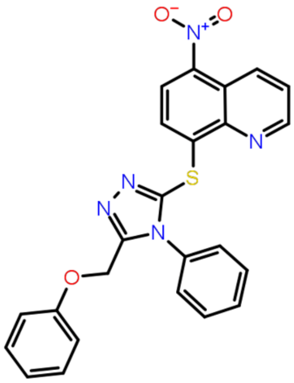

36. MIND4

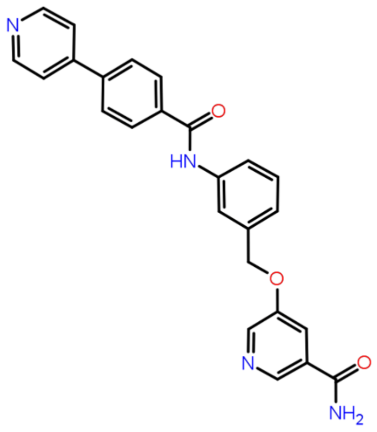

37. Compound 69

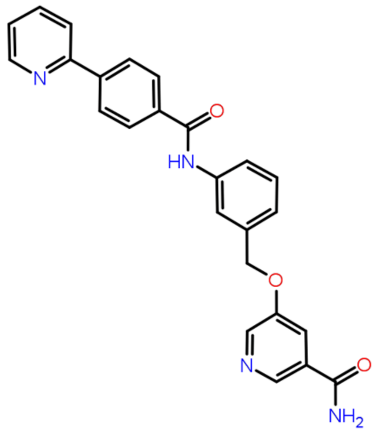

38. Compound 67

Figure S23. Structural formula and names as reported in the literature for the known selective inhibitors of Sirt2. 2D image is sketched using academic Schrödinger-Maestro v12.4 suite [43] (URL: https://www.schrodinger.com/freemaestro)

S.no.	Literature			This study		
	Compounds	IC50 (μM)	References	pIC50 (μM)	Docking score (kcal/mol)	RMSD (Å)
1	TM	0.028	[1]	7.552	-12.297	0.046
2	7	0.57	[1]	6.244	-12.061	0.046
3	24a (LC5)	0.815	[2]	6.089	-11.981	0.046
4	5GN	0.21	[3]	6.678	-11.741	0.043
5	Sirtinol	46	[1]	4.337	-11.639	0.036
6	A1B11	5.3	[4]	5.275	-11.382	0.049
7	1 (A21)	25.9	[2]	4.587	-11.375	0.047
8	Compound 17	4.8	[5]	5.319	-11.105	0.047
9	ICL-SIRT078	1.45	[6]	5.839	-10.927	0.042
10	A2B57	6.3	[4]	5.2	-10.903	0.045
11	Compound 28	0.0354	[5]	7.451	-10.897	0.047
12	Compound 14	0.546	[5]	6.263	-10.848	0.045
13	Compound 61	0.0263	[5]	7.58	-10.762	0.05
14	Compound 20	0.572	[5]	6.243	-10.758	0.043
15	AK-7	15.5	[1,7]	4.81	-10.738	0.039
16	16	2.9	[8]	5.538	-10.676	0.041
17	Compound 66	0.019	[5]	7.721	-10.609	0.041
18	AK-1	12.5	[7,9,10]	4.903	-10.582	0.034
19	7	1	[4]	6	-10.552	0.049
20	Compound 29	0.0157	[5]	7.804	-10.529	0.05
21	Compound 27	0.0831	[5]	7.08	-10.401	0.041
22	AEM1	18.5	[1]	4.733	-10.286	0.042
23	SirReal3	2.3	[11]	5.638	-10.238	0.049
24	Compound 19	0.156	[5]	6.807	-10.175	0.042
25	Compound 101	0.193	[5]	6.714	-10.147	0.044
26	JFD00244	56.7	[12]	4.246	-10.134	0.045
27	AGK2	3.5	[1]	5.455	-9.963	0.049
28	Compound 64	0.0447	[5]	7.35	-9.848	0.043
29	SirReal1	3.7	[11]	5.432	-9.716	0.037
30	SirReal4	16.8	[11]	4.77	-9.668	0.045
31	EX-527	20	[1,13]	4.699	-9.596	0.049
32	Compound 11	15.1	[5]	4.821	-9.021	0.048
33	Compound 10	0.107	[5]	6.97	-8.423	0.047
34	Cambinol	56	[14]	4.252	-8.17	0.048
35	Compound 62	0.046	[5]	7.58	-7.862	0.04
36	MIND4	3.5	[15]	5.456	-7.057	0.049
37	Compound 69	0.0229	[5]	7.64	-6.937	0.039
38	Compound 67	0.0227	[5]	7.644	-6.693	0.047

Table S5. List of known selective inhibitors of Sirt2 with names as reported in literature, IC50 values, pIC50 values, and docking score calculated at the selective pocket of Sirt2 protein.

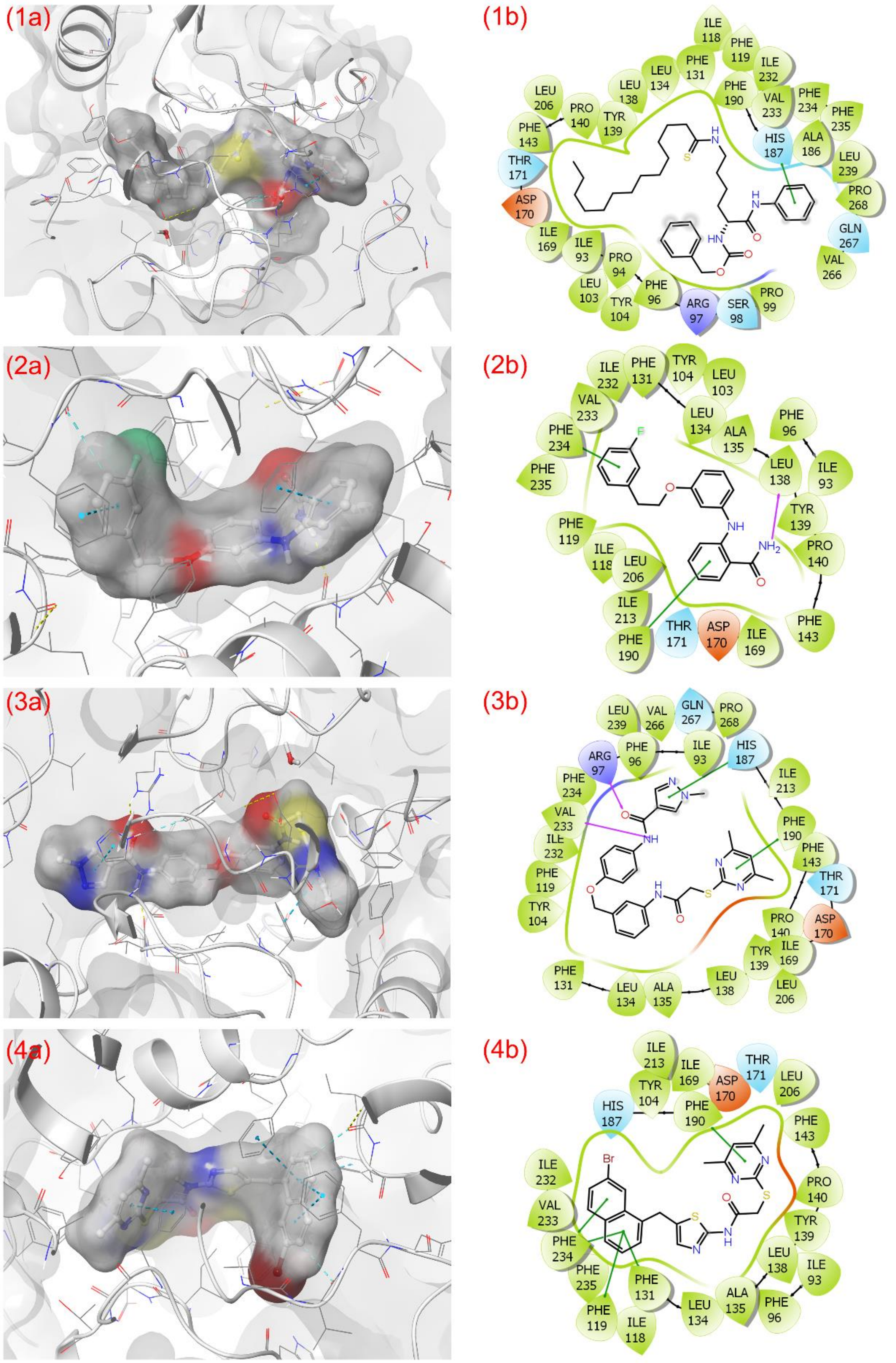

(1a)
(1b)
ILE 118
PHE 119
ILE 232
LEU 134
PHE 131
LEU 138
PHE 190
VAL 233
PHE 234
PHE 235
LEU 206
PRO 140
TYR 139
PHE 143
HIS 187
ALA 186
THR 171
LEU 239
ASP 170
PRO 268
GLN 267
ILE 169
ILE 93
PRO 94
VAL 266
LEU 103
PHE 96
TYR 104
ARG 97
SER 98
PRO 99
(2a)
(2b)
ILE 232
PHE 131
TYR 104
LEU 103
VAL 233
LEU 134
ALA 135
PHE 96
PHE 234
LEU 138
ILE 93
PHE 235
TYR 139
PHE 119
PRO 140
ILE 118
LEU 206
ILE 213
THR 171
ASP 170
ILE 169
PHE 143
PHE 190
(3a)
(3b)
LEU 239
VAL 266
GLN 267
PRO 268
ARG 97
PHE 96
ILE 93
HIS 187
PHE 234
ILE 213
VAL 233
PHE 190
ILE 232
PHE 143
PHE 119
THR 171
TYR 104
PRO 140
ASP 170
ILE 169
TYR 139
LEU 206
PHE 131
LEU 134
ALA 135
LEU 138
(4a)
(4b)
ILE 213
ILE 169
THR 171
TYR 104
ASP 170
LEU 206
HIS 187
PHE 190
PHE 143
ILE 232
PRO 140
VAL 233
TYR 139
PHE 234
LEU 138
ILE 93
PHE 235
PHE 131
ALA 135
PHE 96
PHE 119
ILE 118
LEU 134

(5a)

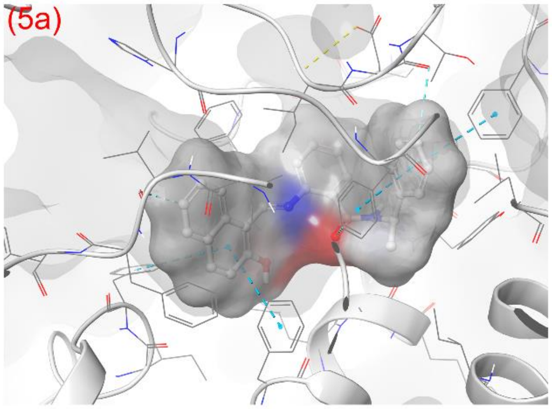

(5b)

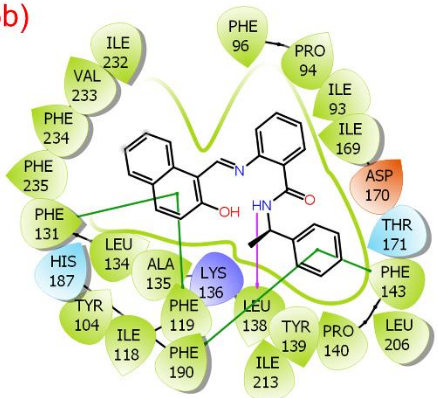

(6a)

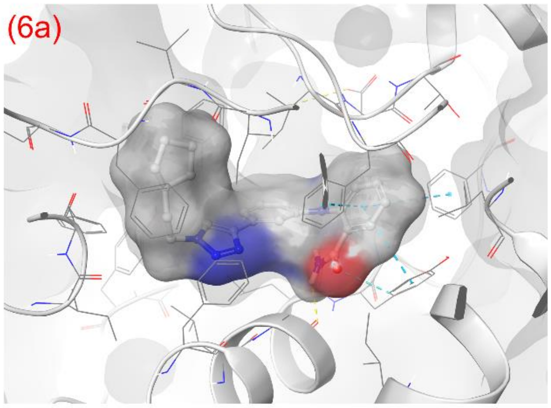

(6b)

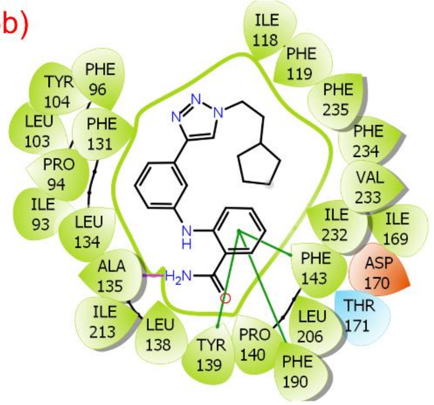

(7a)

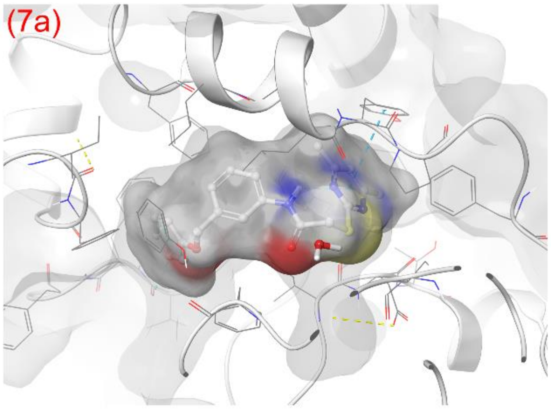

(7b)

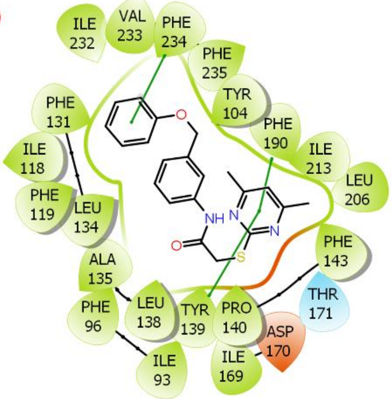

(8a)

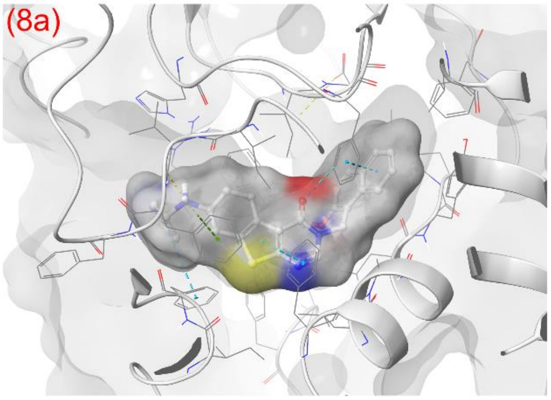

(8b)

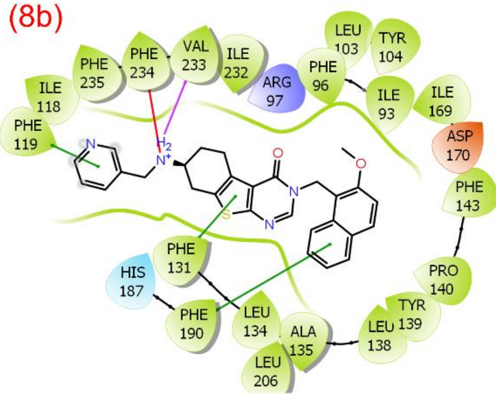

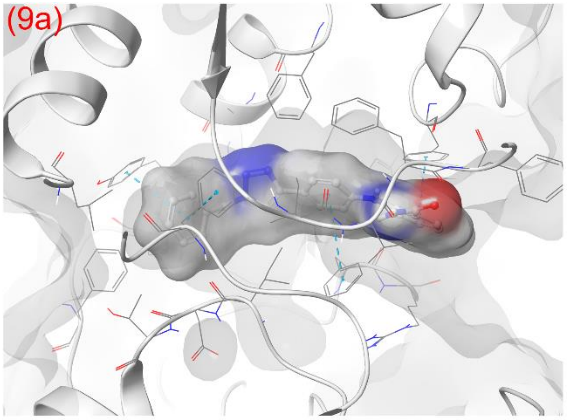
(9a)

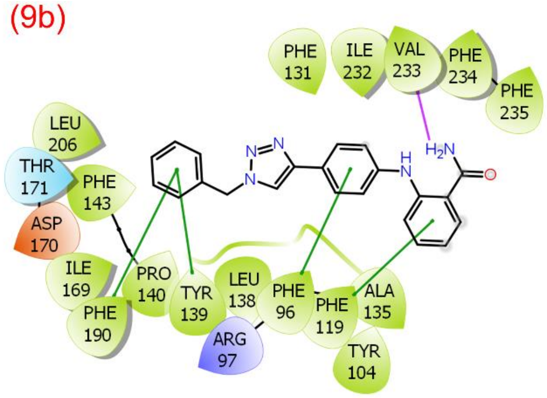
(9b)
PHE 131
ILE 232
VAL 233
PHE 234
PHE 235
LEU 206
THR 171
PHE 143
ASP 170
ILE 169
PHE 190
PRO 140
TYR 139
LEU 138
PHE 96
PHE 119
ALA 135
ARG 97
TYR 104
H2N

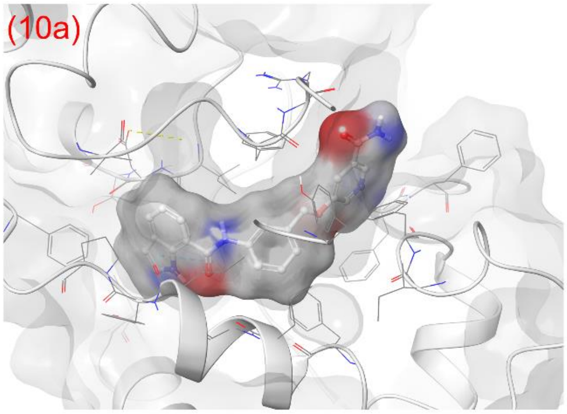
(10a)

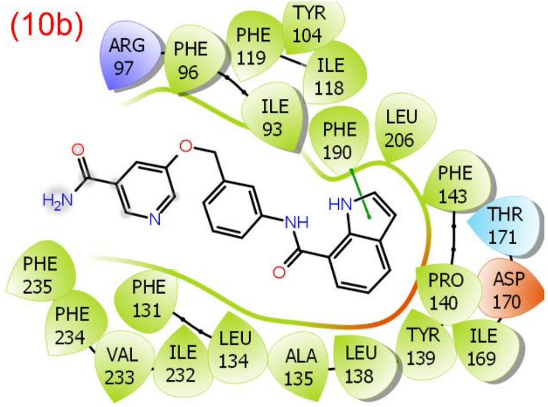
(10b)
ARG 97
PHE 96
PHE 119
TYR 104
ILE 118
ILE 93
PHE 190
LEU 206
PHE 143
THR 171
ASP 170
PRO 140
PHE 235
PHE 234
PHE 131
VAL 233
ILE 232
LEU 134
ALA 135
LEU 138
TYR 139
ILE 169
H2N
NH
HN

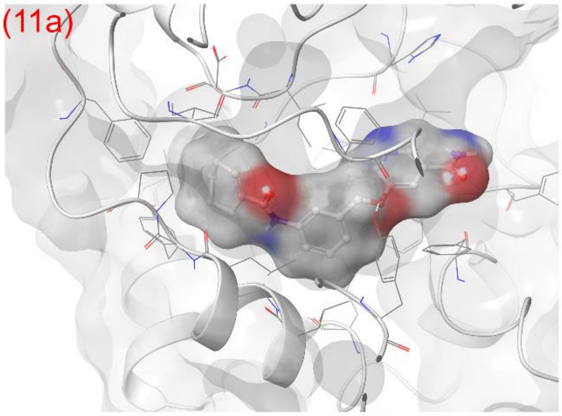
(11a)

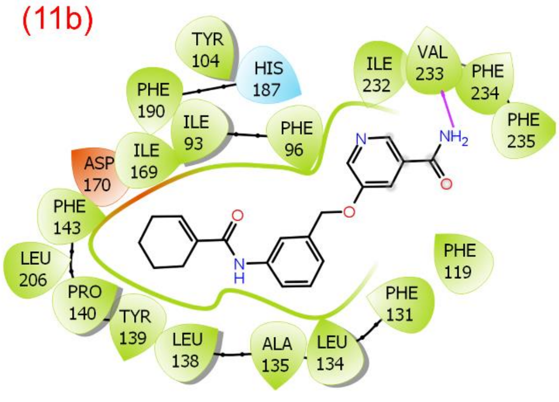
(11b)
TYR 104
HIS 187
PHE 190
ILE 93
PHE 96
ILE 232
VAL 233
PHE 234
PHE 235
ASP 170
ILE 169
PHE 143
LEU 206
PRO 140
TYR 139
LEU 138
ALA 135
LEU 134
PHE 131
PHE 119
NH2

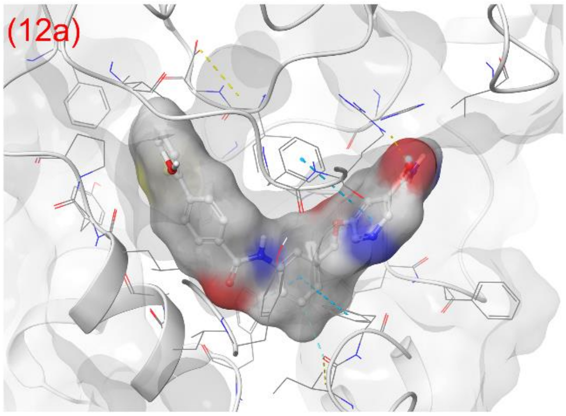
(12a)

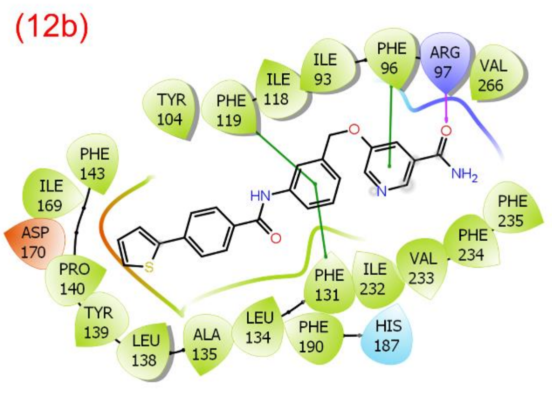
(12b)
ILE 93
PHE 96
ARG 97
VAL 266
TYR 104
PHE 119
ILE 118
PHE 143
ILE 169
ASP 170
PRO 140
TYR 139
LEU 138
ALA 135
LEU 134
PHE 190
HIS 187
PHE 131
ILE 232
VAL 233
PHE 234
PHE 235
NH2
HN

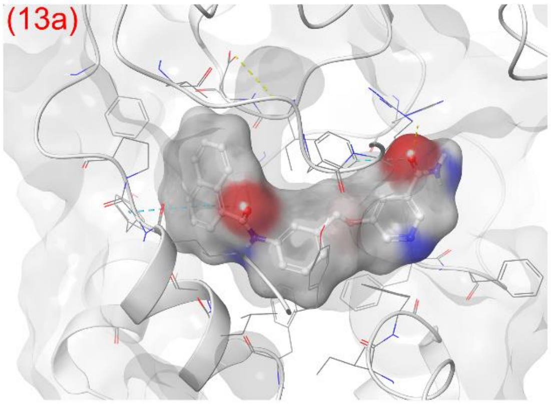
(13a)

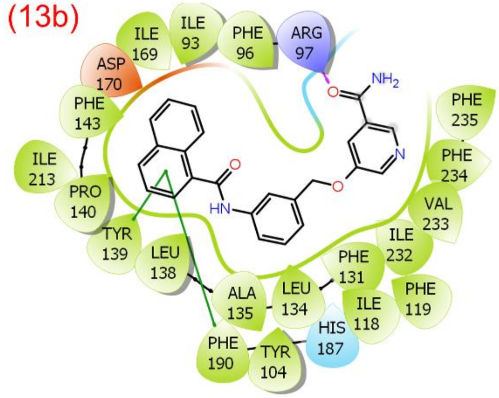
(13b)
ILE 169
ILE 93
PHE 96
ARG 97
ASP 170
PHE 143
ILE 213
PRO 140
TYR 139
LEU 138
ALA 135
LEU 134
PHE 131
ILE 232
VAL 233
PHE 234
PHE 235
PHE 119
ILE 118
HIS 187
TYR 104
PHE 190
NH2
O
N
HN

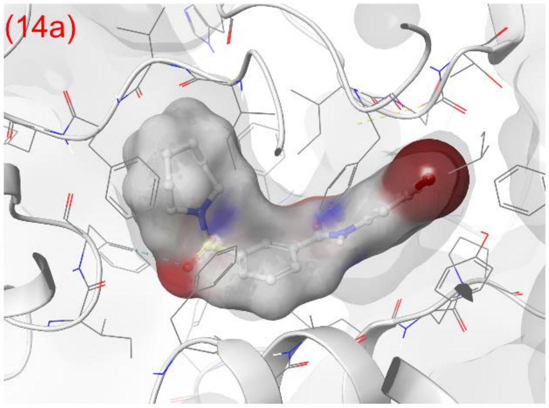
(14a)

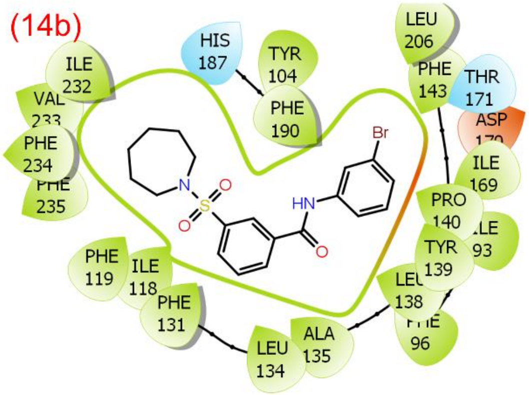
(14b)
HIS 187
TYR 104
PHE 190
LEU 206
PHE 143
THR 171
ILE 232
PHE 234
ILE 169
PRO 140
TYR 139
PHE 119
ILE 118
PHE 131
LEU 134
ALA 135
PHE 96
Br
N
S
O
HN

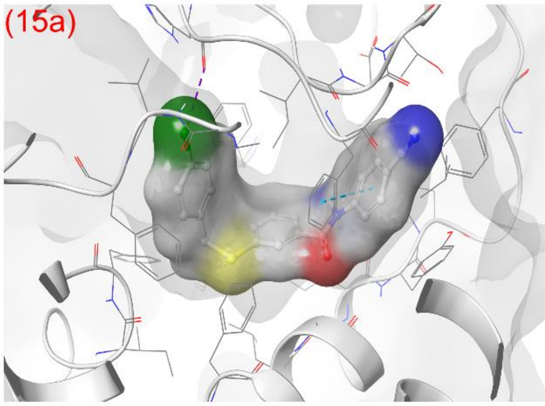
(15a)

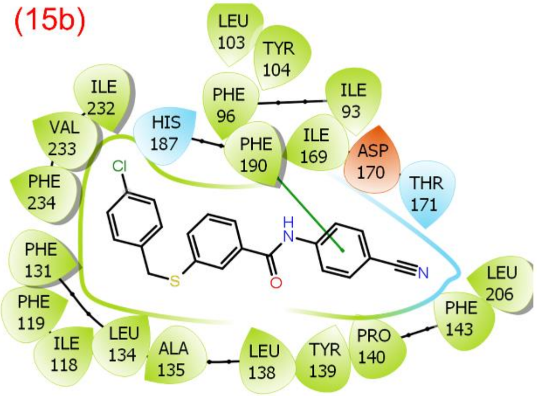
(15b)
LEU 103
TYR 104
ILE 232
PHE 96
ILE 93
VAL 233
HIS 187
PHE 190
ILE 169
ASP 170
THR 171
PHE 234
Cl
PHE 131
PHE 119
ILE 118
LEU 134
ALA 135
LEU 138
TYR 139
PRO 140
PHE 143
LEU 206
S
O
H
N

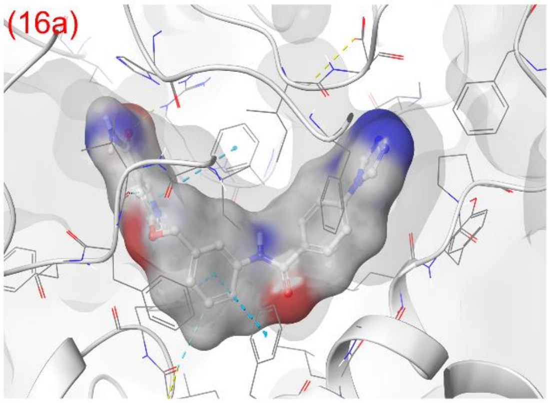
(16a)

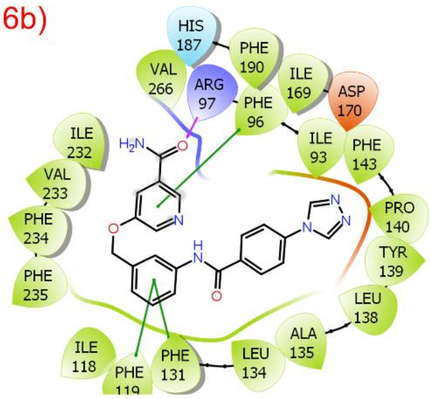
(16b)
HIS 187
PHE 190
VAL 266
ARG 97
ILE 169
ASP 170
PHE 96
ILE 232
ILE 93
PHE 143
VAL 233
PHE 234
PRO 140
TYR 139
PHE 235
LEU 138
ALA 135
ILE 118
LEU 134
PHE 131
H2N
O
N
H
N

(18a)

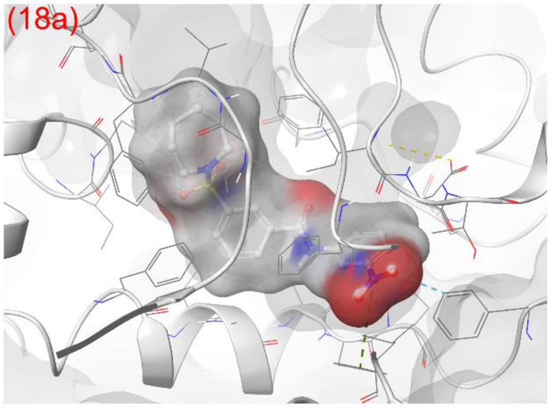

(18b)

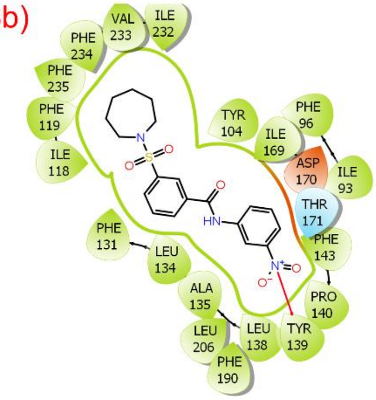

(19a)

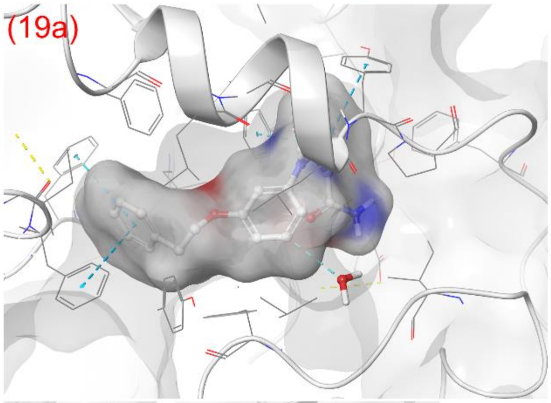

(19b)

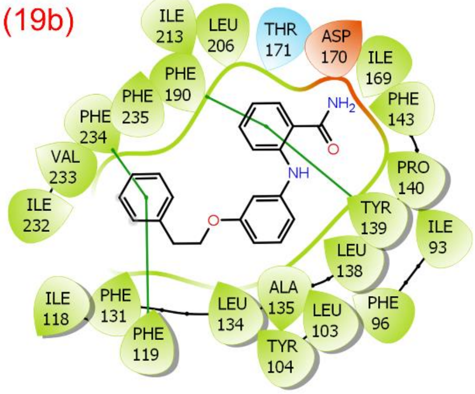

(20a)

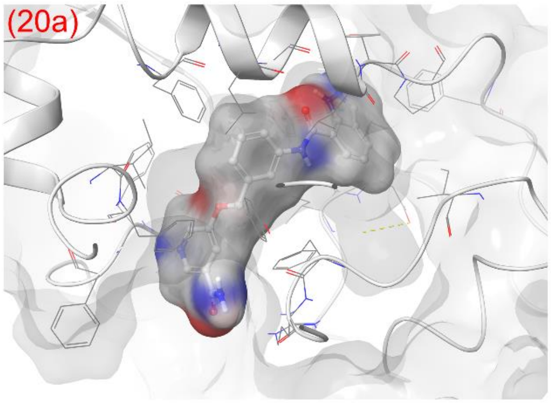

(20b)

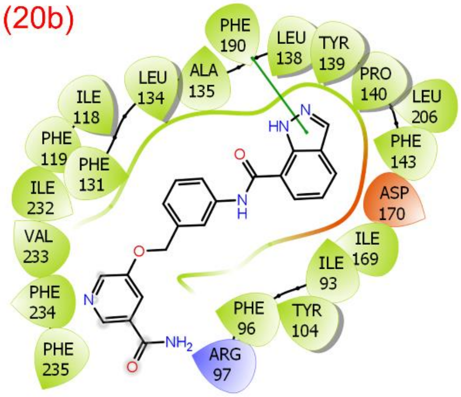

(21a)

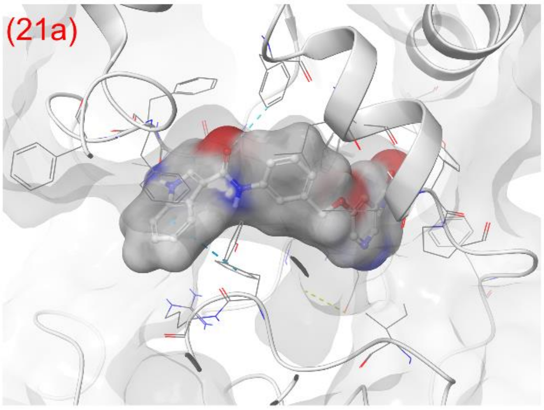

(21b)

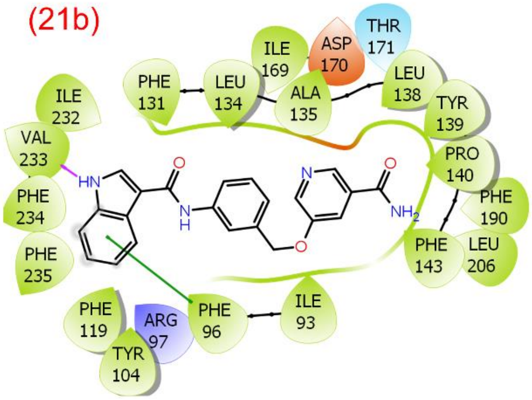

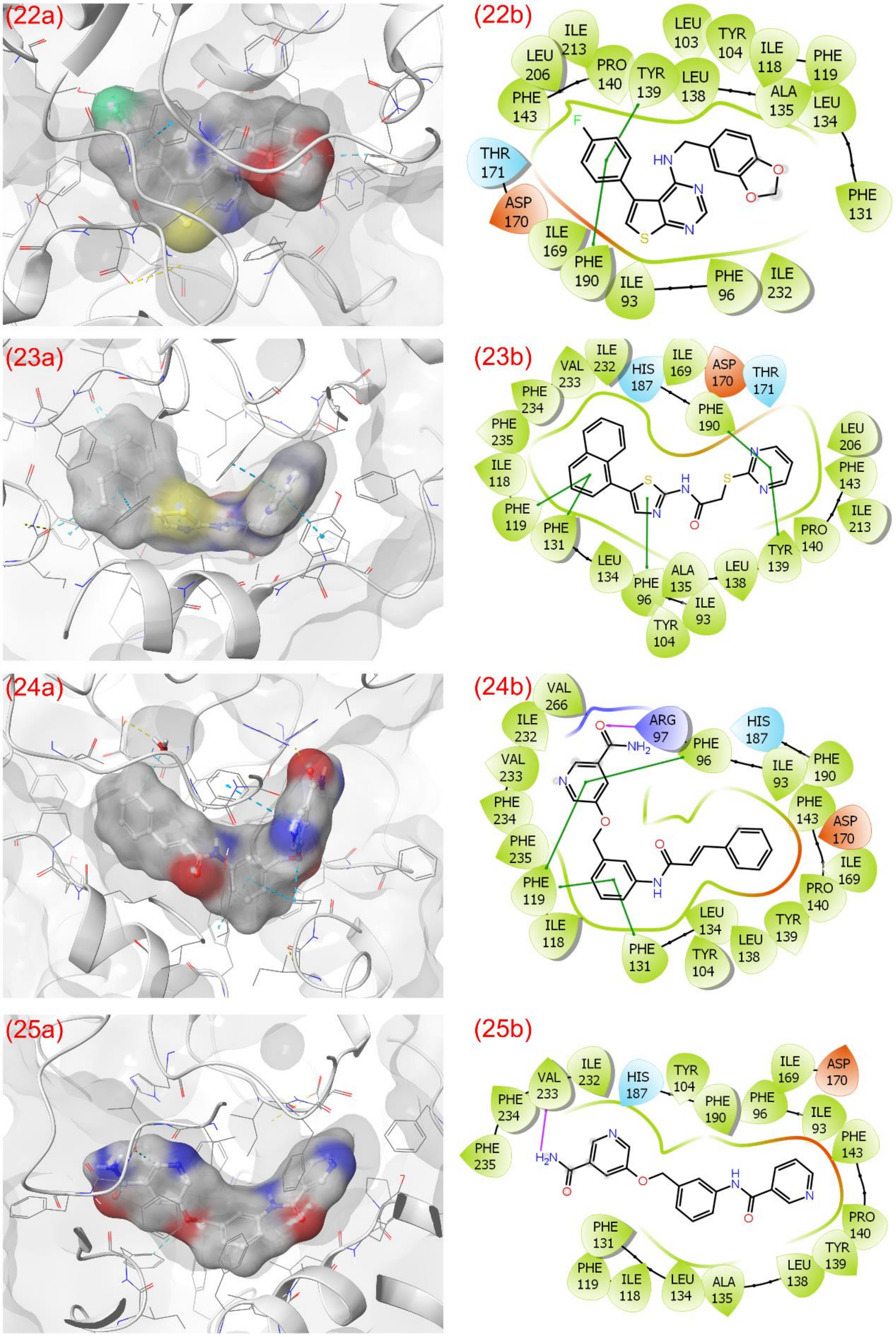
(22a)
(22b)
LEU 206
ILE 213
PRO 140
TYR 139
LEU 103
TYR 104
ILE 118
PHE 119
LEU 138
ALA 135
LEU 134
PHE 143
THR 171
ASP 170
PHE 131
ILE 169
PHE 190
ILE 93
PHE 96
ILE 232
(23a)
(23b)
VAL 233
ILE 232
HIS 187
ILE 169
ASP 170
THR 171
PHE 234
PHE 190
PHE 235
LEU 206
ILE 118
PHE 143
PHE 119
ILE 213
PHE 131
PRO 140
TYR 139
LEU 134
ALA 135
LEU 138
PHE 96
ILE 93
TYR 104
(24a)
(24b)
VAL 266
ILE 232
ARG 97
HIS 187
PHE 96
VAL 233
ILE 93
PHE 190
PHE 234
PHE 143
ASP 170
PHE 235
ILE 169
PRO 140
PHE 119
LEU 134
TYR 139
ILE 118
LEU 138
PHE 131
TYR 104
(25a)
(25b)
VAL 233
ILE 232
HIS 187
TYR 104
ILE 169
ASP 170
PHE 234
PHE 190
PHE 96
ILE 93
PHE 235
PHE 143
PRO 140
PHE 131
TYR 139
PHE 119
ILE 118
LEU 134
ALA 135
LEU 138

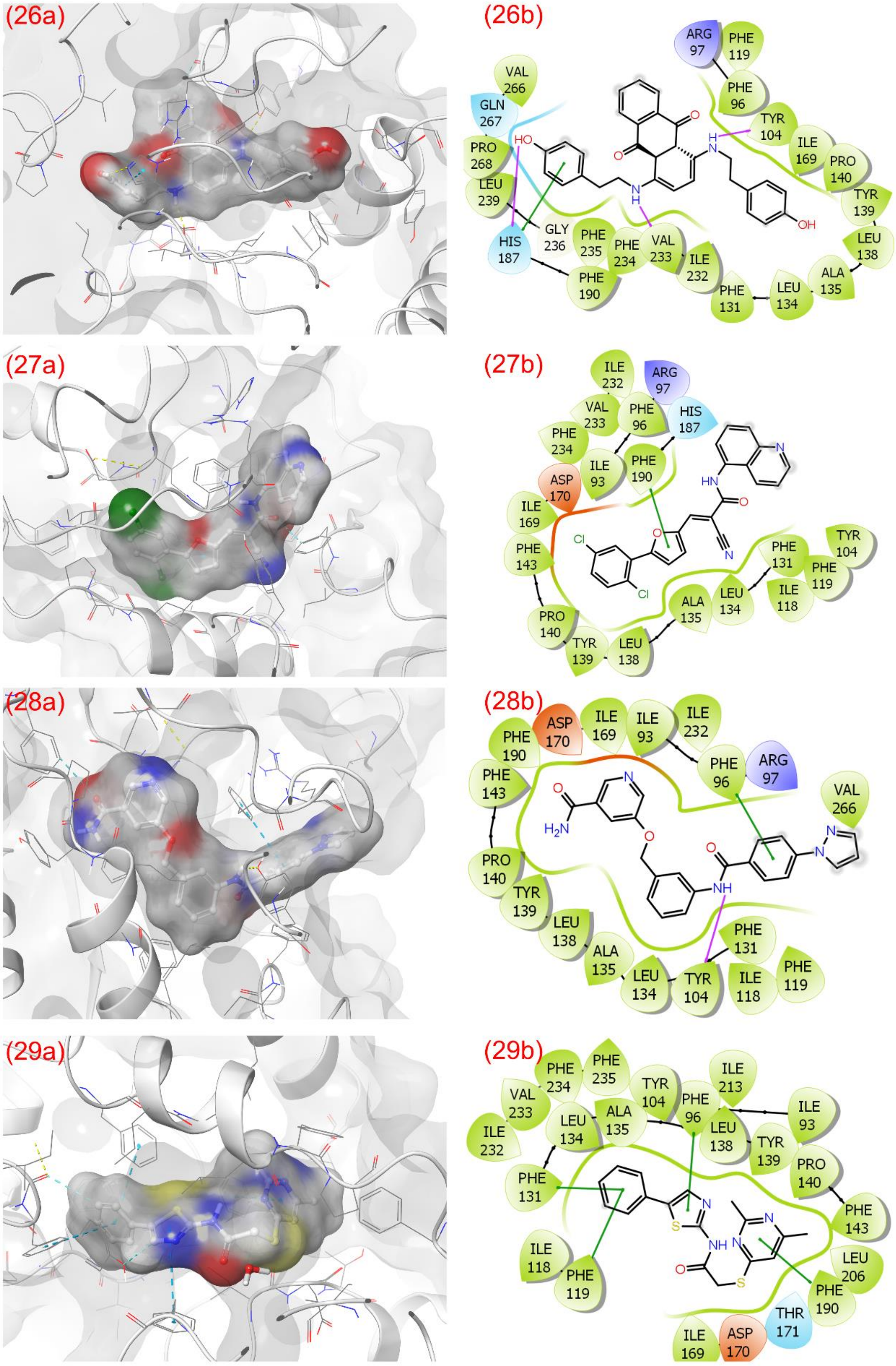
(26a)
(26b)
ARG 97
PHE 119
VAL 266
GLN 267
PHE 96
TYR 104
PRO 268
ILE 169
PRO 140
LEU 239
TYR 139
HIS 187
GLY 236
PHE 235
PHE 234
VAL 233
ILE 232
LEU 138
ALA 135
PHE 190
PHE 131
LEU 134
HO
O
O
H
N
N
H
OH
(27a)
(27b)
ILE 232
ARG 97
VAL 233
PHE 96
HIS 187
PHE 234
ILE 93
PHE 190
ASP 170
ILE 169
PHE 143
PHE 131
TYR 104
PHE 119
ILE 118
LEU 134
ALA 135
PRO 140
TYR 139
LEU 138
HN
N
O
N
O
Cl
Cl
(28a)
(28b)
ASP 170
ILE 169
ILE 93
ILE 232
PHE 190
PHE 96
ARG 97
PHE 143
VAL 266
PRO 140
TYR 139
LEU 138
ALA 135
LEU 134
TYR 104
PHE 131
ILE 118
PHE 119
H_2N
O
N
O
O
NH
N
N
(29a)
(29b)
PHE 234
PHE 235
ILE 213
VAL 233
TYR 104
PHE 96
ILE 93
ILE 232
LEU 134
ALA 135
LEU 138
TYR 139
PRO 140
PHE 131
PHE 143
ILE 118
PHE 119
LEU 206
PHE 190
THR 171
ILE 169
ASP 170
S
N
NH
N
N
O
S

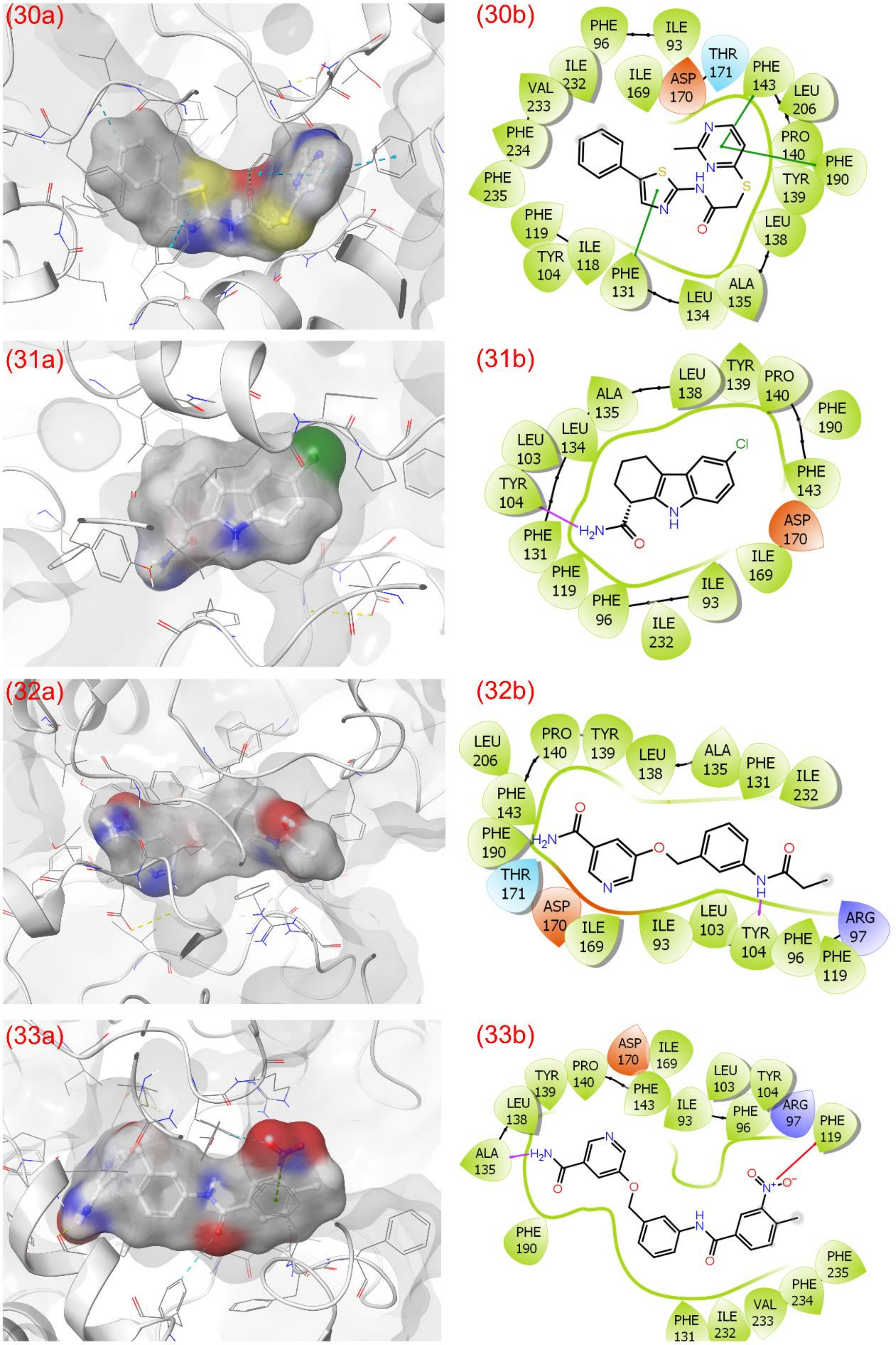
(30a)
(30b)
PHE 96
ILE 93
THR 171
ILE 232
ILE 169
ASP 170
PHE 143
LEU 206
VAL 233
PRO 140
PHE 234
PHE 190
PHE 235
TYR 139
PHE 119
LEU 138
TYR 104
ILE 118
PHE 131
ALA 135
LEU 134
(31a)
(31b)
LEU 138
TYR 139
PRO 140
ALA 135
PHE 190
LEU 103
LEU 134
PHE 143
TYR 104
ASP 170
PHE 131
ILE 169
PHE 119
ILE 93
PHE 96
ILE 232
(32a)
(32b)
LEU 206
PRO 140
TYR 139
LEU 138
ALA 135
PHE 131
ILE 232
PHE 143
PHE 190
THR 171
ASP 170
ILE 169
ILE 93
LEU 103
TYR 104
PHE 96
PHE 119
ARG 97
(33a)
(33b)
ASP 170
ILE 169
TYR 139
PRO 140
LEU 103
TYR 104
LEU 138
PHE 143
ILE 93
PHE 96
ARG 97
PHE 119
ALA 135
PHE 190
PHE 235
PHE 234
VAL 233
PHE 131
ILE 232

(34a)

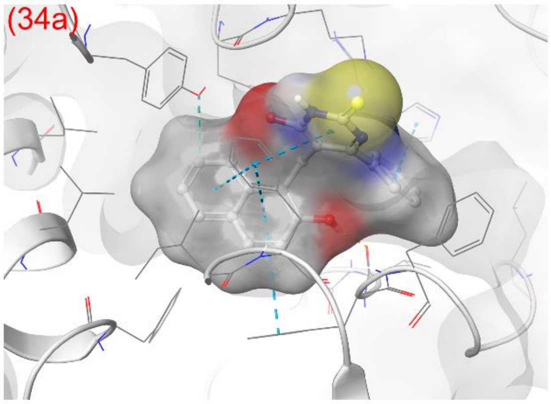

(34b)

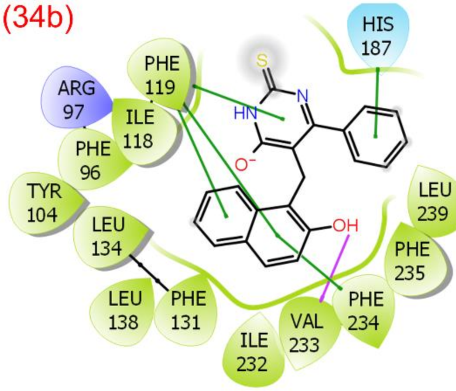

(35a)

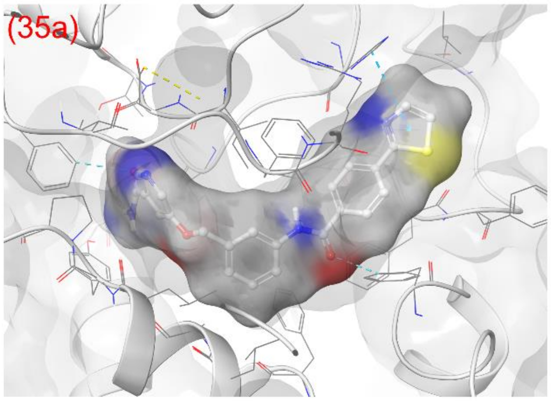

(35b)

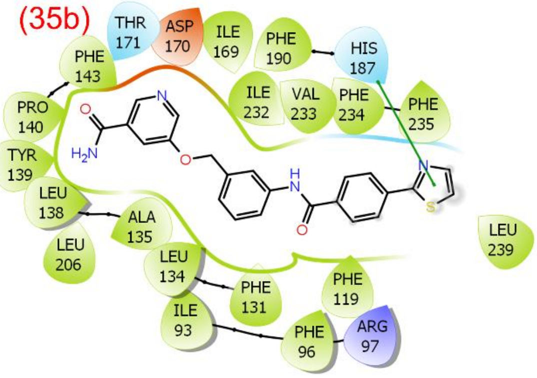

(36a)

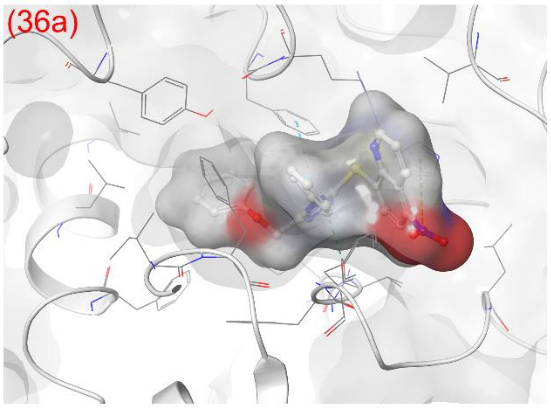

(36b)

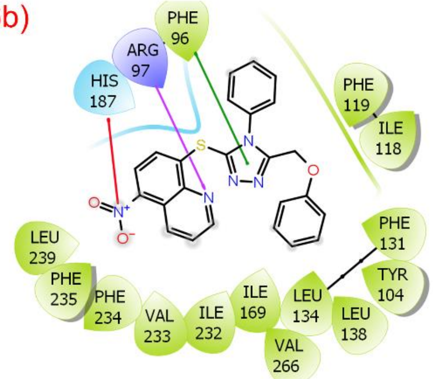

(37a)

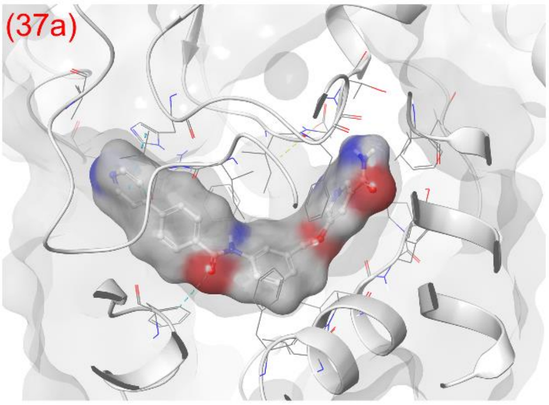

(37b)

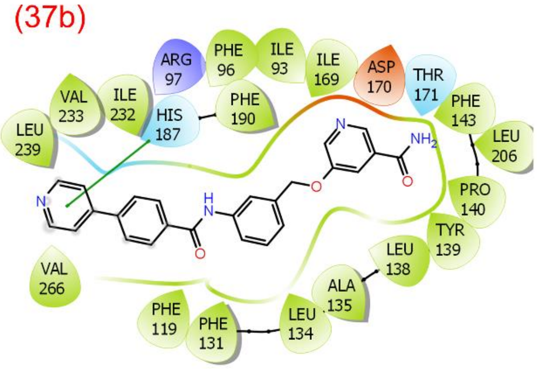

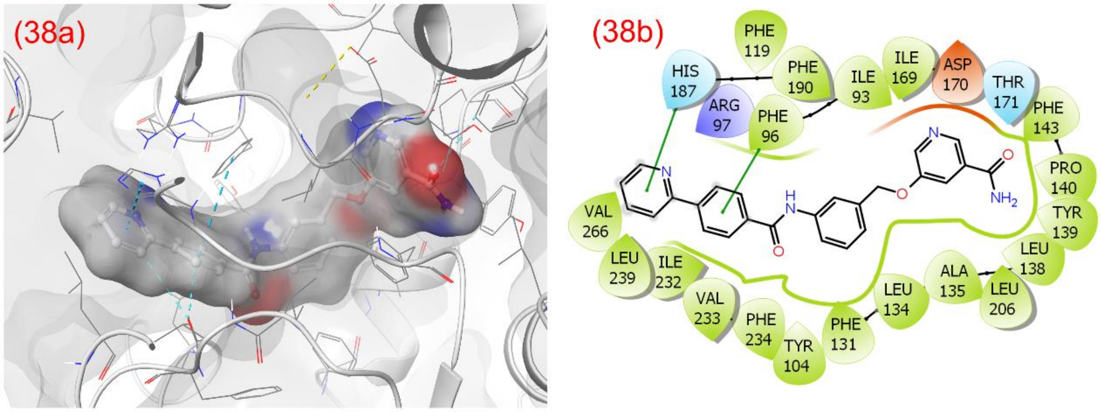

Figure S24. 3D and 2D intermolecular molecular contact for map for the docked complexes of known selective inhibitors of Sirt2, i.e. 1(a-b) TM, 2(a-b) 7, 3(a-b) 24a (LC5), 4(a-b) 5GN, 5(a-b) Sirtinol, 6(a-b)A1B11, 7(a-b) 1 (A21), 8(a-b) Compound 17, 9(a-b) ICL-SIRT078, 10(a-b) A2B57, 11(a-b) Compound 28, 12(a-b) Compound 14, 13(a-b) Compound 61, 14(a-b) Compound 20, 15(a-b) AK-7, 16(a-b) 16, 17(a-b) Compound 66, 18(a-b) AK-1, 19(a-b) 7, 20(a-b) Compound 29, 21(a-b) Compound 27, 22(a-b) AEM1, 23(a-b) SirReal3, 24(a-b) Compound 19, 25(a-b) Compound 101, 26(a-b) JFD00244, 27(a-b) AGK2, 28(a-b) Compound 64, 29(a-b) SirReal1, 30(a-b) SirReal4, 31(a-b) EX-527, 32(a-b) Compound 11, 33(a-b) Compound 10, 34(a-b) Cambinol, 35(a-b) Compound 62, 36(a-b) MIND4, 37(a-b) Compound 69, and 38(a-b) Compound 67, in the selective pocket of Sirt2. 2D interaction map were extracted at 4Å distance around the ligand in the selective pocket of Sirt2, where hydrogen bond (pink arrows), π-π (green lines), π-cation (red lines), hydrophobic (green), polar (blue), negative (red), positive (violet) and glycine (grey) interactions are depicted for respective docked complexes. Images were rendered using academic Schrödinger-Maestro v12.4 suite[43] (URL: https://www.schrodinger.com/freemaestro)

Table S6: Predicted activity data for test and training set of known selective inhibitors of Sirt2 using atom-based 3D-QSAR model.

S.no.	Ligand name	QSAR Set	PLS factor	Activity	Predicted Activity	Prediction error
1	JFD00244	training	5	4.246	4.29596	0.0495564
2	Cambinol	training	5	4.252	4.3968	0.144993
3	Sirtinol	training	5	4.337	4.23777	-0.0992334
4	1 (A21)	test	5	4.587	6.43858	1.85188
5	EX-527	training	5	4.699	4.51622	-0.182678
6	AEM1	training	5	4.733	4.66105	-0.0717471
7	SirReal4	test	5	4.77	5.54778	0.777777
8	AK-7	training	5	4.81	4.98792	0.178321
9	Compound 11	test	5	4.821	5.42141	0.600408
10	AK-1	training	5	4.903	4.91887	0.015872
11	A2B57	training	5	5.2	5.26431	0.0643091
12	A1B11	training	5	5.275	5.20833	-0.0666656
13	Compound 17	training	5	5.319	5.51469	0.195987
14	SirReal1	training	5	5.432	5.48447	0.0526838
15	AGK2	test	5	5.455	5.13198	-0.323025
16	MIND4	test	5	5.456	5.73095	0.275045
17	16	training	5	5.538	5.47427	-0.0633272
18	SirReal3	training	5	5.638	5.51966	-0.118335
19	ICL-SIRT078	training	5	5.839	5.90269	0.0640942
20	7	training	5	6	5.98024	-0.019762
21	24a (LC5)	test	5	6.089	7.04209	0.953293
22	Compound 20	training	5	6.243	6.48901	0.246406
23	7	training	5	6.244	6.04295	-0.201045
24	Compound 14	training	5	6.263	6.16786	-0.0949433
25	SirReal2	test	5	6.397	5.44885	-0.94815
26	5GN	test	5	6.678	5.55457	-1.12321
27	Compound 101	training	5	6.714	6.75793	0.0434861
28	Compound 19	training	5	6.807	6.92687	0.120073
29	Compound 10	training	5	6.97	6.99661	0.0266095
30	Compound 27	test	5	7.08	6.03078	-1.04962
31	Compound 64	training	5	7.35	7.47726	0.127573
32	Compound 28	training	5	7.451	7.07182	-0.379175
33	TM	training	5	7.552	7.60483	0.0528315
34	Compound 62	training	5	7.58	7.5265	-0.0535374
35	Compound 61	test	5	7.58	7.33968	-0.240363
36	Compound 69	training	5	7.64	7.68011	0.040107
37	Compound 67	training	5	7.644	7.5727	-0.0712673
38	Compound 66	training	5	7.721	7.72001	-0.0011862
39	Compound 29	test	5	7.804	7.10443	-0.699568

Table S7. Summary of PLS analysis results for the best common hypotheses (CPHs) generated using Atom-based 3D-QSAR model.

PLS Factors	SD	R^2	R^2 CV	R^2 Scramble	Stability	F	P	RMSE	Q^2	Pearson-r
1	0.4407	0.8621	0.7333	0.402	0.956	162.5	1.09E-12	0.86	0.372	0.6327
2	0.2967	0.9399	0.7857	0.5689	0.865	195.5	5.44E-16	0.91	0.3049	0.5623
3	0.228	0.9659	0.8007	0.7063	0.854	226.8	9.73E-18	0.9	0.3194	0.5701
4	0.1969	0.9756	0.7985	0.8211	0.855	230.4	3.41E-18	0.9	0.3153	0.5677
5	0.1471	0.987	0.8047	0.8721	0.828	334.2	5.65E-20	0.92	0.2846	0.5504

SD=standard deviation of the regression; R=squared value of R2 for the regression; F= variance ratio. Large values of F indicate a more statistically significant regression, P=significance level of variance ratio. Smaller values indicate a greater

degree of confidence; RMSE= root-mean-square error, Q^2=Q squared value of Q for the predicted activities, Pearson-r=Pearson r value for the correlation between the predicted and observed activity for the test set.

Table S8. Predicted activity data for test and training set of known selective inhibitors of Sirt2 using Field-based 3D-QSAR model.

S.no.	Ligand name	QSAR Set	PLS factor	Activity	Predicted Activity	Prediction error	% Extrapolated
1	1 (A21)	training	5	4.587	4.80412	0.217422	0.02
2	A2B57	training	5	5.2	5.15604	-0.0439644	0.03
3	A1B11	training	5	5.275	5.62894	0.353939	0.02
4	16	training	5	5.538	5.31791	-0.219688	0.01
5	7	training	5	6.244	5.9395	-0.304496	0.01
6	7	training	5	6	5.90966	-0.0903396	0.03
7	5GN	training	5	6.678	6.86822	0.190441	0.04
8	TM	training	5	7.552	7.65356	0.101563	0.47
9	Sirtinol	test	5	4.337	5.605	1.268	3.56
10	SirReal4	training	5	4.77	5.22953	0.459526	0.01
11	SirReal3	test	5	5.638	5.92742	0.28942	4.92
12	SirReal2	training	5	6.397	5.96141	-0.435593	0.04
13	SirReal1	training	5	5.432	5.24903	-0.182756	0.01
14	MIND4	test	5	5.456	6.56829	1.11239	18.5
15	24a (LC5)	training	5	6.089	5.82169	-0.26711	0.04
16	JFD00244	test	5	4.246	5.57708	1.33068	8.17
17	ICL-SIRT078	training	5	5.839	5.90857	0.0699691	0.06
18	EX-527	training	5	4.699	4.67617	-0.0227346	0.26
19	Compound 69	test	5	7.64	7.60567	-0.0343318	2.05
20	Compound 101	test	5	6.714	7.20059	0.48615	5.47
21	Compound 67	training	5	7.644	7.73956	0.0955881	0.01
22	Compound 66	training	5	7.721	7.55082	-0.170381	0.04
23	Compound 64	test	5	7.35	7.62566	0.275969	0.5
24	Compound 62	training	5	7.58	7.58904	0.00900219	0.01
25	Compound 61	training	5	7.58	7.48721	-0.0928334	0
26	Compound 29	training	5	7.804	7.46031	-0.343691	0.01
27	Compound 28	training	5	7.451	7.45899	0.00799518	0.04
28	Compound 27	training	5	7.08	7.11073	0.03033	0.08
29	Compound 20	test	5	6.243	7.05757	0.814966	0.56
30	Compound 19	test	5	6.807	6.28696	-0.519843	3.03
31	Compound 17	test	5	5.319	6.61407	1.29537	1.14
32	Compound 14	training	5	6.263	6.67669	0.41389	0.05
33	Compound 11	test	5	4.821	6.10997	1.28897	4.84
34	Compound 10	training	5	6.97	7.20788	0.237885	0.02
35	Cambinol	training	5	4.252	4.18701	-0.0648022	0.14
36	AK-7	training	5	4.81	4.8667	0.0570955	0
37	AK-1	training	5	4.903	4.8972	-0.00579759	0.03
38	AGK2	training	5	5.455	5.48592	0.0309248	0.72
39	AEM1	training	5	4.733	4.70142	-0.0313847	0.02

Table S9. Summary of PLS analysis results for the best common hypotheses (CPHs) generated using 3D Field based 3D-QSAR model.

PLS Factors	SD	R^2	R^2 CV	R^2 Scramble	Stability	F	P	RMSE	Q^2	Pearson-r
1	0.6259	0.7136	0.5128	0.3433	0.932	64.8	1.58E-08	0.83	0.4484	0.829
2	0.4871	0.8332	0.5765	0.5635	0.869	62.4	1.89E-10	0.81	0.4716	0.8761
3	0.395	0.8947	0.584	0.6519	0.815	68	7.10E-12	0.84	0.4342	0.8348
4	0.2905	0.9454	0.5915	0.735	0.715	99.6	3.55E-14	0.88	0.382	0.866
5	0.2416	0.9639	0.596	0.8072	0.67	117.4	4.18E-15	0.92	0.3205	0.8744

SD=standard deviation of the regression; R=squared value of R2 for the regression; F= variance ratio. Large values of F indicate a more statistically significant regression, P=significance level of variance ratio. Smaller values indicate a greater

degree of confidence; RMSE= root-mean-square error, Q,=squared value of Q2 for the predicted activities, Pearson-R=Pearson R value for the correlation between the predicted and observed activity for the test set.

Reference

1 Yang, L. L. *et al.* Discovery of 2-((4,6-dimethylpyrimidin-2-yl)thio)-N-phenylacetamide derivatives as new potent and selective human sirtuin 2 inhibitors. *Eur J Med Chem* **134**, 230-241, doi:10.1016/j.ejmech.2017.04.010 (2017).

2 Yang, L. L. *et al.* X-ray crystal structure guided discovery of new selective, substrate-mimicking sirtuin 2 inhibitors that exhibit activities against non-small cell lung cancer cells. *Eur J Med Chem* **155**, 806-823, doi:10.1016/j.ejmech.2018.06.041 (2018).

3 Schiedel, M. *et al.* Aminothiazoles as Potent and Selective Sirt2 Inhibitors: A Structure-Activity Relationship Study. *Journal of Medicinal Chemistry* **59**, 1599-1612, doi:10.1021/acs.jmedchem.5b01517 (2016).

4 Tatum, P. R. *et al.* Identification of novel SIRT2-selective inhibitors using a click chemistry approach. *Bioorg Med Chem Lett* **24**, 1871-1874, doi:10.1016/j.bmcl.2014.03.026 (2014).

5 Ai, T., Wilson, D. J., More, S. S., Xie, J. S. & Chen, L. Q. 5-((3-Amidobenzyl)oxy)nicotinamides as Sirtuin 2 Inhibitors. *Journal of Medicinal Chemistry* **59**, 2928-2941, doi:10.1021/acs.jmedchem.5b01376 (2016).

6 Ye, X. *et al.* Sirtuins in glucose and lipid metabolism. *Oncotarget* **8**, 1845-1859, doi:10.18632/oncotarget.12157 (2017).

7 Taylor, D. M. *et al.* A brain-permeable small molecule reduces neuronal cholesterol by inhibiting activity of sirtuin 2 deacetylase. *ACS Chem Biol* **6**, 540-546, doi:10.1021/cb100376q (2011).

8 Khanfar, M. A. *et al.* Design and Evaluation of 3-(Benzylthio)benzamide Derivatives as Potent and Selective SIRT2 Inhibitors. *Acs Med Chem Lett* **6**, 607-611, doi:10.1021/acsmedchemlett.5b00075 (2015).

9 Outeiro, T. F. *et al.* Sirtuin 2 inhibitors rescue alpha-synuclein-mediated toxicity in models of Parkinson's disease. *Science* **317**, 516-519, doi:10.1126/science.1143780 (2007).

10 Luthi-Carter, R. *et al.* SIRT2 inhibition achieves neuroprotection by decreasing sterol biosynthesis. *Proc Natl Acad Sci U S A* **107**, 7927-7932, doi:10.1073/pnas.1002924107 (2010).

11 Rumpf, T. *et al.* Selective Sirt2 inhibition by ligand-induced rearrangement of the active site. *Nat Commun* **6**, 6263, doi:10.1038/ncomms7263 (2015).

12 Tervo, A. J. *et al.* An in silico approach to discovering novel inhibitors of human sirtuin type 2. *J Med Chem* **47**, 6292-6298, doi:10.1021/jm049933m (2004).

13 Napper, A. D. *et al.* Discovery of indoles as potent and selective inhibitors of the deacetylase SIRT1. *Journal of Medicinal Chemistry* **48**, 8045-8054, doi:10.1021/jm050522v (2005).

14 Mahajan, S. S. *et al.* Development of pyrazolone and isoxazol-5-one cambinol analogues as sirtuin inhibitors. *J Med Chem* **57**, 3283-3294, doi:10.1021/jm4018064 (2014).

15 Quinti, L. *et al.* SIRT2- and NRF2-Targeting Thiazole-Containing Compound with Therapeutic Activity in Huntington's Disease Models. *Cell Chem Biol* **23**, 849-861, doi:10.1016/j.chembiol.2016.05.015 (2016).
